# Total Synthesis of Strigolactones via Palladium-Catalyzed Cascade Carbonylative Carbocyclization of Enallenes

**DOI:** 10.1021/acs.orglett.4c01283

**Published:** 2024-05-28

**Authors:** Bin Yang, Patrick Federmann, Viktoria Warth, Mingzhe Ren, Xin Mu, Haibo Wu, Jan-E. Bäckvall

**Affiliations:** † Department of Organic Chemistry, Arrhenius Laboratory, Stockholm University, SE-10691 Stockholm, Sweden; ‡ School of Chemistry, Xi’an Jiaotong University, Xi’an 710049, P. R. China

## Abstract

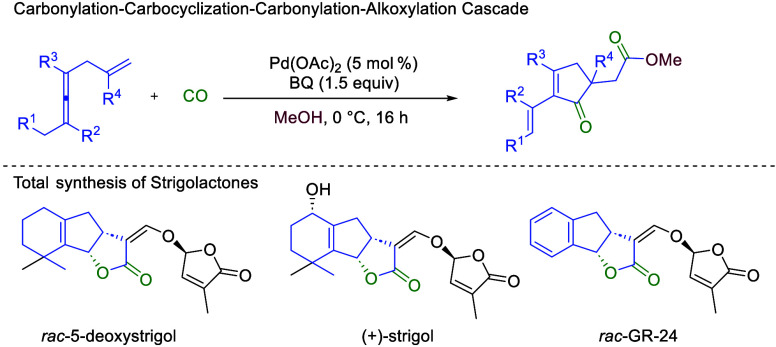

Here we report an efficient route for synthesizing strigolactones (SLs) and their derivatives. Our method relies on a palladium-catalyzed oxidative carbonylation/carbocyclization/carbonylation/alkoxylation cascade reaction, which involves the formation of three new C–C bonds and a new C–O bond while cleaving one C(sp^3^)–H bond in a single step. With our versatile synthetic strategy, both naturally occurring and artificial SLs were prepared.

Cascade reactions in organic synthesis are challenging and innovative but require delicate retrosynthetic strategies.^1−3^ In addition to their aesthetic appeal, cascade processes offer economical and environmentally friendly means for generating molecular complexity.^4,5^ Consequently, these reactions have found numerous applications in the synthesis of complex molecules, both natural and designed. Utilizing cascade reactions in the synthesis of natural products can be highly efficient and may produce remarkable outcomes.^6,7^

Functionalized allenes are valuable organic molecules that are significantly important in transition-metal catalysis. There are numerous examples of the use of functionalized allenes as starting materials in synthetic methodologies.^8−13^ All of these methodologies contribute to the accessibility of versatile building blocks, enabling the synthesis of complex molecular targets, including natural products, pharmaceuticals, and functional materials.^12,14−18^ In ongoing research by our team with the objective of developing palladium-catalyzed transformations of enallenes and evaluating their utility in total synthesis, we embarked on their applications in the synthesis of strigolactones (SLs). The isolation of strigol from the root exudates of cotton in 1966 was the first reported compound of this lactone family.^19^ Afterward, several research teams reported the total synthesis of SLs and their derivatives.^20−24^ However, acquiring SLs directly from nature or through existing total synthesis methods remains environmentally challenging and economically difficult. SLs play a crucial role for chemical signals that control various aspects of shoot and root growth in plants,^25−28^ and there is a growing need for these compounds among phytochemists and agricultural chemists.^29^ Previous studies have suggested that SLs enhance crop resistance to drought and high salinity, leading to increased crop yields, especially in arid regions, making them promising new-generation agrochemical compounds.^30−32^ However, traditional synthetic methods for obtaining SLs have proven to be unsatisfactory in terms of step efficiency and atom economy. More versatile synthesis pathways for obtaining naturally occurring and artificial SLs are therefore important for the development of phytochemistry and agricultural chemistry.

As Matusova et al. have indicated, SLs originate from open-chain unsaturated hydrocarbons, exemplified by 15-*cis*-phytoene in Scheme 1A.^33^ The transformation of the latter to the SL skeleton is characterized by oxidative cyclization, a process that forms multiple bonds in a single step with remarkable selectivity and efficiency.^33^ Building upon understanding of oxidative cyclization to form complex structures, we have developed a number of palladium(II)-catalyzed oxidative carbocyclizations of allenes with high chemo-, regio-, and stereoselectivity, which provide a set of useful structures and, more importantly, reveal completely new synthetic disconnections.^15,34−36^ These methods often involve multiple bond formation, giving an intrinsic advantage of step and atom economy.^37,38^

**Scheme 1:**
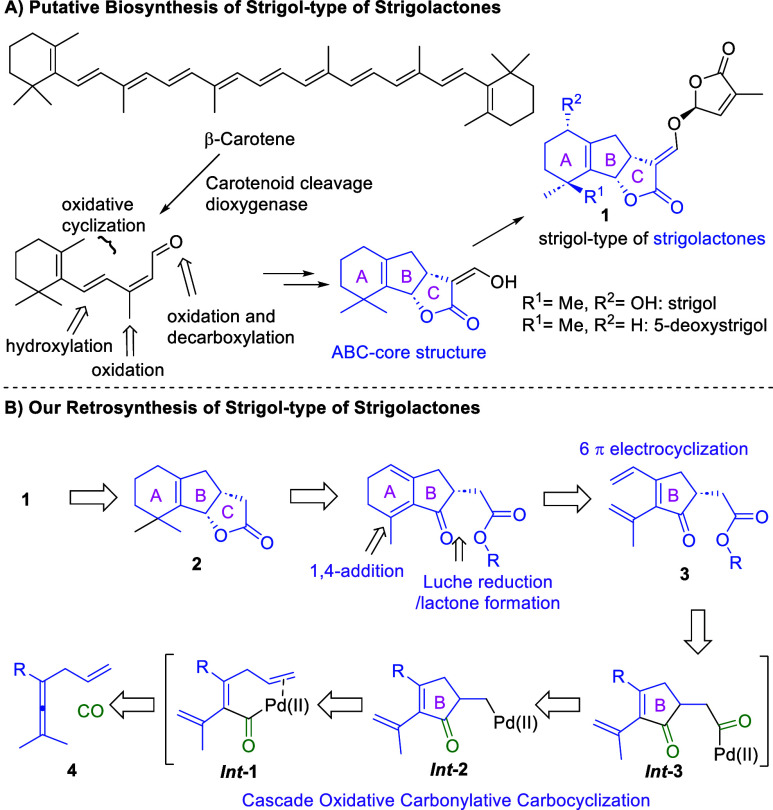
Biosynthetic Origins of SLs and Our Conceptual Route for the Chemical Synthesis of SLs

Inspired by our previously developed palladium(II)-catalyzed oxidative carbocyclization reactions, a possible route to SLs was designed (Scheme 1B). We anticipated that the B-ring moiety of the SLs could be constructed from an open-chain enallene via a cascade oxidative carbonylative carbocyclization. Here, ***Int*-1** is formed from **4** via activation of the allene by Pd(II) involving C–H bond cleavage to give a dienyl–Pd(II) intermediate followed by carbonylation. The electrocyclic reactions (both oxidative and non-oxidative) could be used to synthesize the A-ring moiety (Scheme 1B).^39^ Afterward, a 1,4-addition, a Luche reduction, and a lactone formation would lead to the C-ring moiety. This proposed route proceeds in a scalable and more concise fashion by applying a carbonylative cascade process in which four bonds are formed, and a bond is cleaved selectively in a one-pot manner. More importantly, several substitutions can be easily introduced into various positions at SLs by using the corresponding substituted enallenes as the substrate. The key part of this chemistry is to develop a catalytic pathway to transform enallenes **4** into cyclopentenones **3** (Scheme 1B), which can be further transformed into SLs. The selectivity control during the transformation is usually the main obstacle for the development of this methodology. Herein, we report the realization of this design, work that has resulted in an efficient synthesis of SLs and an SL derivative from readily accessible enallenes as starting materials. It is interesting to note that our synthetic strategy has similarities to the biogenesis of SLs suggested by Matusova et al.^33^

Our proposed route to SLs became possible only after the realization of the cascade oxidative carbonylative carbocyclization of enallenes **4** to cyclopentenones **3**. We initially chose readily accessible enallene **4a** as the model substrate (Table 1). When **4a** was treated with 5 mol % Pd(OAc)_2_ and 1.5 equiv of benzoquinone (BQ) under 1 atm of CO (balloon) in MeOH, a complicated mixture was generated (Table 1, entry 1). However, when DMSO was added (20 mol %), the reaction proceeded smoothly and the envisioned product **3a** was obtained in 76% yield (Table 1, entry 2). DMSO could serve as a key ligand in one or more steps in the catalytic cycle.^40^

**Table 1:**
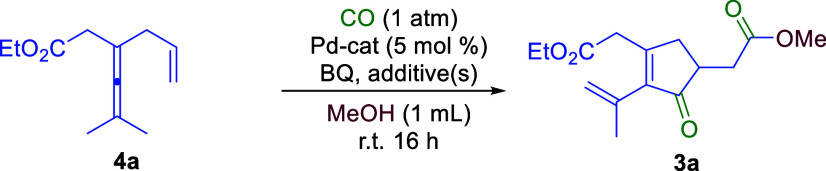
Optimization of the Reaction Conditions^a^

entry	additive(s) (20 mol %)	catalyst	yield of **3a** (%)^b^
1	none	Pd(OAc)_2_	0
2	DMSO	Pd(OAc)_2_	76
3	DMSO	Pd(TFA)_2_	43
4	DMSO	Pd_2_(dba)_3_	53
5^c^	DMSO	Pd(OAc)_2_	63
6^d^	DMSO	Pd(OAc)_2_	71
7^e^	DMSO	Pd(OAc)_2_	78
8^e^,^f^	AcOH (10 mol %) and MeOH (5 equiv)	Pd(OAc)_2_	12
9^e^	DMSO and AcOH (10 mol %)	Pd(OAc)_2_	82 (80)
10^e^,^g^	DMSO and chiral PA (10 mol %)	Pd(OAc)_2_	79 (7% ee)

a The reaction was conducted in MeOH (1 mL) with **4a** (0.2 mmol), BQ, and additive(s) in the presence of a Pd catalyst (5 mol %) at room temperature under 1 atm of CO (balloon) for 16 h.

b The yield was determined by ^1^H NMR analysis using anisole as the internal standard. The number in parentheses is the isolated yield.

c The reaction was conducted at 50 °C.

d The reaction was conducted at 40 °C.

e The reaction was conducted at 0 °C.

f Toluene was used as the solvent.

g For chiral PA, (*R*)-(−)-VAPOL hydrogen phosphate was used.

Subsequently, the reaction conditions were further optimized (Table 1, entries 3–9). Screening of Pd sources showed that Pd(OAc)_2_ is the superior catalyst (Table 1, entries 3 and 4). Reaction at 0 °C gave a chemoselectivity and a yield better than those of the reactions at room temperature or higher temperatures (Table 1, entries 5–7). When the reaction was carried out in toluene with 5 equiv of MeOH, the yield decreased to 12% (Table 1, entry 8). Using AcOH as an extra additive further increased the yield of **3a** to 82% with an 80% isolated yield (Table 1, entry 9). Attempts to develop an enantioselective version of this cascade reaction using chiral phosphates as counterions or ligands were unsuccessful; a poor enantioselectivity with only 7% ee of product **3a** was obtained (Table 1, entry 10). When ethanol was used as the solvent, there was no detectable ethyl ester product formed due to the slower acylpalladium alcoholysis compared to that of methanol.^13b,41,42^

Under the optimal reaction conditions, the scope of enallenes in the carbocyclization was investigated (Scheme 2). With a change in the two terminal methyl groups at the allene moiety to a cyclopentyl group (**4b**), the reaction worked smoothly giving the corresponding product (**3b**) in 71% yield. Substrates with different ring sizes were further examined. The reaction of the six-membered ring substrate (**4c**) gave a high yield (87%) of **3c**; however, the reactions with seven- and eight-membered ring substrates resulted in slightly lower yields (60% for **3d** and 66% for **3e**). We continued to investigate the functional group compatibility of this transformation. Functional groups such as sulfonyl ester (**4f**), ether (**4g**), imide (**4h**), and free hydroxyl group (**4i**) were compatible with the reaction conditions, giving good to high yields of **3f** (60%), **3g** (76%), **3h** (67%), and **3i** (92%), respectively. Interestingly, the introduction of a methyl substituent (R^4^) on the olefin moiety of the substrate did not affect the reactivity, and product **3j** was obtained in 81% yield. The reaction of dissymmetric substrate **4k** (R^1^ = H, and R^2^ = CHMe_2_) produced two products, **3ka** and **3kb** [another isomer (see the Supporting Information for details)], in 44% and 19% yields, respectively. Another dissymmetric substrate, **4l** (R^1^ = H, and R^2^ = Ph), worked well and afforded the corresponding cyclopentenone **3l** in 69% yield.

**Scheme 2:**
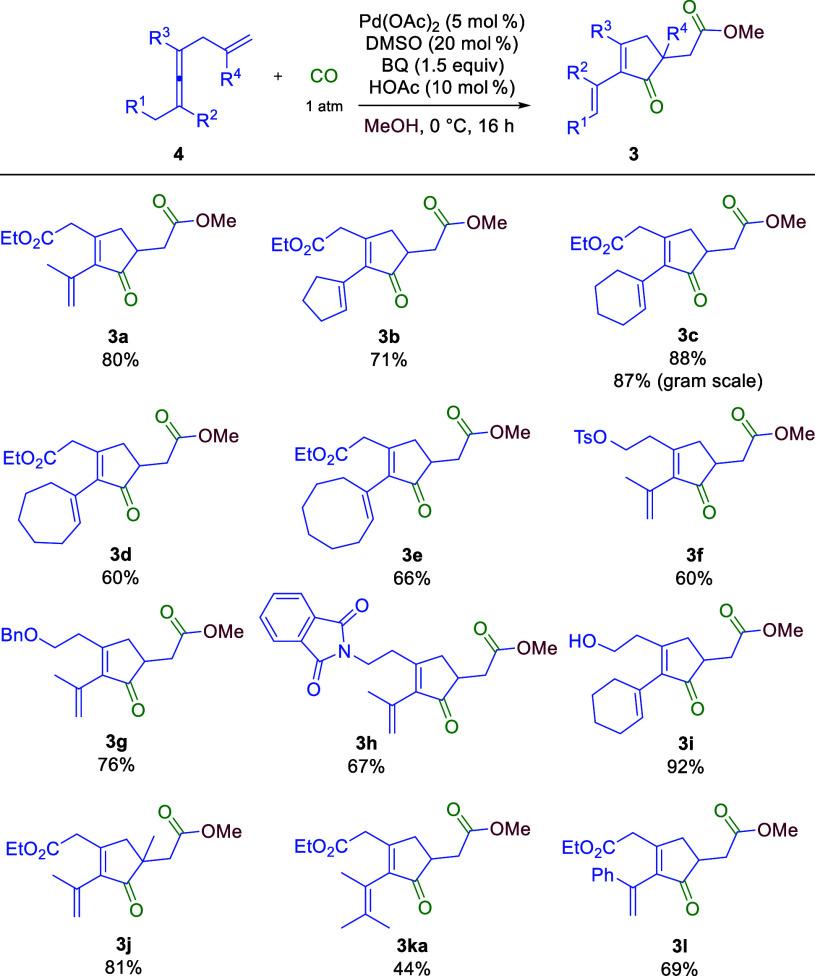
Scope of Enallenes for the Pd-Catalyzed Carbonylation/Carbocyclization/Carbonylation/Alkoxylation Cascade Reaction conditions: 0.2 mmol of **4**, 5 mol % Pd(OAc)_2_, 20 mol % DMSO, 10 mol % HOAc, and 1.5 equiv of BQ under 1 atm of CO (balloon) for 16 h.

On the basis of the cascade reactions depicted in Scheme 2, we developed a one-pot process involving Pd-catalyzed carbonylative carbocyclization and elimination of TsOH from the tosylated product in the presence of Et_3_N to afford cyclopentenone **3m** (in 63% yield) and **3n** (in 84% yield), which could serve as useful intermediates for the synthetic route to SLs (Scheme 3).

**Scheme 3:**
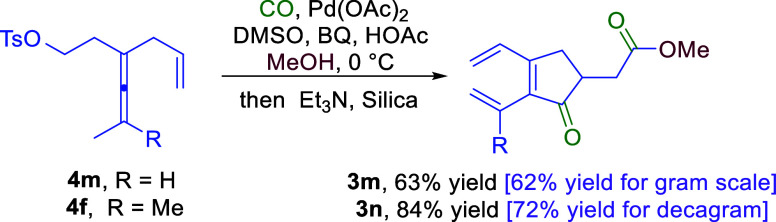
One-Pot Cyclization and Elimination to Obtain Trienes (**3m** and **3n**)

Kinetic isotope effect studies suggest that the formation of ***Int*-1** (Scheme 1B), involving C(sp^3^)–H be the rate-determining step (RDS) (cf. the Supporting Information for a detailed discussion). A proposed reaction mechanism of the Pd-catalyzed dehydrogenative carbonylation/carbocyclization/carbonylation/alkoxylation cascade was suggested (see the Supporting Information for a detailed discussion).

On the basis of the developed palladium-catalyzed oxidative carbonylative carbocyclization of enallenes, we then set out to study the synthesis of naturally occurring and artificial SLs (Scheme 4). The synthesis commenced with the preparation of readily available enallene **4f** from 2-methylhept-6-en-3-yn-2-ol via a three-step transformation involving a Johnson–Claisen rearrangement, reduction, and tosylation on a large scale [28.0 g (see the Supporting Information for a detailed description)]. Subsequently, a one-pot carbonylation/carbocyclization/carbonylation/alkoxylation cascade ending with a tosylate elimination was conducted on a decagram scale, providing **3n** in good yield (72%), which demonstrates the practical applicability of this synthetic strategy (Scheme 3). Afterward, an extensive screening of the reaction parameters for the electrocyclization of conjugated triene **3n** (for the cyclization reaction to give the A-ring moiety of SLs) indicated that using H_2_O and sodium acetate as additives was crucial to achieving a high yield of **5a** (88%) on a gram scale (Scheme 4A) (see the Supporting Information for a detailed discussion). To our delight, the conjugate addition of dimethyl cuprate to **5a** exclusively afforded the desired product **6a** in excellent yield (97%). With key intermediate **6a** at our disposal, we synthesized lactone **2a** through a one-pot process involving ester hydrolysis, Luche reduction, and lactone formation. Subsequently, *rac*-5-deoxystrigol (*rac*-**1a**) and its 2′-epimer (*rac*-**1b)** were synthesized as outlined in Scheme 4A. The latter transformation was achieved using a previously reported hydroxymethylenation/ether formation procedure, albeit with minor modifications.^23^ Importantly, the characterization data for both *rac*-**1a** and *rac*-**1b** matched those reported in the literature.^43^

**Scheme 4:**
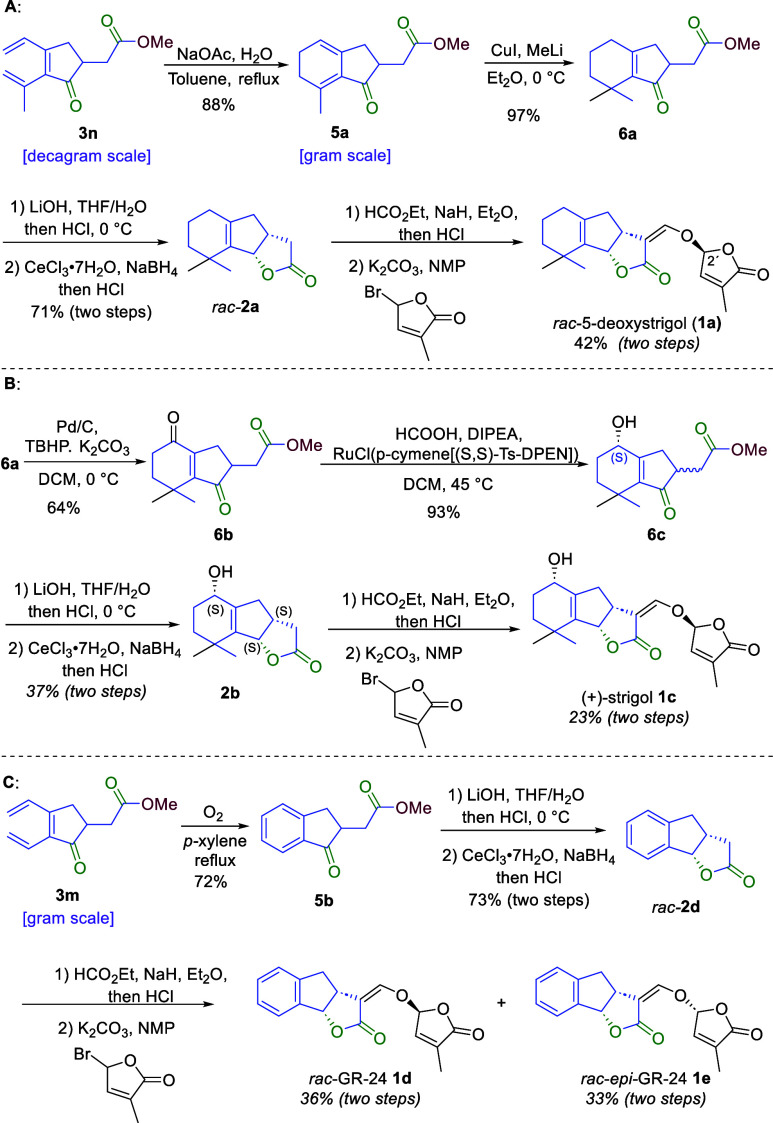
Total Synthesis of SLs and Their Derivatives: (A) Completion of the Synthesis of 5-Deoxyl-strigolactone, (B) Completion of the Asymmetric Total Synthesis of (+)-Strigol, and (C) Completion of the Four-Step Synthesis of GR-24

Then, we set out to complete the asymmetric synthesis of (+)-strigol **1c** (Scheme 4B). After an extensive screening of oxidation parameters, we found that the allylic position at the A-ring moiety was exclusively oxidized into a ketone using TBHP (10 equiv) as the oxidant, Pd/C (10 mol %) as the catalyst, and K_2_CO_3_ (1.0 equiv) as the additive in DCM at 0 °C. Compound **6b** was obtained in good yield (64%), with the allylic position on the B ring remaining untouched under the optimal reaction conditions. An asymmetric transfer hydrogenation of the keto group of **6b** was achieved using DIPEA (2.3 equiv), formic acid (6.0 equiv), and RuCl(*p*-cymene)[(*S,S*)-Ts-DPEN] (2 mol %) in DCM at 45 °C, affording product **6c** in excellent yield (93%) with the exclusively *S* configuration of the alcohol, but as a mixture of two diastereomers. Subsequently, the C-ring moiety was constructed via a one-pot process involving ester hydrolysis, Luche reduction, and lactone formation, resulting in **2b** and its diastereomer, **2c**, which could be separated from each other, being obtained in 37% and 35% yields, respectively. The synthesis of (+)-strigol **1c** from **2b** was carried out using a reported hydroxymethylenlation/ether formation process in a one-pot procedure.^23^ (+)-Strigol **1c** was obtained in 23% overall yield for the two steps, and its characterization data were in good agreement with those reported in the literature.^23^

We then proceeded by synthesizing GR-24 (as depicted in Scheme 4C). This involved a gram-scale synthesis of **3m**, in 62% yield from substrate **4m** (Scheme 3). In our efforts to optimize the oxidative cyclization of cyclopentenone **3m**, which is a key step in the construction of the A ring of GR-24, we identified *p*-xylene as the most effective solvent. This solvent facilitated the desired aromatization at an increased temperature under an O_2_ atmosphere, affording **5b** in 72% yield. Subsequently, the C-ring moiety was obtained through a one-pot procedure encompassing ester hydrolysis, NaBH_4_ reduction, and lactone formation, giving **2d** in 73% yield. Armed with these findings, we successfully synthesized both *rac*-GR-24 (*rac*-**1d**) and *rac*-*epi*-GR-24 (*rac*-**1e**), adhering to a previously reported procedure but with minor modifications.^24^

In conclusion, we have developed a scalable palladium-catalyzed carbonylation/carbocyclization/carbonylation/alkoxylation cascade (in which three C–C bonds and one C–O bond were formed in a single step) and identified efficient total synthetic pathways for SLs and their derivatives. Ongoing research is currently exploring the variations of this synthetic strategy to provide further insight and advancement in the field.

## Data Availability

The data underlying this study are available in the published article and its Supporting Information.

## Supporting Information Available

General experimental procedures, tables of reaction optimization, mechanistic study, and characterization data, including ^1^H and ^13^C NMR spectra, for all new compounds (PDF)
