# Supplementary material for: Total Synthesis of Strigolactones via Palladium-Catalyzed Cascade Carbonylative Carbocyclization of Enallenes
Source: Org Lett. 2024 May 28;26(22):4637–42. doi: 10.1021/acs.orglett.4c01283 (PMC11165582; doi:10.1021/acs.orglett.4c01283)
Supplement: Supplementary file 1 — ol4c01283_si_001.pdf [file ol4c01283_si_001.pdf]

Supporting Information

Total Synthesis of Strigolactones via Palladium-Catalyzed Cascade Carbonylative Carbocyclization of Enallenes

Bin Yang,[a,b] *Patrick Federmann,*[a] *Viktoria Warth,*[a] *Mingzhe Ren,*[b] *Xin Mu,**[b] *Haibo Wu,**[a] *Jan-E. Bäckvall**[a]

[a]*Department of Organic Chemistry, Arrhenius Laboratory, Stockholm University, SE-106 91 Stockholm, Sweden.*

[b]*School of Chemistry, Xi'an Jiaotong University, 710049, Xi'an, P. R. China.*

Corresponding authors: Xin Mu − Email: ximu1331@xjtu.edu.cn

Haibo Wu − Email: haibo.wu@su.se

Jan-E. Bäckvall − Email: jeb@organ.su.se

Table of Contents

Table of Contents S1

General Remarks S2

1. Preparation of Starting Materials S3

2. Formation of Highly Substituted Cyclopentenone **3** from Enallene **4** and Construction of B-ring Moiety of SLs S6

   2.1 Reaction Conditions Optimization for the Carbonylative Cascade S6

   2.2 General Procedure for the Synthesis of **3** S9

   2.3 Gram-Scale Synthesis for the Synthesis of **3c**. S16

   2.4 General Procedure for the Small-scale Sequential Synthesis of Vinyl-Cyclopentenone **2** from TsO-functionalized Enallenes. S17

   2.5 Gram-Scale Synthesis of **3m** S18

   2.6 Decagram-Scale Synthesis of **3n** S19

   2.7 Kinetic Isotope Effect (KIE) Experiments and Proposed Mechanism S20

   2.8 Proposed Mechanism S23

3. The Construction of A-ring Moiety of SL S25

   3.1 Percyclization Reaction for A-ring Construction S25

   3.2 Functionalization of the A-ring moieties S28

   3.3 Oxidative Percyclization reaction for A-ring Construction of GR-24 .... S30

4. Construction of the C-Ring and D-Ring Moieties of Strigols S31

5. Copies of Spectra S37

6. References S67

General Remarks

All reagents were used directly from commercial suppliers, unless specified otherwise. The palladium-catalyzed cascade reactions were conducted without special precautions to exclude moisture. The progress of the reactions was monitored via thin-layer chromatography (TLC) on SiO_2 plates, which were visualized under UV light at 254 nm or by staining with $KMnO_4$. Flash chromatography was performed using 60Å silica gel with a particle size of 35-70 µm. NMR spectra were acquired at 400 MHz for ^{1}H and 100 MHz for ^{13}C. Chemical shifts (δ) are expressed in ppm, referencing the residual solvent peaks in $CDCl_3$ (^{1}H = 7.26 ppm, ^{13}C = 77.0 ppm). Coupling constants (*J*) are reported in Hertz (Hz). Multiplicity is denoted as: s (singlet), d (doublet), t (triplet), q (quartet), dd (doublet of doublets), app-d (appear as doublet), and m (multiplet). High-resolution mass spectra (HRMS) were obtained using electrospray ionization time-of-flight (ESI-TOF) methods. All experiments were performed at room temperature (23 °C), unless indicated otherwise.

1. *Preparation of Starting Materials*

Enallene **4a**, **4g**, **4f**, **4i** were prepared as previously described.[1] Enallene **4b**, **4c** and **4k** were prepared based on a literature procedure.[2] Enallene **4d** and **4e** were synthesized based on previous report.[3] Enallene **4h** and **4j** were prepared based on a literature procedure.[4]

Ethyl 3-allyl-5-phenylhexa-3,4-dienoate ***(4l)***

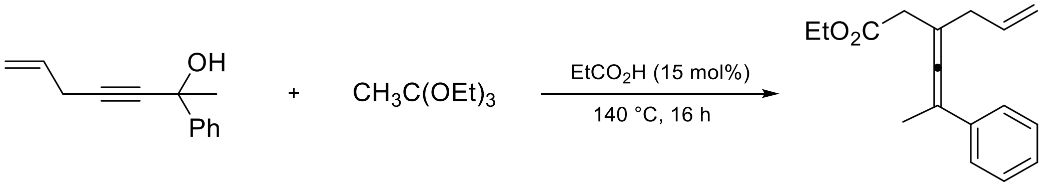

A1 **4l**, 62%

A1 was prepared based on a literature procedure.[5] In a dry round-bottomed flask containing propargylic alcohol **A1** (4.00 g, 21.5 mmol), equipped with a distillation receiver and a condenser, triethyl orthoacetate (50 mL) and propanoic acid (0.241 mL, density = 0.99 g/mL, 5.45 mmol) were added sequentially. The reaction mixture was then refluxed at 140 °C for 4 hours. After the reaction, the mixture was cooled to 0 °C using an ice bath. HCl (1 M, 30 mL) was added, leading to the separation of the layers. The aqueous layer was extracted with ethyl ether (2 × 50 mL). The combined organic extracts were dried over Na_2SO_4, filtered, and concentrated under reduced pressure. The residue was purified using column chromatography on silica gel with eluent: PE / EA = 40:1, yielding the desired product **4l** as a colorless oil (3.42 g, 62% yield). ^{1}H NMR (400 MHz, $CDCl_3$) δ 7.47 – 7.39 (m, 2H), 7.35 – 7.27 (m, 2H), 7.22 – 7.17 (m, 1H), 5.89 – 5.78 (m, 1H), 5.16 – 5.03 (m, 2H), 4.16 – 4.08 (m, 2H), 3.09 (s, 2H), 2.95 – 2.89 (m, 2H), 2.09 (s, 3H), 1.25 – 1.19 (m, 3H); ^{13}C NMR (101 MHz, $CDCl_3$) δ 203.2, 171.2, 137.3, 135.2, 128.2, 126.6, 125.9, 125.8, 116.6, 101.8, 98.5, 60.7, 38.5, 37.5, 17.0, 14.1. HRMS (ESI): calc. for $C_{17}H_{20}NaO_2^+$ $[M+Na]^+$: 279.1356, found 279.1366.

3-Allylhexa-3,4-dien-1-yl 4-methylbenzenesulfonate ***(4m)***

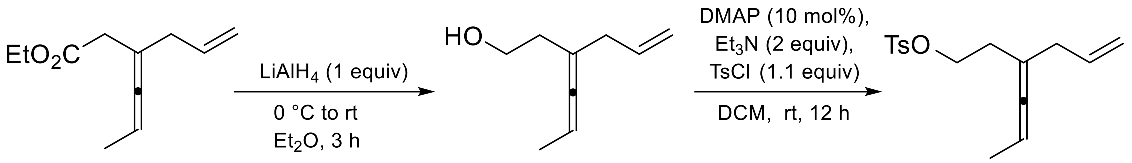

A2 **A3**, 85% **4m** 75%

A2 was prepared based on a literature procedure.[6] A solution of 3,4-dienoate **A2** (3.60 g, 20.0 mmol) in anhydrous diethyl ether (Et_2O, 5 mL) was gradually added to a stirred suspension of lithium aluminum hydride ($LiAlH_4$, 0.76 g, 20.0 mmol) in anhydrous Et_2O (200 mL, 10 mL/mmol) at 0 °C under a nitrogen atmosphere. The mixture was stirred at room temperature for 3 hours, then cautiously quenched with water (2 mL). The organic phase was separated, and the aqueous phase was extracted with dichloromethane (DCM, 2 × 200 mL). The combined organic extracts were dried over sodium sulfate (Na_2SO_4), filtered, and concentrated under reduced pressure. The residue was purified by column chromatography on silica gel (eluent: petroleum ether/ethyl ether = 15:1), yielding **A3** (2.35 g, 85%) as a colorless oil. In a separate process, 3,4-dienol **A3** (622.0 mg, 4.50 mmol) was placed in a single-neck flask, followed by the addition of anhydrous DCM (20 mL), 4-dimethylaminopyridine (DMAP, 55 mg, 0.45 mmol), triethylamine (Et_3N, 1.25 mL, density = 0.73 g/mL, 9.0 mmol), and tosyl chloride (TsCl, 953.0 mg, 5 mmol) in sequence. The mixture was stirred at room temperature for 24 hours. The reaction was then quenched with hydrochloric acid (1 M, 20 mL) at 0 °C. The organic layer was separated, and the aqueous layer was extracted with DCM (2 × 20 mL). The combined organic layers were dried over Na_2SO_4, filtered, and concentrated. The product was purified using silica gel column chromatography [eluent: petroleum ether (PE)/ethyl acetate (EA) 30:1 to 10:1] to obtain the desired **4m** (colorless oil, 986.8 mg, 75% yield). ^1H NMR (400 MHz, $CDCl_3$) δ 7.82 – 7.76 (2H, m), 7.37 – 7.31 (m, 2H), 5.81 – 5.57 (m, 1H), 5.12 – 4.94 (m, 3H), 4.15 – 4.05 (m, 2H), 2.68 – 2.59 (m, 2H), 2.45 (s, 3H), 2.32 – 2.21 (m, 2H), 1.57 (d, J = 6.9 Hz, 3H); ^{13}C NMR (101 MHz, $CDCl_3$) δ 201.9, 144.6, 135.5, 133.2, 129.8, 127.9, 116.1, 97.0, 88.0, 68.7, 37.7, 31.1, 21.6, 14.6. HRMS (ESI): calc. for $C_{16}H_{21}O_3S^+$ $[M+H]^+$: 293.1206; found: 293.1201.

Large scale synthesis of ***4f***

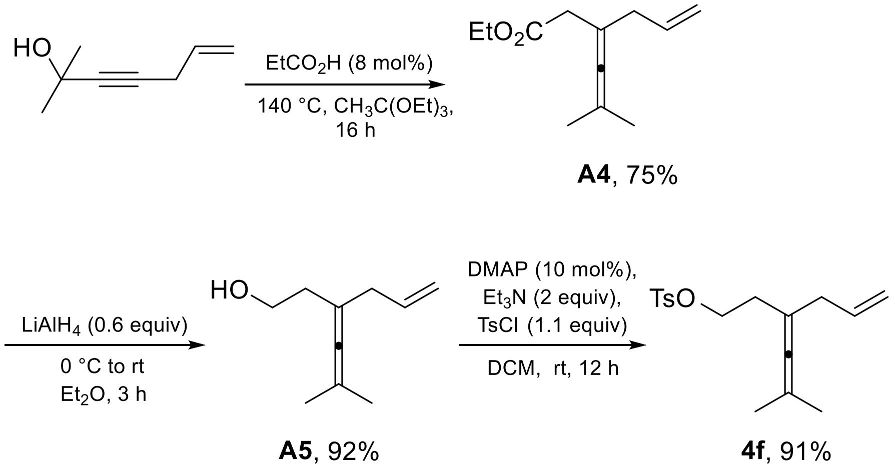

A round-bottomed flask was set up with a distillation receiver and a condenser for the reaction. Initially, 2-methylhept-6-en-3-yn-2-ol (24.84 g, 200 mmol) was added, followed by triethyl orthoacetate (300 mL), and then propanoic acid (1.18 g, 16 mmol). The mixture was refluxed for 16 hours and subsequently cooled to 0 °C using an ice bath. Et_2O (500 mL) and HCl (1 M, 200 mL) were then added. The organic layer was separated from the aqueous layer, which was further extracted twice with Et_2O (200 mL each). The combined organic extracts were dried over Na_2SO_4, filtered, and evaporated. Purification via column chromatography on silica gel (eluent: PE/diethyl ether = 20/1) yielded the product **A4** (29.335 g, 151 mmol, 75%).

A solution of 3,4-dienoate **A4** (29.335 g, 151 mmol) in dry Et_2O (50 mL) was gradually added to a stirred suspension of $LiAlH_4$ (3.42 g, 91 mmol) in dry Et_2O (1000 mL) at -78 °C under a nitrogen atmosphere. The mixture was stirred at 0 °C for 2 hours, then cautiously quenched with water (10 mL) at the same temperature. After separating the organic layer, the aqueous phase was extracted twice with Et_2O (300 mL each). The combined organic layers were dried over Na_2SO_4, filtered, and evaporated. The residue was dissolved in Et_2O (200 mL) and quickly passed through a short column of silica gel (2 cm, eluent: 200 mL of Et_2O). Direct evaporation of the solvent yielded pure product **A5** (21.1480 g, 138.9 mmol, 92%) as a colorless oil.

In a separate procedure, 3,4-dienol **A5** (15.22 g, 10.0 mmol) was added to a single-neck flask, followed by DCM (200 mL), 4-dimethylaminopyridine (DMAP, 122 mg, 1.0 mmol), Et_3N (27.7 mL, density = 0.73 g/mL, 200 mmol), and TsCl (20.97 g, 11.0 mmol). The mixture was stirred at room temperature for 24 hours. The reaction was quenched by adding HCl (1 M, 200 mL) at 0 °C.

The organic layer was separated, and the aqueous layer was further extracted twice with DCM (200 mL each). The combined organic extracts were dried over Na_2SO_4, filtered, and evaporated. Purification via column chromatography on silica gel (eluent: PE/EA 20/1 to 10/1) yielded the desired product **4f** (28.0 g, 91.3 mmol, 91%). The spectral data are in good agreement with literature values.[1]

2. *Formation of Highly Substituted Cyclopentenone 3 from Enallene 4 and Construction of B-ring Moiety of SLs*

2.1 Reaction Conditions Optimization for the Carbonylative Cascade

Table S1 Catalyst and ligand screening[a]

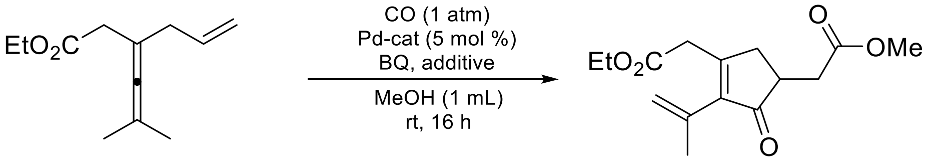

4a 3a

Entry	Catalyst (10 mol%)	Ligand (20 mol%)	Yield of **3a** (%)[b]	Recovery of **4a** (%)[b]
1	$Pd(OAc)_2$	none	n.d.	0
**2**	**$Pd(OAc)_2$**	**DMSO**	**76**	**0**
3	$Pd(TFA)_2$	DMSO	43	0
4	$Pd_2(dba)_3$	DMSO	53	0
5	$Pd(PPh_3)_4$	DMSO	22	53
6	$PdCl_2$	DMSO	n.d.	72
7	$Pd(CH_3CN)_2Cl_2$	DMSO	n.d.	21
8	$Pd(PPh_3)_2(OAc)_2$	DMSO	69	0

[a] The reaction was carried out in MeOH (0.5 mL) with substrate **4a** (0.1 mmol), 1,4-benzoquinone (BQ, 1.5 equiv), and additive (ligand), in the presence of a palladium catalyst (5 mol%). This was conducted at room temperature under a CO atmosphere (1 atm, provided by a balloon) for 16 hours. [b] The yield was determined by ^{1}H-NMR analysis, using anisole or nitromethane ($MeNO_2$) as the internal standard.

Table S2 Effects of oxidant and its amount[a]

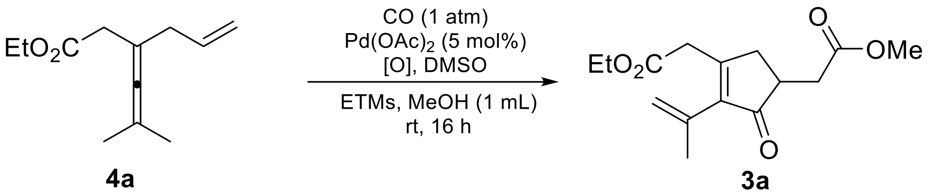

Entry	Oxidant [O]	ETMs	Yield of **3a** (%)[b]	Recovery of **4a** (%)[b]
**1**	**BQ (1.5 equiv)**	**-**	**76**	**0**
2[c]	O_2 (~0.5 atm)	BQ (20 mol%) + Co(salophen) (10 mol%)	14	<1
3	2,6-dimethyl-*p*-benzoquinone (1.5 equiv)	-	69	0
4	2,5-dimethyl-*p*-benzoquinone (1.5 equiv)	-	72	0
5	BQ (1.3 equiv)	-	73	0
6	BQ (1.7 equiv)	-	76	0

[a] The reaction was carried out in MeOH (0.5 mL) with compound **4a** (0.1 mmol), an oxidant (1.5 equiv), and DMSO (20 mol%) in the presence of $Pd(OAc)_2$ (5 mol%). This process was conducted at the specified temperature under a carbon monoxide atmosphere (1 atm, supplied by a balloon) for 16 hours. [b] The yield was determined using ^{1}H-NMR analysis with anisole or $MeNO_2$ as the internal standard. [c] The reaction was performed in MeOH (0.5 mL) with compound **4a** (0.1 mmol), BQ (20 mol%), Co(salophen) (10 mol%), and DMSO (20 mol%) in the presence of $Pd(OAc)_2$ (5 mol%). This was carried out at room temperature under a mixed atmosphere (from a balloon) of CO (approximately 0.5 atm) and O_2 (approximately 0.5 atm) for 16 hours.

Table S3 Effect of temperature, catalyst loading, additive and reaction time[a]

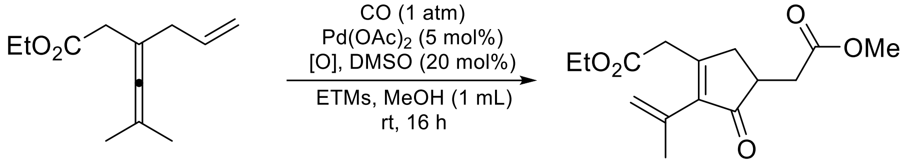

4a **3a**

Entry	Additive(s)	Temperature	Yield of **3a** (%)*[b]*	Recovery of **4a** (%)[b]
1	-	50 °C	63	0
2	-	40 °C	71	<1
3	-	0 °C	78	0
4	-	rt	76	0
**5**	**AcOH (10 mol%)**	**0 °C**	**82 (80)**	**0**
6	AcOH (50 mol%)	0 °C	80	0
7[c]	AcOH (10 mol%) + MeOH (5 equiv)	0 °C	12	13
8	chiral *acid[d]* (10 mol%)	0 °C	79 [7% *ee*]	0
9[e]	AcOH (10 mol%)	0 °C	81	<1

[a] The reaction was performed in MeOH (0.5 mL) with compound **4a** (0.1 mmol), BQ (1.5 equiv), and additive(s), in the presence of $Pd(OAc)_2$ (5 mol%). This process was conducted at the specified temperature under an atmosphere of CO (1 atm, supplied by a balloon) for 16 hours. [b] The yield was determined using ^{1}H-NMR analysis with anisole as the internal standard. The numbers in parentheses indicate the isolated yield, while the numbers in square brackets represent the enantiomeric excess, as determined by chiral HPLC. [c] The reaction was carried out in toluene instead of MeOH. [d] The additive used was (*R*)-(-)-VAPOL hydrogenphosphate (10 mol%).[3] [e] The reaction time was changed to 8 hours.

2.2 General Procedure for the Synthesis of 3

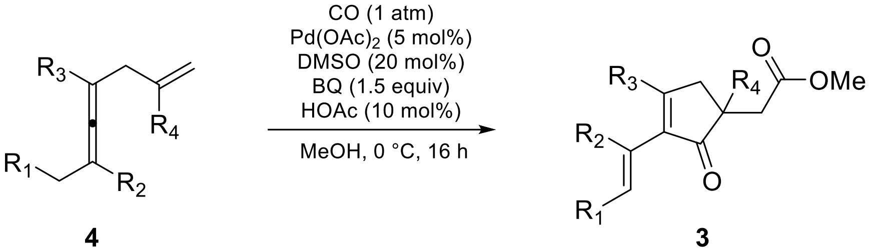

These reactions were conducted without special precautions to exclude moisture. In a vial with a magnetic stirring bar, $Pd(OAc)_2$ (2.2 mg, 0.01 mmol, 5 mol%) and BQ (32.4 mg, 0.30 mmol, 1.5 equiv) were dissolved in 0.5 ml MeOH. Enallene **4** (0.20 mmol), DMSO (, 2.9 µl, 0.04 mmol, 20 mol%), acetic acid (1.2 µl, 0.02 mmol, 10 mol%), and an additional 0.5 ml of MeOH were then added in sequence. The vial was sealed with a septum and subjected to three cycles of evacuation and filling with CO gas using a balloon. The reaction was stirred at 0 °C for 16 hours. Upon complete consumption of the starting enallene **4**, as confirmed by TLC, the reaction mixture was concentrated under reduced pressure and the product was purified by silica gel column chromatography.

*Ethyl 2-(4-(2-methoxy-2-oxoethyl)-3-oxo-2-(prop-1-en-2-yl)cyclopent-1-en-1-yl) acetate (**3a**)*

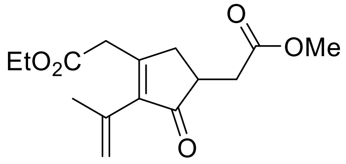

3a

Eluent: PE/EA = 5/1 (+0.1% triethylamine). Following the general procedure (0.20 mmol scale), **2a** (44.9 mg, 0.160 mmol, 80% yield) was obtained as a slightly yellow oil. ^{1}H NMR (400 MHz, $CDCl_3$) δ 5.23 – 5.20 (m, 1H), 4.86 – 4.84 (m, 1H), 4.17 (q, J = 7.1 Hz, 2H), 3.68 (s, 3H), 3.50 (s, 2H), 2.96 (dd, J = 18.5 Hz, 7.0 Hz, 1H), 2.86 (dd, J = 16.4 Hz, 4.1 Hz, 1H), 2.82 – 2.76 (m, 1H), 2.49 – 2.39 (m, 2H), 1.93-1.91 (m, 3H), 1.27 (t, J = 7.1 Hz, 3H); ^{13}C NMR (101 MHz, $CDCl_3$) δ 207.3, 172.4, 169.1, 162.8, 144.4, 136.9, 117.3, 61.3, 51.8, 41.7, 37.2, 36.9, 35.0, 21.8, 14.1. HRMS (ESI): calc. for $C_{15}H_{20}NaO_5^+$ $[M+Na]^+$: 303.1203, found: 303.1203.

*Ethyl 2-(4-(2-methoxy-2-oxoethyl)-5-oxo-[1,1'-bi(cyclopentane)]-1,1'-dien-2-yl) acetate (**3b**)*

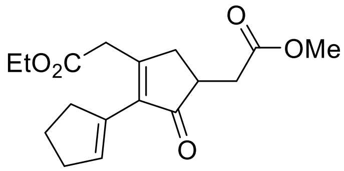

3b

Eluent: PE/EA = 5/1 (+0.1% triethylamine). Following the general procedure (0.20 mmol scale), **3b** (43.3 mg, 0.142 mmol, 71% yield) was obtained as a slightly yellow oil. ^{1}H NMR (400 MHz, $CDCl_3$) δ 6.12 – 6.08 (m, 1H), 4.18 (q, *J* = 7.2 Hz, 2H), 3.68 (s, 3H), 3.54 (s, 2H), 2.96 (dd, *J* = 18.6 Hz, 7.1 Hz, 1H), 2.86 (dd, *J* = 16.3 Hz, 4.1 Hz), 2.83 – 2.77 (m, 1H), 2.63 – 2.57 (m, 2H), 2.47 – 2.39 (m, 4H), 1.90 (q, *J* = 7.5 Hz, 2H), 1.28 (t, *J* = 7.1 Hz, 3H); ^{13}C NMR (101 MHz, $CDCl_3$) δ 207.7, 172.5, 169.2, 161.9, 138.4, 134.1, 133.3, 61.3, 51.8, 41.8, 37.7, 37.3, 35.1, 34.5, 33.0, 23.3, 14.1. HRMS (ESI): calc. for $C_{17}H_{22}NaO_5^+$ $[M+Na]^+$: 329.1359, found: 329.1367.

*Ethyl 2-(2-(cyclohex-1-en-1-yl)-4-(2-methoxy-2-oxoethyl)-3-oxocyclopent-1-en-1-yl) acetate (**3c**)*

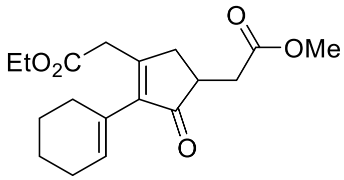

3c

Eluent: PE/EA = 5/1 (+0.1% triethylamine). Following the general procedure (0.20 mmol scale), **3c** (56.4 mg, 0.176 mmol, 88% yield) was obtained as a slightly yellow oil. ^{1}H NMR (400 MHz, $CDCl_3$) δ 5.59 – 5.56 (m, 1H), 4.17 (q, *J* = 7.2 Hz, 2H), 3.68 (s, 3H), 3.47 (s, 2H), 2.93 (dd, *J* = 18.3 Hz, 7.0 Hz, 1H), 2.85 (dd, *J* = 16.4 Hz, 4.2 Hz, 1H), 2.80 – 2.73 (m, 1H), 2.46 – 2.35 (m, 2H), 2.15 – 2.07 (m, 4H), 1.70 – 1.60 (m, 4H), 1.27 (t, *J* = 7.2 Hz, 3H); ^{13}C NMR (101 MHz, $CDCl_3$) δ 207.9, 172.5, 169.3, 162.3, 144.8, 130.1, 128.9, 61.2, 51.8, 41.7, 37.3, 36.8, 35.1, 27.3, 25.3, 22.4, 21.9, 14.2. HRMS (ESI): calc. for $C_{18}H_{24}NaO_5^+$ $[M+Na]^+$: 343.1516, found: 343.1521.

*Ethyl 2-(2-(cyclohept-1-en-1-yl)-4-(2-methoxy-2-oxoethyl)-3-oxocyclopent-1-en-1-yl) acetate (**3d**)*

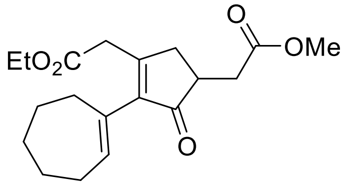

3d

Eluent: PE/EA = 5/1 (+0.1% triethylamine). Following the general procedure (0.20 mmol scale), **3d** (40.2 mg, 0.120 mmol, 60% yield) was obtained as a colorless slightly yellow oil. ^{1}H NMR (400 MHz, $CDCl_3$) δ 5.73 (t, *J* = 6.5 Hz, 1H), 4.17 (q, *J* = 7.0 Hz, 2H), 3.67 (s, 3H), 3.47 (s, 2H), 2.93 (dd, *J* = 18.3 Hz, 7.0 Hz, 1H), 2.84 (dd, *J* = 16.4 Hz, 4.2 Hz, 1H), 2.78 – 2.71 (m, 1H), 2.46 – 2.35 (m, 2H), 2.26 – 2.19 (m, 4H), 1.81 – 1.74 (m, 2H), 1.61 – 1.51 (m, 4H), 1.27 (t, *J* = 7.1 Hz, 3H); ^{13}C NMR (101 MHz, $CDCl_3$) δ 208.0, 172.5, 169.4, 161.0, 146.4, 136.3, 134.6, 61.2, 51.8, 41.6, 37.3, 36.7, 35.1, 32.5, 32.2, 28.9, 26.9, 26.6, 14.1. HRMS (ESI): calc. for $C_{19}H_{26}NaO_5^+$ $[M+Na]^+$: 357.1672, found: 357.1674.

*Ethyl 2-(2-(cyclooct-1-en-1-yl)-4-(2-methoxy-2-oxoethyl)-3-oxocyclopent-1-en-1-yl) acetate (**3e**)*

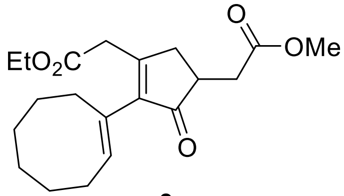

3e

Eluent: PE/EA = 5/1 (+0.1% triethylamine). Following the general procedure (0.20 mmol scale), **3e** (45.9 mg, 0.132 mmol, 66% yield) was obtained as a slightly yellow oil. ^{1}H NMR (400 MHz, $CDCl_3$) δ 5.51 (t, *J* = 8.2 Hz, 1H), 4.17 (q, *J* = 7.1 Hz, 2H), 3.67 (s, 3H), 3.50 (s, 2H), 2.94 (dd, *J* = 18.3 Hz, 7.0 Hz, 1H), 2.84 (dd, *J* = 16.4 Hz, 4.2 Hz, 1H), 2.80 – 2.73 (m, 1H), 2.47 – 2.31 (m, 4H), 2.26 – 2.18 (m, 2H), 1.59 – 1.43 (m, 8H), 1.26 (t, *J* = 7.1 Hz, 3H); ^{13}C NMR (101 MHz, $CDCl_3$) δ 208.1, 172.4, 169.3, 162.6, 145.2, 133.2, 132.2, 61.2, 51.8, 41.7, 37.2, 36.7, 35.1, 29.5, 28.4, 28.1, 26.5, 26.4, 26.3, 14.1. HRMS (ESI): calc. for $C_{20}H_{28}NaO_5^+$ $[M+Na]^+$: 371.1829, found: 371.1830.

*methyl 2-(2-oxo-3-(prop-1-en-2-yl)-4-(2-(tosyloxy)ethyl)cyclopent-3-en-1-yl)acetate (**3f**)*

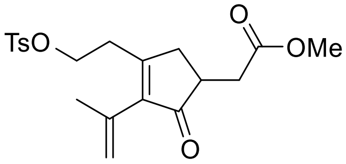

3f

Eluent: PE/EA = 2/1. Following the general procedure (0.20 mmol scale), **3f** (47.2 mg, 0.120 mmol, 60% yield) was obtained as a slightly yellow oil. Please note that **3f** is not very stable at room temperature. It is also interesting to note that when triethylamine was added to the eluent during the purification, **3f** was converted into **3n** in quantitative yield. ^{1}H NMR (400 MHz, $CDCl_3$) δ 7.79 – 7.74 (m, 2H), 7.38 – 7.33 (m, 2H), 5.16 – 5.14 (m, 1H), 4.75 – 4.73 (m, 1H), 4.20 (t, *J* = 6.3 Hz, 2H), 3.68 (s, 3H), 2.85 – 2.74 (m, 4H), 2.71 – 2.64 (m, 1H), 2.45 (s, 3H), 2.39 (dd, *J* = 16.7 Hz, 9.2 Hz, 1H), 2.27 – 2.20 (m, 1H), 1.86 – 1.84 (m, 3H); ^{13}C NMR (101 MHz, $CDCl_3$) δ 207.1, 172.3, 165.6, 145.2, 144.5, 136.8, 132.7, 129.9, 127.9, 117.1, 67.0, 51.8, 41.5, 36.2, 34.9, 30.8, 22.0, 21.6. HRMS (ESI): $C_{20}H_{24}NaO_6S^+$ $[M+Na]^+$: 415.1186, found: 415.1185.

*Methyl 2-(4-(2-(benzyloxy)ethyl)-2-oxo-3-(prop-1-en-2-yl)cyclopent-3-en-1-yl)acetate (**3g**)*

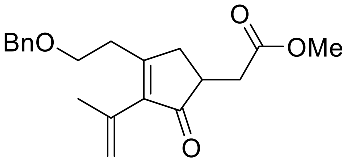

3g

Eluent: PE/EA = 5/1 (+0.1% triethylamine). Following the general procedure (0.20 mmol scale), **3g** (50.0 mg, 0.152 mmol, 76% yield) was obtained as a slightly yellow oil. ^{1}H NMR (400 MHz, $CDCl_3$) δ 7.38 – 7.26 (m, 5H), 5.20 – 5.17 (m, 1H), 4.84 – 4.82 (m, 1H), 4.50 (s, 2H), 3.67 (s, 3H), 3.65 (t, *J* = 6.5 Hz, 2H), 2.93 – 2.83 (m, 2H), 2.79 (t, *J* = 6.5 Hz, 2H), 2.77 – 2.70 (m, 1H), 2.38 (dd, *J* = 16.6 Hz, 9.5 Hz, 1H), 2.32 (dd, *J* = 18.4 Hz, 3.0 Hz, 1H), 1.92-1.89 (m, 3H); ^{13}C NMR (101 MHz, $CDCl_3$) δ 207.6, 172.6, 169.8, 143.2, 137.9, 137.2, 128.4, 127.7, 127.6, 116.9, 73.0, 67.5, 51.8, 41.6, 36.6, 35.2, 31.9, 22.2. HRMS (ESI): calc. for $C_{20}H_{24}NaO_4$ $[M+Na]^+$: 351.1567, found: 351.1566.

*Methyl 2-(4-(2-(1,3-dioxoisoindolin-2-yl)ethyl)-2-oxo-3-(prop-1-en-2-yl)cyclopent-3-en-1-yl)acetate (**3h**)*

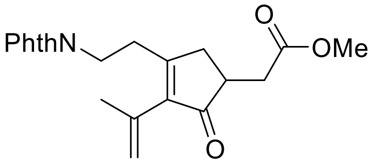

3h

Eluent: PE/EA = 5/1 (+0.1% triethylamine). Following the general procedure (0.20 mmol scale), **3h** (48.9 mg, 0.134 mmol, 67% yield) was obtained as a slightly yellow oil. ^{1}H NMR (400 MHz, $CDCl_3$) δ 7.86 – 7.80 (m, 2H), 7.75 – 7.69 (m, 2H), 5.08 – 5.05 (m, 1H), 4.63 – 4.60 (m, 1H), 3.93 (t, J = 6.9 Hz, 2H), 3.70 (s, 3H), 3.03 (dd, J = 18.3 Hz, 6.9 Hz, 1H), 2.92 – 2.81 (m, 3H), 2.81 – 2.74 (m, 1H), 2.53 – 2.43 (m, 2H), 1.75 – 1.72 (m, 3H); ^{13}C NMR (101 MHz, $CDCl_3$) δ 207.5, 172.5, 168.0, 167.7, 144.1, 136.4, 134.1, 131.8, 123.3, 117.1, 51.8, 41.7, 36.2, 35.6, 35.0, 30.5, 22.0. HRMS (ESI): calc. for $C_{21}H_{21}NNaO_5^+$ $[M+Na]^+$: 390.1312, found: 390.1313.

*methyl 2-(3-(cyclohex-1-en-1-yl)-4-(2-hydroxyethyl)-2-oxocyclopent-3-en-1-yl)acetate (**3i**)*

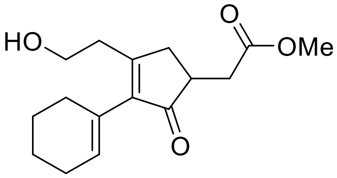

3i

Eluent: PE/EA = 5/1 (+0.1% triethylamine). Following the general procedure (0.20 mmol scale), **3i** (51.2 mg, 0.184 mmol, 92% yield) was obtained as a slightly yellow oil. ^{1}H NMR (400 MHz, $CDCl_3$) δ 5.56 – 5.52 (m, 1H), 3.82 (t, J = 6.3 Hz, 2H), 3.64 (s, 3H), 2.91–2.72 (m, 3H), 2.72 – 2.61 (m, 2H), 2.45 (dd, J = 16.5 Hz, 8.8 Hz, 1H), 2.36 (dd, J = 18.4 Hz, 2.8 Hz, 1H), 2.13 – 2.04 (m, 4H), 1.70 – 1.56 (m, 4H); ^{13}C NMR (101 MHz, $CDCl_3$) δ 208.3, 172.6, 169.0, 144.2, 130.2, 128.2, 60.4, 51.7, 41.3, 36.3, 35.0, 34.6, 27.7, 25.2, 22.5, 21.9. HRMS (ESI): calc. for $C_{16}H_{22}NaO_4^+$ $[M+Na]^+$: 301.1410, found: 301.1404.

Ethyl 2-(4-(2-methoxy-2-oxoethyl)-4-methyl-3-oxo-2-(prop-1-en-2-yl)cyclopent-1-en-1-yl)acetate ***(3j)***

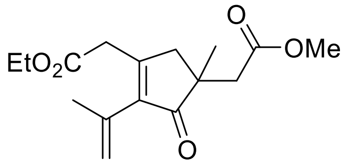

3j

Eluent: PE/EA = 5/1 (+0.1% triethylamine). Following the general procedure (0.20 mmol scale), **3j** (47.6 mg, 0.162 mmol, 81% yield) was obtained as a slightly yellow oil. ^{1}H NMR (400 MHz, $CDCl_3$) δ 5.21 – 5.18 (m, 1H), 4.86 – 4.84 (m, 1H), 4.15 (q, J = 7.1 Hz, 2H), 3.60 (s, 3H), 3.52 (d, J = 15.5 Hz, 1H), 3.42 (dd, J = 15.5 Hz, 7.0 Hz, 1H), 2.80 (d, J = 18.5 Hz, 1H), 2.61 (d, J = 16.0 Hz, 1H), 2.50 (d, J = 16.0 Hz, 1H), 2.48 (d, J = 18.4 Hz, 1H), 1.93-1.89 (m, 3H), 1.25 (t, J = 7.1 Hz, 3H), 1.13 (s, 3H); ^{13}C NMR (101 MHz, $CDCl_3$) δ 209.7, 171.5, 169.1, 161.4, 142.7, 137.1, 117.1, 61.2, 51.5, 44.7, 44.0, 41.1, 37.0, 24.4, 21.7, 14.1. HRMS (ESI): calc. for $C_{16}H_{22}NaO_5^+$ [M+Na]$^+$: 317.1359, found: 317.1365.

Ethyl 2-(4-(2-methoxy-2-oxoethyl)-2-(3-methylbut-2-en-2-yl)-3-oxocyclopent-1-en-1-yl)acetate ***(3ka)***

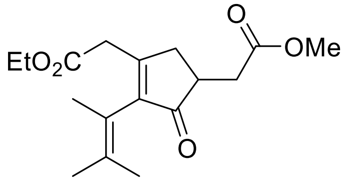

3ka

Eluent: PE/EA = 6/1 (+0.1% triethylamine). Following the general procedure (0.20 mmol scale), ***3ka*** (27.3 mg, 0.088 mmol, 44% yield) was obtained as a slightly yellow oil. ^{1}H NMR (400 MHz, $CDCl_3$) δ 4.15 (q, J = 7.1 Hz, 2H), 3.67 (app-d, 3H, two -OMe signals from a pair of rotamers), 3.31 (m, 2H), 3.03 – 2.90 (m, 1H), 2.89 – 2.73 (m, 2H), 2.61 (d, J = 16.0 Hz, 1H), 2.50 (d, J = 16.0 Hz, 1H), 2.54 – 2.73 (m, 2H), 1.76 (s, 3H), 1.69 (s, 3H), 1.48 – 1.43 (m, 3H), 1.25 (t, J = 7.1 Hz, 3H); ^{13}C NMR (101 MHz, $CDCl_3$) δ [208.0, 207.9], [172.4, 172.3], [169.1, 169.0], [163.2, 163.1], [145.6, 145.5], [131.9, 131.7], [120.0, 119.8], 61.1, 51.7, [41.9, 41.7], [37.1, 37.0], [36.4,

36.2], [35.1, 35.0], [21.8, 21.7], [19.89, 19.87], [17.92, 17.89], 14.1 (signals grouped within the same bracket might represent identical types of carbon atoms found in different rotamers of a molecule). HRMS (ESI): calc. for $C_{17}H_{24}NaO_5^+$ [M+Na]$^+$: 331.1516, found: 331.1525.

*Ethyl 2-(4-(2-methoxy-2-oxoethyl)-2-(3-methylbut-1-en-2-yl)-3-oxocyclopent-1-en-1-yl)acetate (**3kb**)*

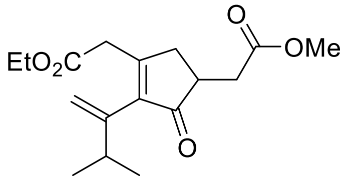

3kb

Eluent: PE/EA = 6/1 (+0.1% triethylamine). Following the general procedure (0.20 mmol scale), ***3kb*** (11.5 mg, 0.038 mmol, 19% yield) was obtained as a slightly yellow oil. ^{1}H NMR (400 MHz, $CDCl_3$) δ 5.20 – 5.18 (m, 1H), 4.81 – 4.79 (m, 1H), 4.17 (q, J = 7.1 Hz, 2H), 3.68 (s, 3H), 3.46 (s, 2H), 2.98 (dd, J = 18.3 Hz, 6.9 Hz, 1H), 2.85 (dd, J = 16.2 Hz, 4.2 Hz, 1H), 2.82 – 2.76 (m, 1H), 2.61 (sept, J = 6.9 Hz, 1H), 2.51-2.41 (m, 2H), 1.26 (t, J = 7.2 Hz, 3H), 0.99 (d, J = 6.9 Hz, 6H); ^{13}C NMR (101 MHz, $CDCl_3$) δ 207.5, 172.3, 169.2, 163.8, 147.4, 144.5, 113.7, 61.2, 51.8, 41.7, 37.1, 36.6, 35.0, 31.8, 21.1, 21.0, 14.1; HRMS (ESI): calc. for $C_{17}H_{24}NaO_5^+$ [M+Na]$^+$: 331.1516; found: 331.1521.

*Ethyl 2-(4-(2-methoxy-2-oxoethyl)-3-oxo-2-(1-phenylvinyl)cyclopent-1-en-1-yl)acetate (**3l**)*

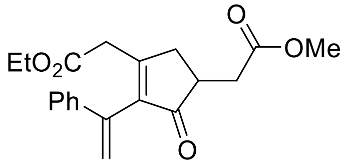

3l

Eluent: PE/EA = 6/1. Following the general procedure (0.20 mmol scale), ***3l*** (47.3 mg, 0.138 mmol, 69% yield) was obtained as a slightly yellow oil. ^{1}H NMR (400 MHz, $CDCl_3$) δ 7.37 – 7.16 (m, 5H), 5.81 (d, J = 1.2 Hz, 1H), 5.24 (d, J = 1.2 Hz, 1H), 4.10 (q, J = 7.1 Hz, 2H), 3.69 (s, 3H), 3.37

(s, 2H), 3.07 (dd, *J* = 18.5, 6.9 Hz, 1H), 2.94 – 2.82 (m, 2H), 2.65 – 2.50 (m, 2H), 1.22 (t, *J* = 7.1 Hz, 3H); ^{13}C NMR (101 MHz, $CDCl_3$) δ 206.9, 172.3, 168.8, 165.6, 142.7, 139.1, 138.8, 128.4, 128.0, 126.2, 118.0, 61.2, 51.8, 41.8, 37.2, 36.8, 34.8, 14.0. HRMS (ESI): calc. for $C_{20}H_{22}NaO_5^+$ $[M+Na]^+$: 365.1359, found: 365.1362.

2.3 Gram-Scale Synthesis for the Synthesis of 3c.

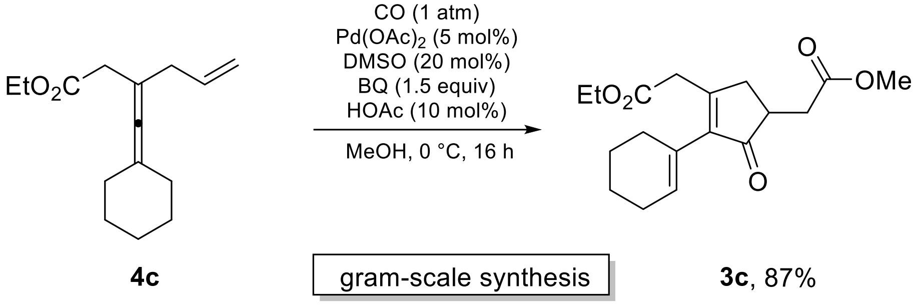

4c **3c**, 87%

In a Schlenk-flask with a magnetic stirring bar, $Pd(OAc)_2$ (47.9 mg, 0.21 mmol, 5 mol%) and BQ (691.9 mg, 6.40 mmol, 1.5 equiv) were dissolved in 10 ml MeOH. Enallene **4c** (1.00 g, 4.29 mmol), DMSO (60.4 µl, 0.85 mmol, 20 mol%), acetic acid (24.6 µl, 0.43 mmol, 10 mol%), and an additional 10 ml of MeOH were then added in sequence under a N_2 atmosphere. The flask was sealed with a septum and subjected to three cycles of evacuation and filling with CO gas using a balloon. The reaction was stirred rapidly at 0 °C for 16 hours. Upon complete consumption of the starting enallene **4c**, as confirmed by TLC, the reaction mixture was concentrated under reduced pressure and the product was purified by silica gel column chromatography. Eluent: PE/EA = 5/1 (+0.1% triethylamine). Compound **3c** (1.19 g, 3.71 mmol, 87% yield) was obtained as a slightly yellow oil. The ^{1}H NMR spectrum was in good accordance with **3c** from the small-scale synthesis.

2.4 General Procedure for the Small-scale Sequential Synthesis of Vinyl-Cyclopentenone 2 from TsO- functionalized Enallenes.

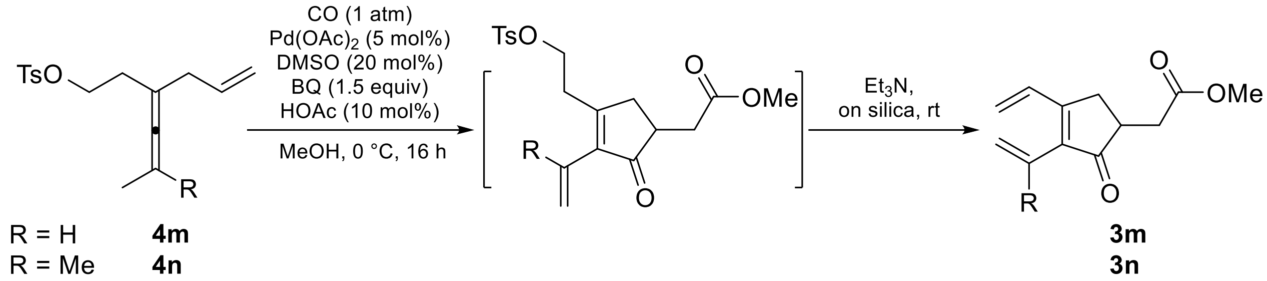

These reactions were conducted without special precautions to exclude moisture. In a vial with a magnetic stirring bar, $Pd(OAc)_2$ (2.2 mg, 0.01 mmol, 5 mol%) and BQ (32.4 mg, 0.30 mmol, 1.5 equiv) were dissolved in 0.5 ml MeOH. Enallene **4** (0.20 mmol), DMSO (2.9 µl, 0.04 mmol, 20 mol%), acetic acid (1.2 µl, 0.02 mmol, 10 mol%), and an additional 0.5 ml of MeOH were then added in sequence. The vial was sealed with a septum and subjected to three cycles of evacuation and filling with CO gas using a balloon. The reaction was stirred at 0 °C for 16 hours. Upon complete consumption of the starting enallene **4**, as confirmed by TLC, the reaction mixture was concentrated under reduced pressure and the product was purified by silica gel column chromatography using eluent with 0.1% triethylamine.

*Methyl 2-(2-oxo-3,4-divinylcyclopent-3-en-1-yl)acetate (**3m**)*

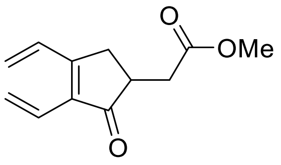

3m

Eluent: PE/EA = 10/1 to 3/1 (+0.1% triethylamine). Following the general procedure (0.20 mmol scale), ***3m*** (26.1 mg, 0.126 mmol, 63% yield) was obtained as a colorless oil. ^{1}H NMR (400 MHz, $CDCl_3$) δ 7.05 (dd, J = 17.3, 10.7 Hz, 1H), 6.54 (dd, J = 17.6, 11.6 Hz, 1H), 6.28 (dd, J = 17.6, 2.1 Hz, 1H), 5.80 (dd, J = 17.3, 1.0 Hz, 1H), 5.58 (d, J = 11.0 Hz, 1H), 5.46 (dd, J = 11.6, 2.1 Hz, 1H), 3.70 (s, 3H), 3.06 (dd, J = 18.1, 7.4 Hz, 1H), 2.91 (dd, J = 16.5, 4.1 Hz, 1H), 2.86 – 2.80 (m, 1H), 2.61 – 2.35 (m, 2H); ^{13}C NMR (101 MHz, $CDCl_3$) δ 207.9, 172.6, 162.2, 134.0, 130.5, 125.0,

122.5, 121.1, 51.8, 41.5, 35.3, 31.9. HRMS (ESI): calc. for $C_{12}H_{14}NaO_3^+$ $[M+Na]^+$: 229.0835, found: 229.0836.

*methyl 2-(2-oxo-3-(prop-1-en-2-yl)-4-vinylcyclopent-3-en-1-yl)acetate (**3n**)*

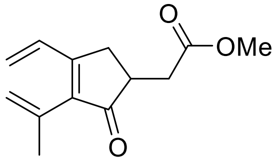

3n

Eluent: PE/EA = 3/1 (+0.1% triethylamine). Following the general procedure (0.20 mmol scale), ***3n*** (37.0 mg, 0.168 mmol, 84% yield) was obtained as a colorless oil. ^{1}H NMR (400 MHz, $CDCl_3$) δ 7.03 – 6.94 (m, 1H), 5.77 (dd, *J* = 17.5 Hz, 1.0 Hz, 1H), 5.50 (dd, *J* = 10.6 Hz, 0.9 Hz, 1H), 5.26 – 5.23 (m, 1H), 4.86 – 4.84 (m, 1H), 3.69 (s, 3H), 3.06 (dd, *J* = 17.5 , 7.2 Hz, 1H), 2.89 (dd, *J* = 16.4 Hz, 4.1 Hz, 1H), 2.84-2.77 (m, 1H), 2.47-2.38 (m, 2H), 1.95-1.93 (m, 3H); ^{13}C NMR (101 MHz, $CDCl_3$) δ 207.6, 172.5, 162.3, 142.3, 136.9, 131.8, 121.9, 118.0, 51.8, 41.2, 35.3, 32.3, 21.9. HRMS (ESI): calc. for $C_{13}H_{16}NaO_3$ $[M+Na]^+$: 243.0992, found: 243.0999.

2.5 Gram-Scale Synthesis of 3m

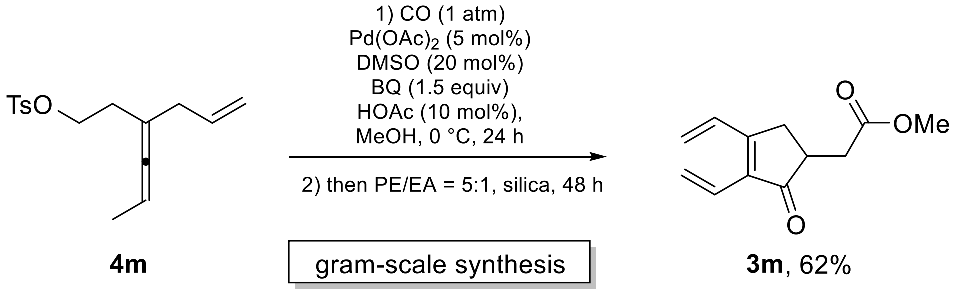

In a Schlenk-flask with a magnetic stirring bar, $Pd(OAc)_2$ (46.1 mg, 0.20 mmol, 5 mol%) and BQ (665.4 mg, 6.16 mmol, 1.5 equiv) were dissolved in 10 ml MeOH. Enallene (1.2 g, 4.10 mmol), DMSO (58.3 µl, 0.82 mmol, 20 mol%), acetic acid (23.5 µl, 0.41 mmol, 10 mol%), and an additional 10 ml of MeOH were then added in sequence under N_2. The flask was sealed with a septum and subjected to three cycles of evacuation and filling with CO gas using a balloon. The

reaction was stirred rapidly at 0 °C for 24 hours. Upon complete consumption of the starting enallene **4m**, as confirmed by TLC. The reaction mixture was concentrated under reduced pressure. In a round bottom flask, the intermediate, PE/EA 5:1 (100 mL), silica gel (10 g), and Et_3N (10 mL) were added. The reaction mixture was stirred rapidly for 48 hours at room temperature. After full consumption of starting material monitored by TLC, the reaction mixture was evaporated and purified via short column chromatography on silica gel with eluent: PE/EA = 5/1 (+0.1% triethylamine) to give the product (523.8 mg, 2.54 mmol, 62% yield). The ^1H NMR spectrum was in good accordance with **3m** from the small-scale synthesis.

2.6 Decagram-Scale Synthesis of 3n

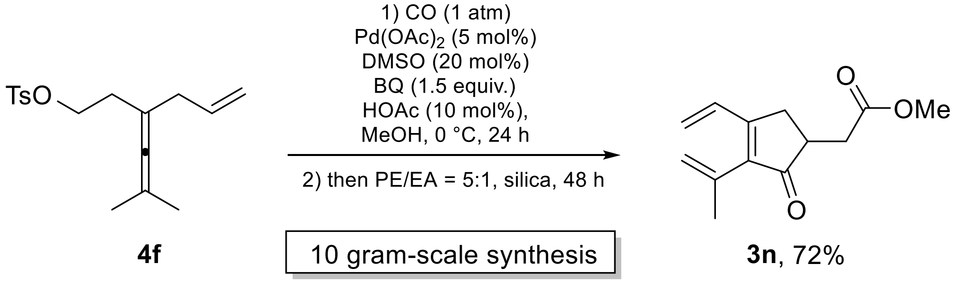

4f **3n**, 72%

In a round-bottom flask with a magnetic stirring bar, $Pd(OAc)_2$ (370.0 mg, 1.65 mmol, 5 mol%) and BQ (5.34 g, 49.4 mmol, 1.5 equiv.) were dissolved in 50 ml MeOH. Enallene **4f** (10.10 g, 32.96 mmol), DMSO (468 µl, 6.59 mmol, 20 mol%), acetic acid (189 µl, 0.33 mmol, 10 mol%), and an additional 50 ml of MeOH were then added in sequence under a N_2 atmosphere. The flask was sealed with a septum and subjected to three cycles of evacuation and filling with CO gas using a balloon. The reaction was stirred rapidly at 0 °C for 24 hours. Upon complete consumption of the starting enallene **4f**, as confirmed by TLC. The reaction mixture was concentrated under reduced pressure. In a round bottom flask, the intermediate, PE/EA 5:1 (100 mL), silica gel (10 g), and Et_3N (10 mL) were added. The reaction mixture was stirred rapidly for 48 hours at room temperature. After full consumption of starting material monitored by TLC, the reaction mixture was evaporated and purified via short column chromatography on silica gel with eluent: PE/EA = 5/1 (+0.1% triethylamine) to give the product **3n** (5.23 g, 23.7 mmol, 72% yield). The ^1H NMR spectrum was in good accordance with **3n** from the small-scale synthesis.

2.7 Kinetic Isotope Effect (KIE) Experiments and Proposed Mechanism

Determination of the Competition KIE

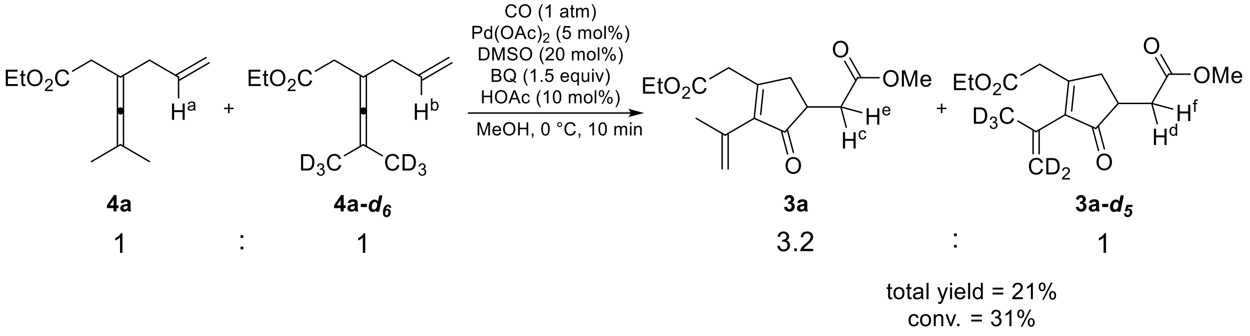

In a vial with a magnetic stirring bar, $Pd(OAc)_2$ (2.2 mg, 0.01 mmol, 5 mol%) and BQ (32.4 mg, 0.30 mmol, 1.5 equiv) were dissolved in 0.5 ml MeOH. Enallene **4a** (19.4 mg, 0.10 mmol), Enallene **4a-*d₆*** (20.0 mg, 0.10 mmol), DMSO (2.9 µl, 0.04 mmol, 20 mol%), acetic acid (1.2 µl, 0.02 mmol, 10 mol%), and an additional 0.5 ml of MeOH were then added in sequence. The vial was sealed with a septum and subjected to three cycles of evacuation and filling with CO gas using a balloon. The reaction was stirred at 0 °C for 10 min, then quickly evaporated. The yields and the ratio of **3a** and **3a-*d₅*** were determined by ^{1}H NMR measurement using anisole as the internal standard (22 µl, 0.20 mmol). As shown in the attached spectrum, the combined yield of **3a** and **3a-d₅** was 21% (integration of signals around 2.98 and 2.88 ppm, respectively, arising from H_c+H_d and H_e+H_f, respectively). The NMR yield of **3a** was determined as 16% (by integration of the signal at δ 5.23), in conclusion the yield of **3a-d₅** was 5% and therefore the ratio of **3a** and **3a-d₅** is 3.2:1. The combined recovery of **4a** and **4a-d₆** was 69% (integration of the signal of H_a+H_b at δ 5.79), so the reaction conversion was 31%. Additionally, recovery of **4a** was 25% (integration of the singlet at δ 1.69 from **4a** is 1.5). Therefore, the remaining **4a-d₆** was 44%. Consequently, the ratio of recovered **1a** and **4a-d₆** was determined as 1:1.8. Finally, the competitive kinetic isotope effect (KIE) value calculated from the product ratio and the change of the starting material ratio is 4.2 according to Sih's equation.[7]

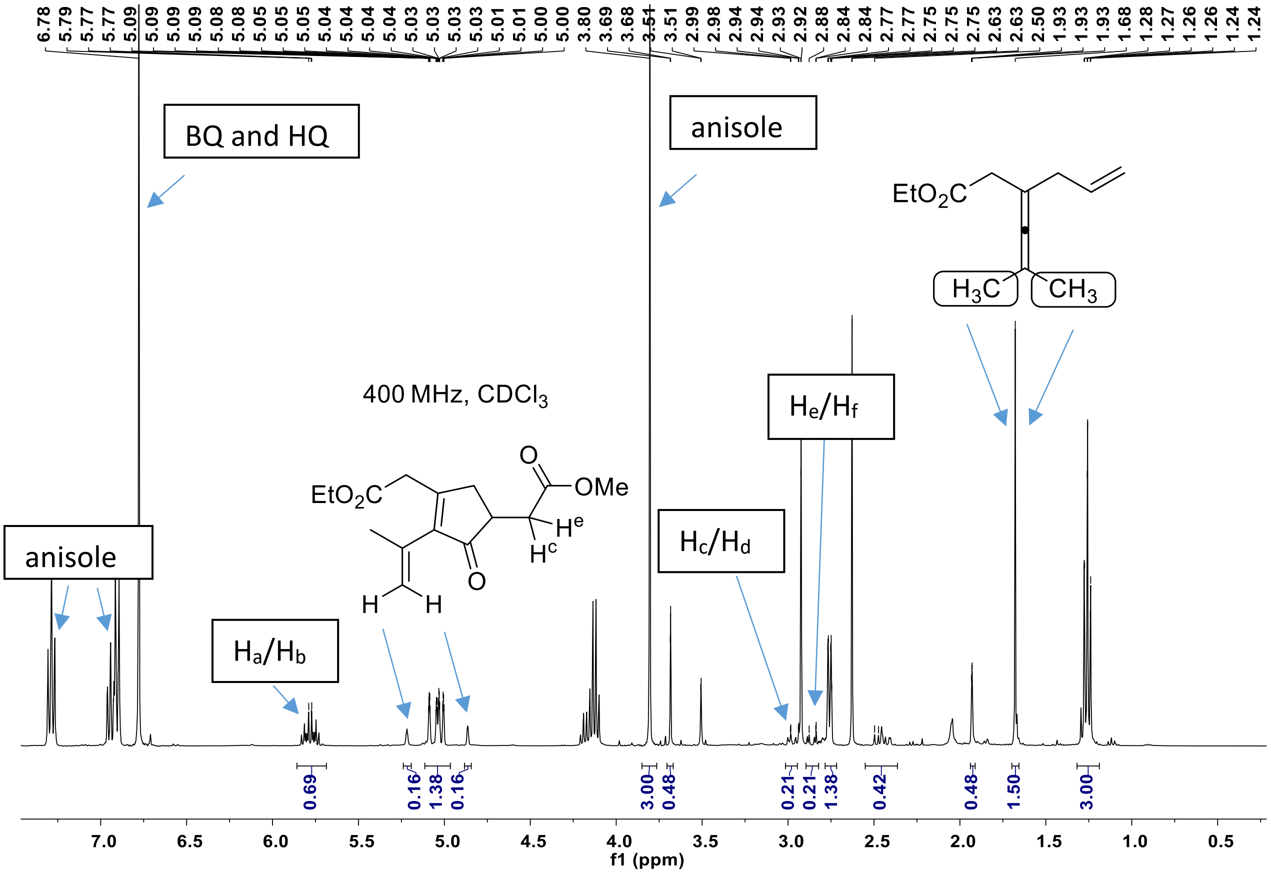

Determination of the Parallel KIE (Separate Experiments)

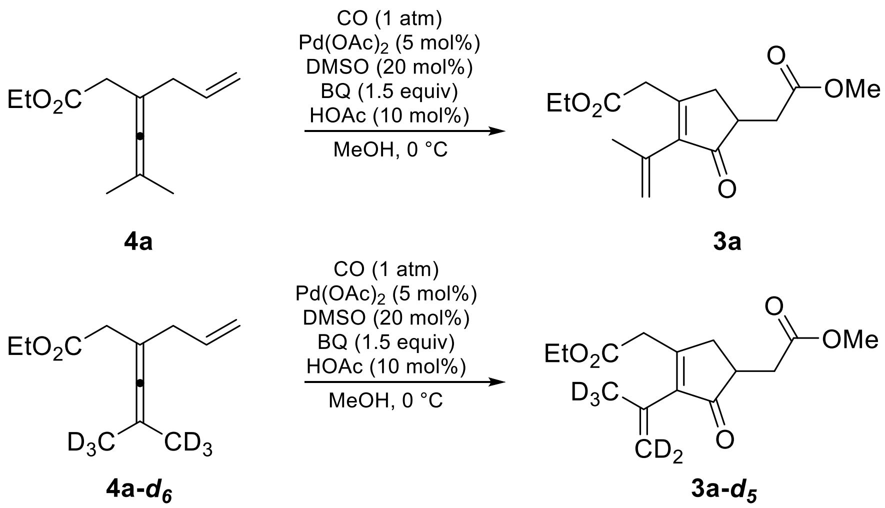

In a vial with a magnetic stirring bar, $Pd(OAc)_2$ (2.2 mg, 0.01 mmol, 5 mol%) and BQ (32.4 mg, 0.30 mmol, 1.5 equiv) were dissolved in 0.5 ml MeOH. Enallene **4a** (38.9 mg, 0.20 mmol) [or enallene **4a-*d*₆** (40.1 mg, 0.20 mmol)], DMSO (2.9 µl, 0.04 mmol, 20 mol%), acetic acid (1.2 µl,

0.02 mmol, 10 mol%), and an additional 0.5 ml of MeOH were then added in sequence. The vial was sealed with a septum and subjected to three cycles of evacuation and filling with CO gas using a balloon. The reaction was stirred at 0 °C and recorded at different times (see Table S4 and S5, respectively). The yields were determined by ^{1}H NMR measurement using anisole as the internal standard.

***Table S4*:** Progress of the reaction to give **3a** at the early stage of the reaction

Time [min]	1	3	5	8	10
Yield of **3a** [%]	3.4	5.5	11.0	19.1	28.1

Due to the nature of the experiment, plots to determine the KIE were taken for **3a** (figure S1 and S2).

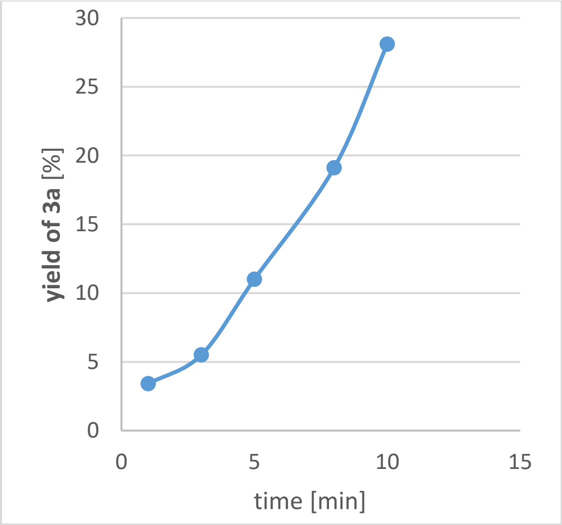

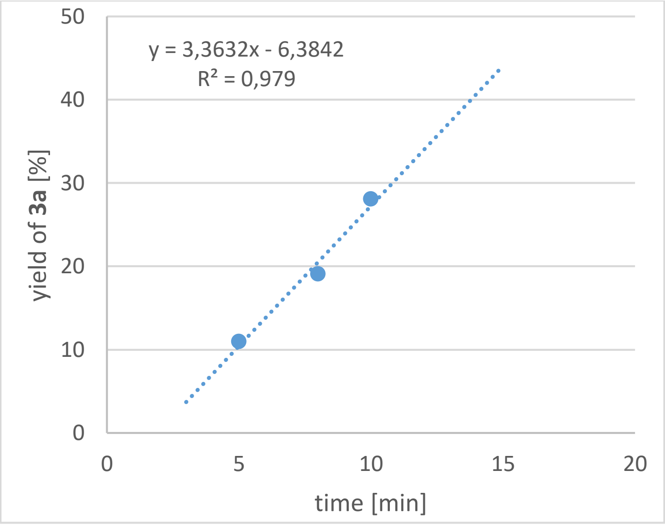

Figure S1: Progress of the reaction to give **3a** at the early stage of the reaction (left); Linear function fit for reaction rate to give **3a** at the early stage of the reaction (right)

Table S5: Progress of the reaction to give **3a-*d*$_5$** at the early stage of the reaction

Time [min]	2	5	10	17.5	25
Yield of **3a-d$_5$** [%]	4.8	9.4	16.6	28.2	42.2

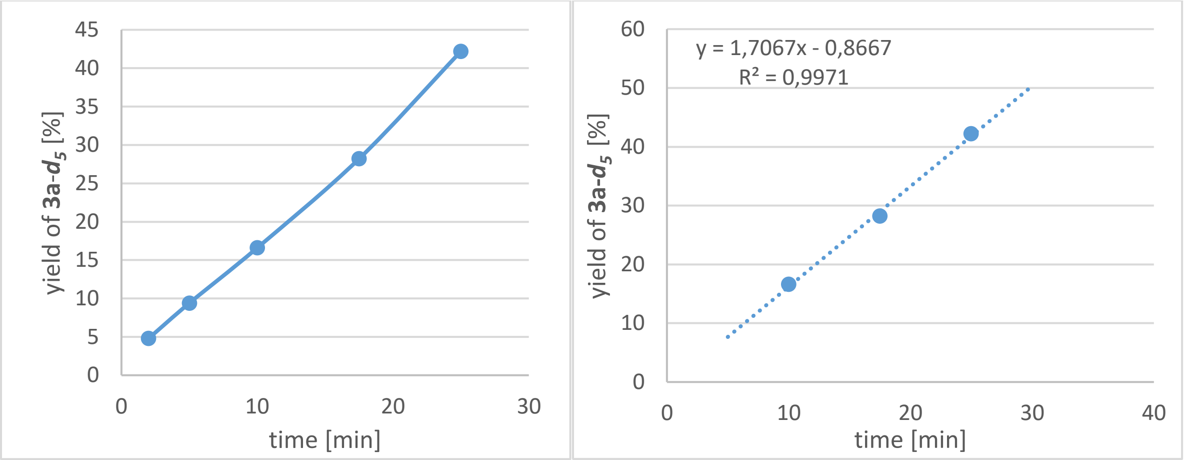

Figure S2: Progress of the reaction to give **3a-*d*$_5$** in the early stage; Linear function fit for the reaction rate of **3a-*d*$_5$** at the early stage of the reaction (right).

Since there is an induction period in the reactions there will be some errors in the linearity. Part of that error is cancelled when calculating k_H/k_D from the ratio of the slopes. We estimate that the parallel kinetic isotope effect value is 2.0 ± 0.1.

$$\frac{k_H}{k_D} = \frac{3.3632}{1.7067} = 2.0 \pm 0.1$$

2.8 Proposed Mechanism

Building on the successful carbon monoxide insertion cascade and the KIE study, we propose a mechanism depicted in Scheme S1. The cascade begins with the coordination of enallene **4** to Pd^{2+}(L) forming the intermediate *Int*-**S1**. This is followed by the attack of allene on Pd^{2+}, leading to the formation of *Int*-**S2**. According the KIE study, the formation of *Int*-**S2** is the rate-determine step (RDS). CO insertion into *Int*-**S2** produces *Int*-**S3**. The subsequent migratory insertion of the olefin into the carbonyl-Pd^{2+} bond yields the carbocyclic intermediate *Int*-**S4**. Carbonylation *Int*-**S4** result in the formation of *Int*-**S5**. *Int*-**S5** reacts with methoxy, resulting in the carbonylated

cyclopentenone **3**. Finally, the generated Pd^0 is reoxidized by BQ to Pd^{2+}, thereby completing the catalytic cycle.

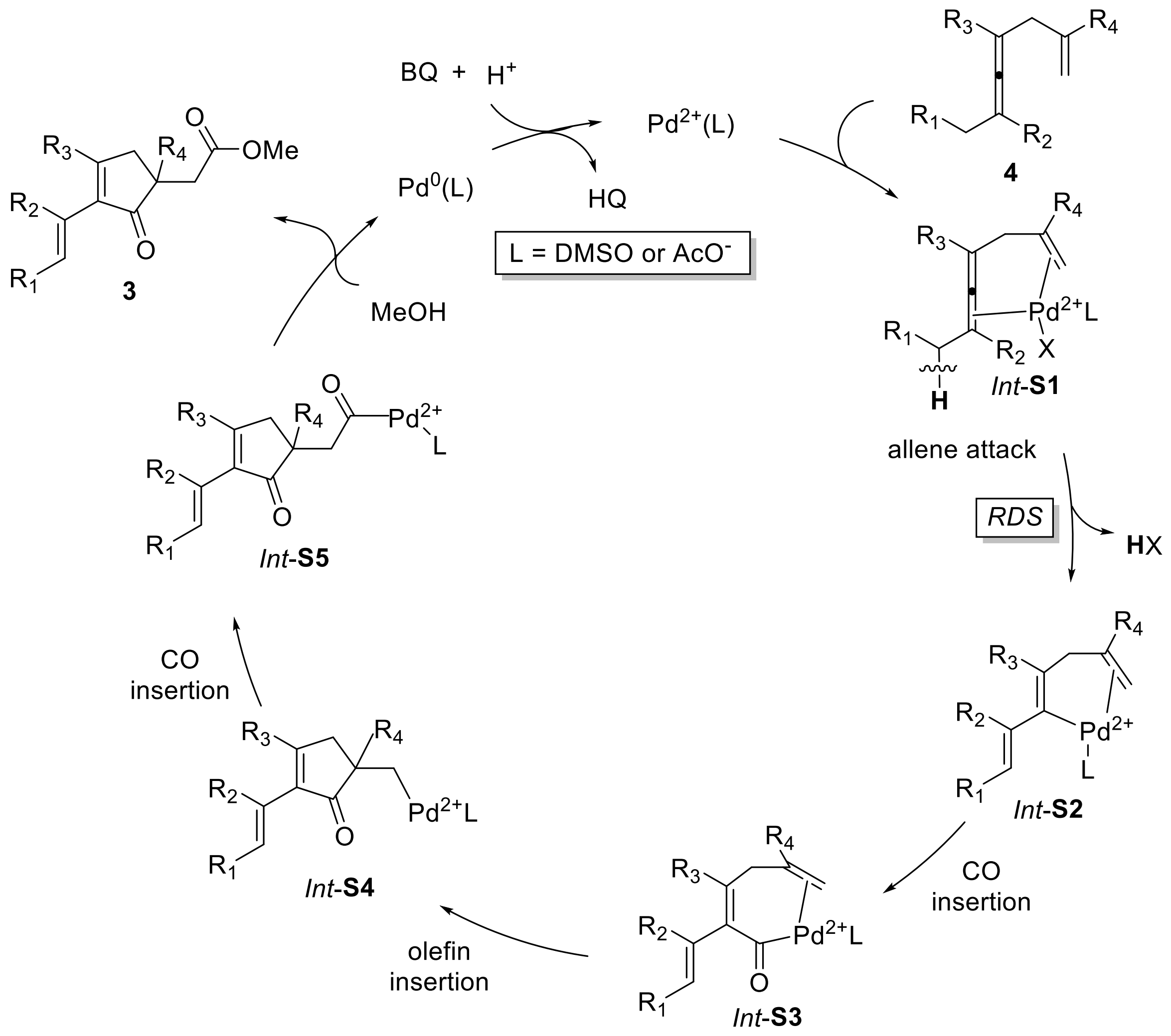

Scheme S1: Proposed Mechanism

3. *The Construction of A-ring Moiety of SL*

3.1 Percyclization Reaction for A-ring Construction

Table S6: Effect of temperature additive for the percyclization reaction of triene[a]

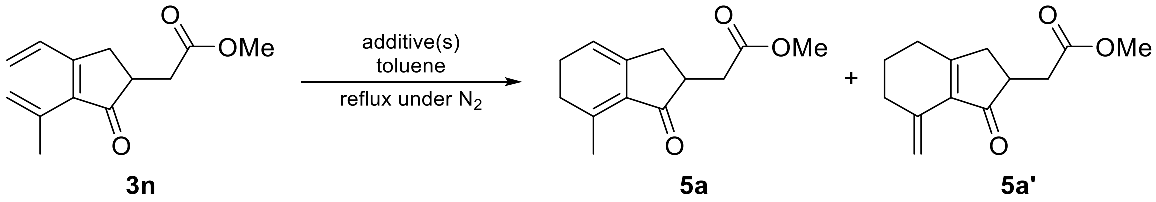

Entry	Additive(s)	Yields (%)[b] of **5a**	Yield (%)[b] of **5a'**
1	No	39	30
2	NaOAc (1 equiv.)	39	6
3	NaOAc (20 mol%)	58	10
4	NaOAc (10 mol%)	44	6
5	NaOAc (5 mol%)	56	8
6	NaOAc (1 mol%)	54	3
**7**	**NaOAc (~1 mol%) + H_2O (1.1eq)**	**97 (92)**	**1**
8	Propanoic acid (5 mol%)	41	25
9	Et_3N (5%)	52	3
10	NaOAc (1%) + H_2O (5 equiv.)	63	0

a) In a vial with a magnetic stirring bar, triene **3n** (0.1 mmol) and indicated additive were dissolved in 0.5 ml toluene under N_2. The vial was sealed with a septum and subjected to three cycles of evacuation and filling with N_2 gas. Then, the reaction was stirred at reflux for 24 hours. b) The yield was determined using ^{1}H-NMR analysis with 1,3,5-trimethoxybenzene as the internal standard. The numbers in parentheses indicate the isolated yield.

Crude NMR of entry 1

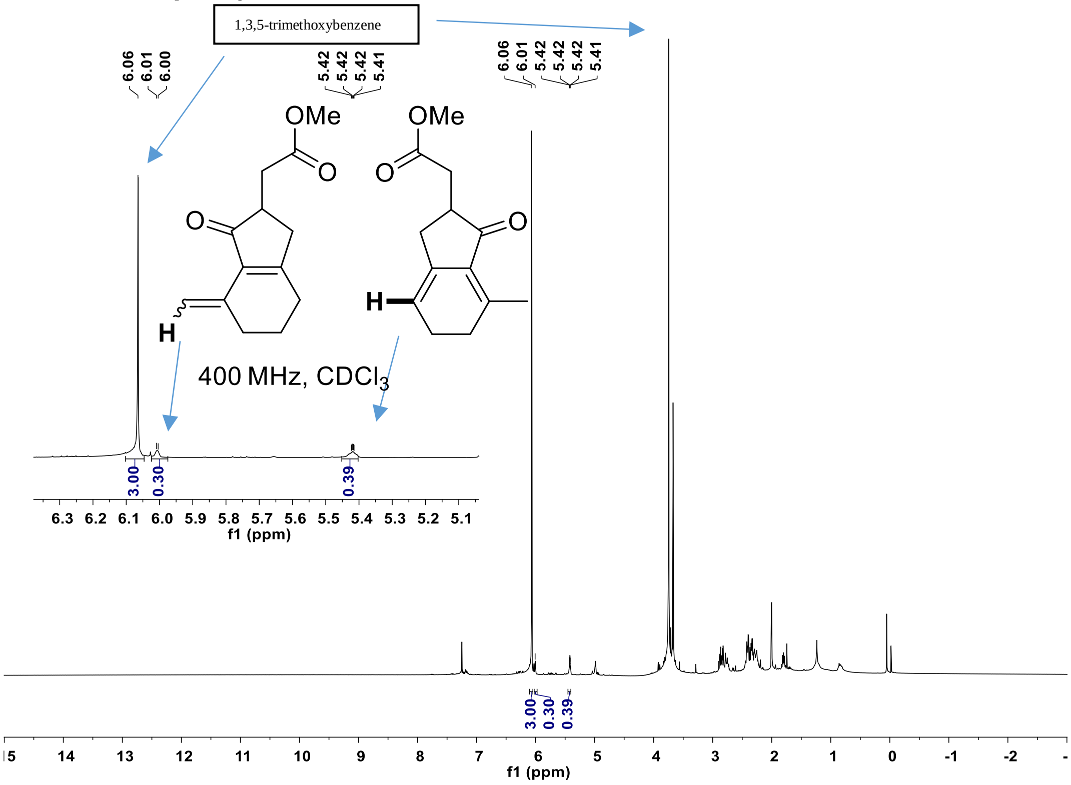

Crude NMR of entry 7

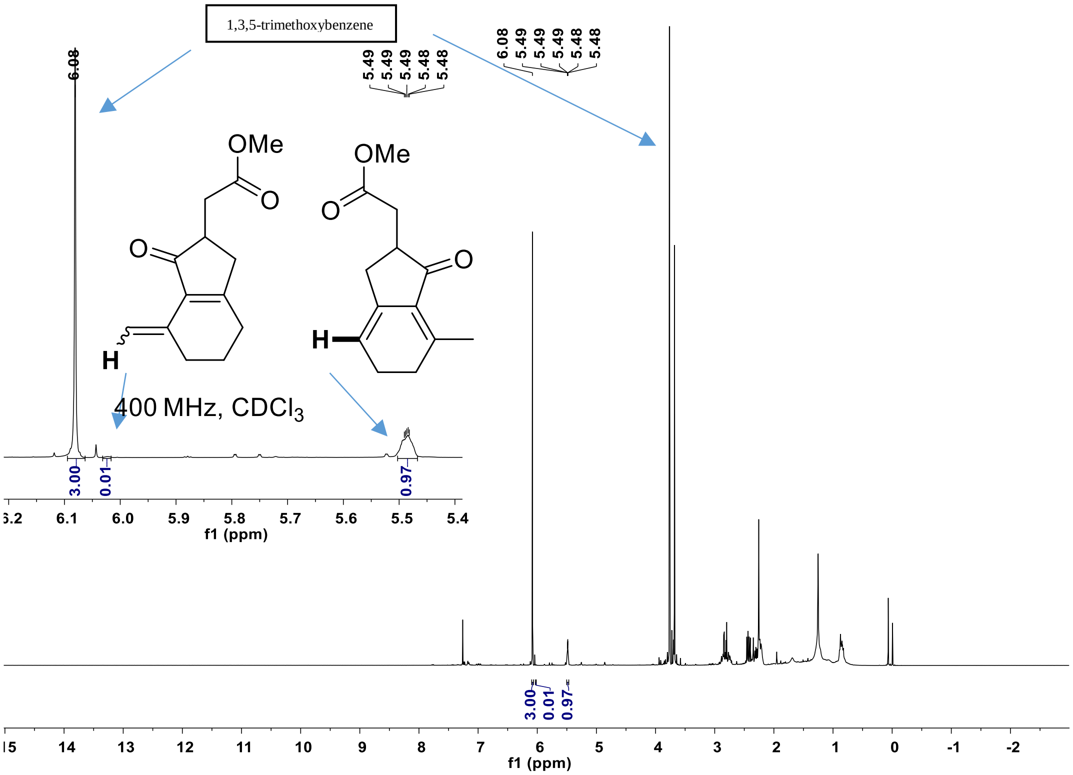

NMR of Enriched 5a’

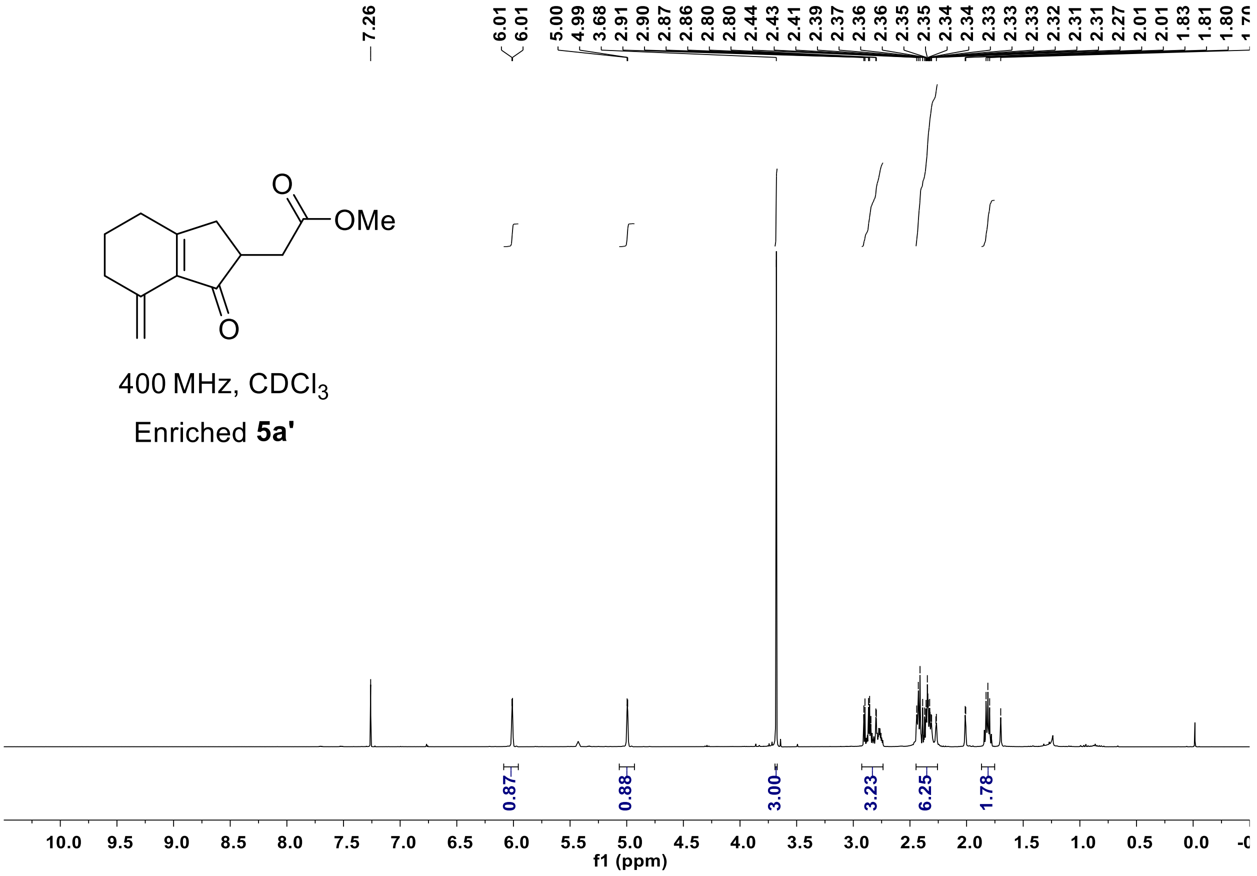

Methyl 2-(7-methyl-1-oxo-2,3,5,6-tetrahydro-1H-inden-2-yl)acetate **5a**

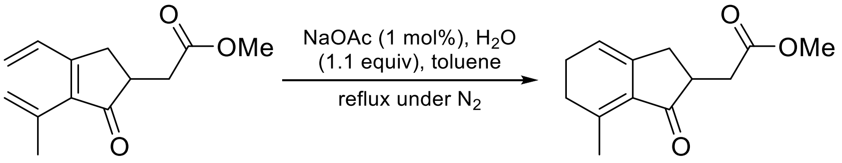

3n **5a**, 92%

In a vial with a magnetic stirring bar, triene **3n** (0.2 mmol, 44.1 mg), NaOAc (0.002 mmol, 0.2 mg, 1 mol%), and H_2O (0.22 mmol, 4 μL, 1.1 equiv) were added in 1 ml toluene under N_2. The vial was sealed and subjected to three cycles of evacuation and filling with N_2 gas. Then, the reaction was stirred at reflux for 24 hours. The reaction mixture was concentrated under reduced pressure and the product was purified by silica gel column chromatography (PE:EA = 10:1). Compound **5a** (40.5 mg, 0.184 mmol, 92% yield) was obtained as a colorless oil. ^{1}H NMR (400 MHz, $CDCl_3$) δ

5.59 – 5.32 (m, 1H), 3.67 (s, 3H), 2.89 – 2.70 (m, 3H), 2.46 – 2.27 (m, 3H), 2.27 – 2.16 (m, 6H); ^{13}C NMR (101 MHz, $CDCl_3$) δ 205.5, 172.8, 149.1, 135.2, 129.2, 115.1, 51.7, 45.2, 34.9, 31.9, 31.3, 22.2, 19.2. HRMS (ESI): calc. for $C_{13}H_{16}NaO_3$ $[M+Na]^+$: 243.0992, found: 243.0994.

Gram Scale Synthesis of Methyl 2-(7-methyl-1-oxo-2,3,5,6-tetrahydro-1H-inden-2-yl)acetate **5a**

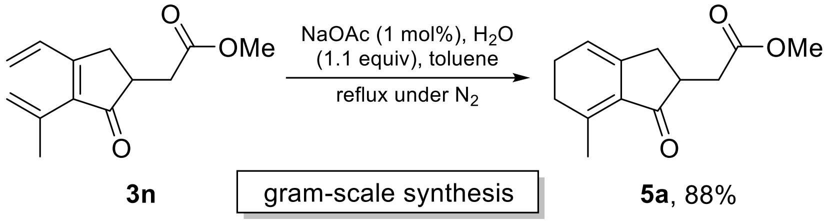

3n **5a**, 88%

In a round bottom flask with a magnetic stirring bar, triene **3n** (4.6 mmol, 1.02 g), NaOAc (0.046 mmol, 3.8 mg, 1 mol%), and H_2O (5.1 mmol, 91 µL, 1.1 equiv) were added in 23 ml toluene under N_2. After adding of a condenser, the set-up was sealed with a septum and subjected to three cycles of evacuation and filling with N_2 gas. Then, the reaction was stirred at reflux for 24 hours. The reaction mixture was concentrated under reduced pressure and the product was purified by silica gel column chromatography (PE:EA = 10:1). Compound **5a** (897.6 mg, 4.08 mmol, 88% yield) was obtained as a colorless oil. The ^1H NMR spectrum was in good accordance with **5a** from the small-scale synthesis.

3.2 Functionalization of the A-ring moieties

Methyl 2-(7,7-dimethyl-1-oxo-2,3,4,5,6,7-hexahydro-1H-inden-2-yl)acetate **6a**

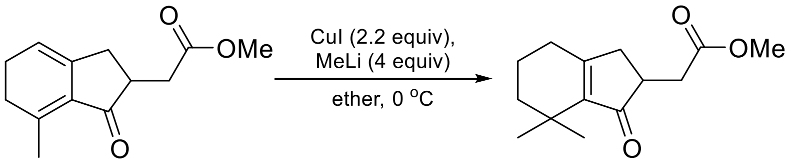

5a **6a**, 97%

In a Schlenk flask, copper(I) iodide (620 mg, 3.26 mmol, 2.2 equiv) and dry Et_2O (4 mL) were introduced. Under a nitrogen atmosphere at 0 °C, methyl lithium (3.7 mL, 5.92 mmol, 4 equiv) was added and the mixture was stirred for 30 minutes. Subsequently, a solution of compound **5a** (325 mg, 1.48 mmol, 1 equiv) in dry Et_2O (2 mL) was added. The reaction mixture was then stirred

at 0 °C for another 30 minutes. The complete consumption of the starting material was monitored by TLC. The reaction was quenched with a saturated NH_4Cl solution (4 mL). The organic layer was separated, and the aqueous phase was extracted with Et_2O. The combined organic extracts were dried over Na_2SO_4, filtered, and the solvent was evaporated under reduced pressure to yield the product **6a** (339 mg, 1.43 mmol, 97% yield). ^{1}H NMR (400 MHz, $CDCl_3$) δ 3.66 (s, 3H), 2.80 (dd, *J* = 16.4, 4.2 Hz, 1H), 2.73 – 2.59 (m, 2H), 2.33 (dd, *J* = 16.4, 9.3 Hz, 1H), 2.27 – 2.21 (m, 2H), 2.20 – 2.09 (m, 1H), 1.75 – 1.64 (m, 2H), 1.51 – 1.39 (m, 2H), 1.16 (s, 3H), 1.15 (s, 3H); ^{13}C NMR (101 MHz, $CDCl_3$) δ 207.9, 172.8, 171.0, 144.0, 51.7, 41.8, 39.2, 36.5, 35.2, 31.1, 29.1, 26.6, 26.5, 19.0. HRMS (ESI): calc. for $C_{14}H_{20}NaO_3^+$ $[M+Na]^+$: 259.1305, found: 259.1304.

Methyl 2-(7,7-dimethyl-1,4-dioxo-2,3,4,5,6,7-hexahydro-1H-inden-2-yl)acetate ***6b***

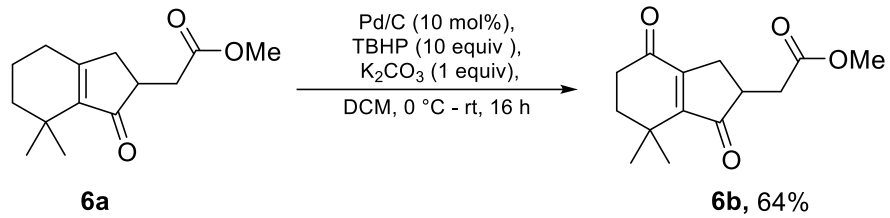

To a solution of compound **6a** (590 mg, 2.5 mmol) in DCM (25 mL), 10% palladium on carbon (266 mg, 0.25 mmol, 10 mol%) and K_2CO_3 (345 mg, 2.5 mmol, 1.0 equiv) were added. The mixture was cooled to 0 °C prior to the addition of tert-butyl hydroperoxide (TBHP, 3.4 mL of a 70 wt% solution in H_2O, 25 mmol, 10 equiv). The mixture was then stirred at room temperature for 16 hours. Subsequent to stirring, the mixture was filtered through a celite pad. The filtrate was concentrated under reduced pressure to obtain the crude product, which was then purified using silica gel column chromatography (eluent: pentane/EA = 6:1) to yield the product **6b** (400 mg, 1.60 mmol, 64% yield) as a colorless oil. ^{1}H NMR (400 MHz, $CDCl_3$) δ 3.63 (s, 3H), 2.90 (m, 1H), 2.81 – 2.72 (m, 1H), 2.70 – 2.50 (m, 4H), 2.33 – 2.21 (m, 1H), 1.89 (td, *J* = 7.0, 1.7 Hz, 2H), 1.30 (s, 6H). ^{13}C NMR (101 MHz, $CDCl_3$) δ 209.9, 199.1, 171.8, 158.1, 156.2, 51. 8, 42.3, 38.2, 35.3, 34.4, 32.1, 29.1, 25.4, 24.9. These spectra data were in good accord with those reported in literature.[8]

Methyl 2-((4S)-4-hydroxy-7,7-dimethyl-1-oxo-2,3,4,5,6,7-hexahydro-1H-inden-2-yl)acetate ***6c***

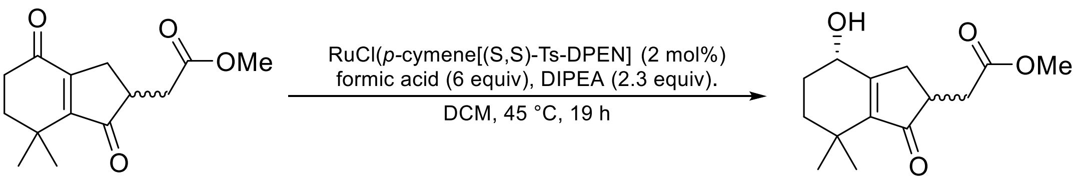

6b **6c**, 93%

In an 8 mL vial equipped with a magnetic stir bar, N, N-diisopropylethylamine (0.60 mL, 3.4 mmol, 2.3 equiv) was added. Formic acid (0.34 mL, 9.0 mmol, 6.0 equiv) was then introduced dropwise, and the resulting mixture was stirred at room temperature for 10 minutes. Next, a solution of compound **6b** (375 mg, 1.5 mmol, 1.0 equiv) in DCM (1.0 mL) was added, followed by evaporation under a nitrogen flow. Subsequently, RuCl(*p*-cymene)[(S,S)-Ts-DPEN] (19.0 mg, 0.03 mmol, 2 mol%) was introduced. The mixture was then stirred at 45 °C for 18 hours. The reaction mixture was directly subjected to silica gel column chromatography (eluent: EA/pentane = 1:1) to yield product 6 (352 mg, 93% yield) as a colorless oil. The ^{1}H NMR spectrum could not be resolved for two diastereomers, the distinctive peaks are 2.12 ppm and 3.05 ppm. ^{1}H NMR (400 MHz, $CDCl_3$) δ 4.34 (m, 1H), 3.64 (s, 3H), 3.12 – 3.00 (m, 1H), 2.83 – 2.55 (m, 3H), 2.52 – 2.33 (m, 2H), 1.99 (m, 1H), 1.76 – 1.38 (m, 3H), 1.15 (s, 6H). First diastereomer: ^{13}C NMR (101 MHz, $CDCl_3$) δ 208.8, 172.7, 169.8, 144.8, 67.7, 51.7, 42.2, 36.3, 34.8, 32.6, 31.4, 29.0, 26.3, 25.9. Second diastereomer ^{13}C NMR (101 MHz, $CDCl_3$) δ 208.5, 172.5, 170.3, 144.7, 68.3, 51.7, 41.9, 36.8, 35.1, 32.9, 31.4, 29.1, 26.6, 25.8.

3.3 Oxidative Percyclization reaction for A-ring Construction of GR-24

Methyl 2-(1-oxo-2,3-dihydro-1H-inden-2-yl)acetate ***5b***

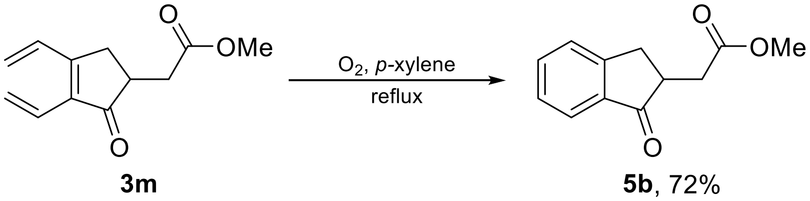

3m **5b**, 72%

In a round bottom flask with a magnetic stirring bar, triene **3m** (2.42 mmol, 500.1 mg) were added in 12 ml *p*-xylene under open-air. After adding of a condenser, the reaction mixture was stirred rapidly at reflux for 24 hours under open air. The reaction mixture was concentrated under reduced pressure and the product was purified by silica gel column chromatography (PE:EA = 10:1).

Compound **5b** (356.4 mg, 1.75 mmol, 72% yield) was obtained as a colorless oil. It is interesting to note that this oxidative cyclization is low-yielding when use toluene as the solvent. ^{1}H NMR (400 MHz, $CDCl_3$) δ 7.81 – 7.74 (m, 1H), 7.64 – 7.56 (m, 1H), 7.49 – 7.43 (m, 1H), 7.43 – 7.36 (m, 1H), 3.69 (s, 3H), 3.47 (dd, J = 17.0, 8.1 Hz, 1H), 3.13 – 2.95 (m, 2H), 2.89 (dd, J = 17.2, 4.4 Hz, 1H), 2.69 – 2.55 (m, 1H). ^{13}C NMR (101 MHz, $CDCl_3$) δ 206.6, 172.4, 153.2, 136.2, 134.8, 127.4, 126.5, 123.9, 51.8, 43.4, 34.9, 32.9; HRMS (ESI): calc. for $C_{12}H_{12}NaO_3^+$ $[M+Na]^+$: 227.0679, found: 227.0678.

4. Construction of the C-Ring and D-Ring Moieties of Strigols

Rac-(3aR,8bS)-8,8-dimethyl-3,3a,4,5,6,7,8,8b-octahydro-2H-indeno[1,2-b]furan-2-one (*rac*-***2a***)

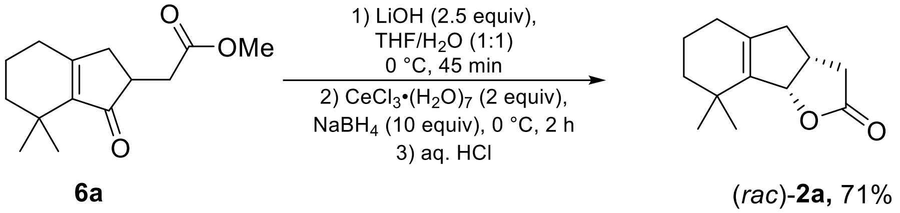

To a solution of compound **6a** (400.2 mg, 1.69 mmol, 1 equiv) in THF (6 mL), LiOH (101.4 mg, 4.24 mmol, 2.5 equiv) in water (6 mL) was added at 0 °C, and the mixture was stirred for 45 minutes. At 0 °C, $CeCl_3{\cdot}7H_2O$ (1.2622 g, 3.39 mmol, 2 equiv) was introduced, followed by the addition of a solution of $NaBH_4$ (640.7 mg, 16.9 mmol, 10 equiv) in cold water (5 mL). The mixture was stirred at 0 °C for 2 hours before the careful addition of a 2 M hydrochloric acid (HCl) solution. The mixture was then extracted with DCM (3 × 20 mL). The combined organic layers were dried over Na_2SO_4 and the solvent was evaporated under reduced pressure. The crude product was purified using silica gel column chromatography (eluent: EA/PE = 1/5) to yield product *rac*-**2a** (247.5 mg, 1.20 mmol, 71% yield). ^{1}H NMR (400 MHz, $CDCl_3$) δ 5.51 – 5.41 (m, 1H), 3.12 – 2.94 (m, 1H), 2.78 (dd, J = 18.2, 10.3 Hz, 1H), 2.67 – 2.52 (m, 1H), 2.31 (dd, J = 18.2, 4.9 Hz, 1H), 2.20 – 2.08 (m, 1H), 2.05 – 1.84 (m, 2H), 1.76 – 1.59 (m, 2H), 1.55 – 1.30 (m, 2H), 1.09 (s, 3H), 1.07 (s, 3H). ^{13}C NMR (101 MHz, $CDCl_3$) δ 177.6, 141.5, 139.9, 90.2, 42.3, 38.9, 36.2, 34.4, 31.9, 28.0, 27.7, 26.4, 19.2. HRMS (ESI): calc. for $C_{13}H_{18}NaO_2^+$ $[M+Na]^+$: 229.1199, found: 229.1198.

(3aR,5S,8bS)-5-hydroxy-8,8-dimethyl-3,3a,4,5,6,7,8,8b-octahydro-2H-indeno[1,2-b]furan-2-one (**2b**)

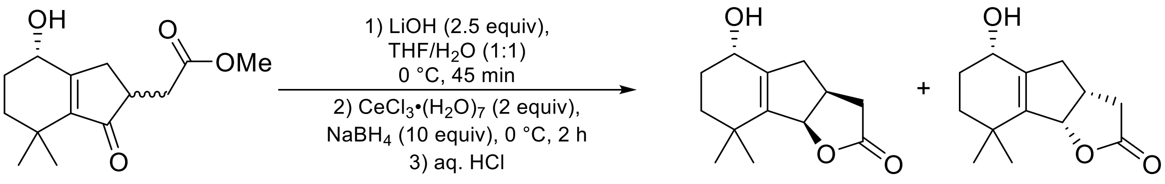

6c **2c,** 35% **2b,** 37%

To a solution of compound **6c** (300 mg, 1.19 mmol, 1.0 equiv) in THF (5 mL), LiOH (71.0 mg, 3.0 mmol, 2.5 equiv) in water (5 mL) was added at 0 °C, and the mixture was stirred for 45 minutes. At 0 °C, $CeCl_3 \cdot 7H_2O$ (894 mg, 2.40 mmol, 2 equiv) was introduced, followed by the addition of a solution of $NaBH_4$ (453 mg, 12.0 mmol, 10 equiv) in cold water (5 mL). The mixture was stirred at 0 °C for 2 hours before the careful addition of a 2 M hydrochloric acid (HCl) solution. The mixture was then extracted with DCM (3 × 20 mL). The combined organic layers were dried over Na_2SO_4 and the solvent was evaporated under reduced pressure. Compound **2b** and **2c** are separable on TLC and silica gel column chromatography. The crude product was purified using silica gel column chromatography (100% EA) to yield product **2b** (98.0 mg, 0.44 mmol, 37% yield). ^{1}H NMR (400 MHz, $CDCl_3$) δ 5.52 – 5.44 (m, 1H), 4.18 (s, 1H), 3.11 – 2.94 (m, 2H), 2.82 (dd, J = 18.2, 10.0 Hz, 1H), 2.35 (dd, J = 18.2, 4.7 Hz, 1H), 2.21 – 2.14 (m, 1H), 2.03 – 1.94 (m, 1H), 1.75 – 1.66 (m, 1H), 1.63 – 1.55 (m, 2H), 1.49 – 1.43 (m, 1H), 1.13 (s, 3H), 1.09 (s, 3H). ^{13}C NMR (101 MHz, $CDCl_3$) δ 177.2, 143.3, 141.9, 89.8, 66.0, 39.1, 36.0, 35.2, 34.6, 32.4, 29.4, 27.3, 27.2. $[\alpha]_D^{26}$ = +4.2 (c = 0.5, $CHCl_3$).

(*Rac*)-*(3aR,8bS)-3,3a,4,8b-tetrahydro-2H-indeno[1,2-b]furan-2-one* ***2d***

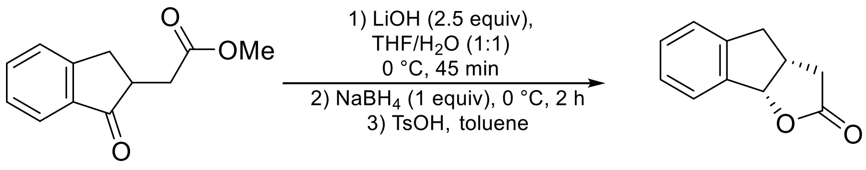

5b (*rac*)**-2d,** 73%

To a solution of compound **5b** (180.0 mg, 0.88 mmol, 1.0 equiv) in THF (3 mL), LiOH (52.8 mg, 2.20 mmol, 2.5 equiv) in water (3 mL) was added at 0 °C, and the mixture was stirred for 60 minutes. THF was evaporated under reduced pressure. The mixture was then added by brine and the aqueous phase was extracted with DCM (3 × 10 mL). Organic solvent was evaporated under

reduced pressure. The residual was added to a NaOH solution (2 mL, 0.2 M), followed by the addition of a solution of $NaBH_4$ (33.3 mg, 0.88 mmol, 1 equiv) in cold water (1 mL). The mixture was stirred at 0 °C for 2 hours. Afterwards, a 2 M HCl (1 mL) was added. The mixture was then extracted with DCM (3 × 20 mL). The combined organic layers were dried over Na_2SO_4 and the solvent was evaporated under reduced pressure. The residual was added to a mixture of TsOH (15.0 mg, 10 mol%) and toluene (10 mL), The mixture was stirred at reflux for 24 hours. Afterwards, 2 M and a saturated Na_2CO_3 (1 mL) was added. The mixture was then extracted with DCM (3 × 20 mL). The combined organic layers were dried over Na_2SO_4 and the solvent was evaporated under reduced pressure. The crude product was purified using silica gel column chromatography (eluent: EA/PE = 1:5) to yield product **2d** (111.6 mg, 0.64 mmol, 73% yield) as a white solid. ^{1}H NMR (400 MHz, $CDCl_3$) δ 7.52 – 7.44 (m, 1H), 7.39 – 7.22 (m, 3H), 5.89 (d, *J* = 7.0 Hz, 1H), 3.49 – 3.26 (m, 2H), 3.06 – 2.78 (m, 2H), 2.52 – 2.31 (m, 1H); ^{13}C NMR (101 MHz, $CDCl_3$) 176.9, 142.5, 138.7, 129.9, 127.5, 126.3, 125.3, 87.6, 37.8, 37.3, 35.6. HRMS (ESI): calc. for $C_{11}H_{10}NaO_2^+$ $[M+Na]^+$: 197.0573, found: 197.0580.

General Procedure for Formylation and O-alkylation with the Bromobutenolide (D-ring)

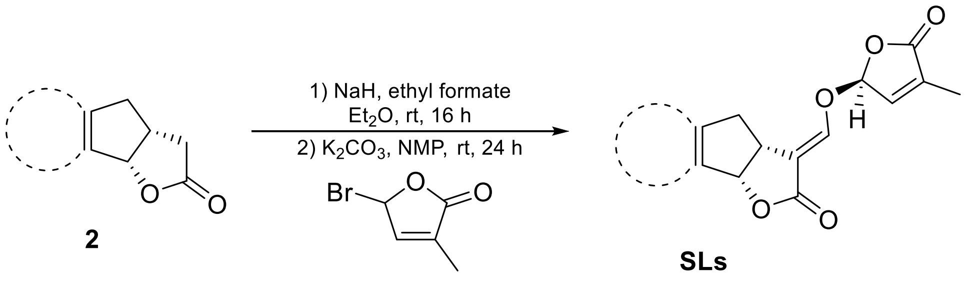

The Formylation and O-alkylation reactions to give SLs or SL analogues from tricyclic lacctones were performed using a reported procedure with minor modifications. Initially, to a stirred solution of NaH (60% in oil, 3 equiv) in Et_2O, a dropwise addition of a solution of tricyclic lactone (0.31 mmol, 1.0 equiv) in Et_2O was carried out, followed by the introduction of ethyl formate (8 - 12 equiv) under a nitrogen atmosphere. After 24 hours, the mixture was acidified with HCl (1M) and extracted with EA. The organic extract was washed with water and brine, dried over Na_2SO_4, and the solvent was removed under reduced pressure to yield the crude product, which was used directly in the subsequent step without further purification. In the next step, to a stirred solution of

hydroxymethylene lactone and K_2CO_3 (2.0 equiv) in dry N-methyl pyrrolidone, bromobutenolide (3 equiv) was added. After stirring for 24 hours, the mixture was poured into HCl (1 M) and extracted with EtOAc. The combined organic extract was washed with water (20 mL) and brine, dried over Na_2SO_4, filtered, and the solvent was evaporated under reduced pressure to yield the crude product. Purification was carried out using chromatography on silica gel.

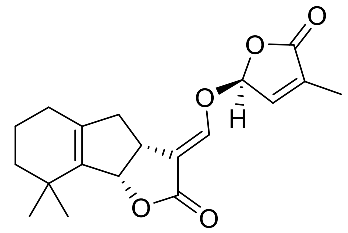

(*rac*)-5-deoxystrigol **1a**

(rac)-5-deoxystrigol **1a**

Eluent: PE/EA = 2/1. Following the general procedure at 0.44 mmol scale, (rac)-5-deoxystrigol (the fast moving diastereomer on TLC, 61.0 mg, 0.184 mmol, 42% yield) was obtained as a white solid. ^{1}H NMR (400 MHz, $CDCl_3$) δ 7.41 (d, *J* = 2.5 Hz, 1H), 6.93 – 6.89 (m, 1H), 6.19 – 6.13 (m, 1H), 5.51 (d, *J* = 7.8 Hz, 1H), 3.63 – 3.52 (m, 1H), 2.70 (dd, *J* = 16.8, 9.2 Hz, 1H), 2.43 – 2.27 (m, 1H), 2.02 (t, *J* = 1.5 Hz, 3H), 1.98 – 1.86 (m, 2H), 1.71 – 1.66 (m, 1H), 1.66 – 1.61 (m, 1H), 1.51 – 1.44 (m, 1H), 1.39 – 1.33 (m, 1H), 1.11 (s, 3H), 1.09 (s, 3H). ^{13}C NMR (101 MHz, $CDCl_3$) δ 171.7, 170.2, 149.7, 141.6, 141.0, 139.7, 136.0, 114.4, 100.4, 88.4, 41.3, 39.0, 36.6, 31.9, 28.1, 27.8, 26.4, 19.2, 10.8. HRMS (ESI): calc. for $C_{19}H_{22}NaO_5^+$ $[M+Na]^+$: 353.1359, found: 353.1361. These spectra data were in good accord with those reported in literature.[9]

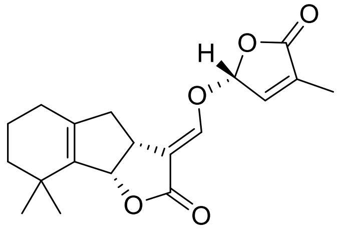

(*rac*)-(*epi*)-5-deoxystrigol **1b**

(rac)(epi)-5-deoxystrigol **1b**

Eluent: PE/EA = 2/1 to 1/1. Following the general procedure at 0.44 mmol scale, (rac) (epi)-5-deoxystrigol (the slow moving diastereomer on TLC, 55.1 mg, 0.167 mmol, 38% yield) was obtained as a white solid. ^{1}H NMR (400 MHz, $CDCl_3$) δ 7.42 (d, *J* = 2.5 Hz, 1H), 7.00 – 6.86 (m,

1H), 6.18 – 6.08 (m, 1H), 5.52 (d, *J* = 7.8 Hz, 1H), 3.63 – 3.52 (m, 1H), 2.68 (dd, *J* = 17.0, 9.1 Hz, 1H), 2.37 – 2.23 (m, 1H), 2.02 (t, *J* = 1.5 Hz, 3H), 1.99 – 1.85 (m, 2H), 1.74 – 1.66 (m, 1H), 1.64 – 1.58 (m, 1H), 1.50 – 1.43 (m, 1H), 1.39 – 1.31 (m, 1H), 1.11 (s, 3H), 1.09 (s, 3H). ^{13}C NMR (101 MHz, $CDCl_3$) δ 171.8, 170.3, 150.1, 141.8, 141.0, 139.5,135.8, 114.4, 100.6, 88.5, 41.3, 39.0, 36.5, 31.9, 28.1, 27.8, 26.3, 19.2, 10.7. HRMS (ESI): calc. for $C_{19}H_{22}NaO_5^+$ $[M+Na]^+$: 353.1359, found: 353.1356. These spectra were in good accord with those reported in literature. [9]

(+)-*strigol* **1c**

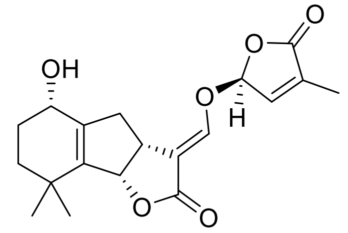

(+)-strigol **1c**

Following the general procedure at 0.31 mmol scale. Eluent: DCM to EtOAc/CH_2Cl_2 3:7) to give the product Strigol (the fast moving diastereomer on TLC, 25.0 mg, 0.072 mmol, 23% yield for 2 steps,) as a white solid. ^{1}H NMR (400 MHz, $CDCl_3$) δ 7.44 (d, *J* = 2.6 Hz, 1H), 6.96 – 6.89 (m, 1H), 6.18 – 6.12 (m, 1H), 5.55 – 5.47 (m, 1H), 4.14 – 4.05 (m, 1H), 3.64 (tdd, *J* = 8.1, 4.3, 2.6 Hz, 1H), 2.74 – 2.65 (m, 2H), 2.02 (t, *J* = 1.6 Hz, 3H), 2.02 – 1.91 (m, 1H), 1.75 – 1.63 (m, 1H), 1.61 – 1.51 (m, 1H), 1.44 (ddd, *J* = 13.9, 11.4, 3.0 Hz, 1H), 1.16 (s, 3H), 1.09 (s, 3H). ^{13}C NMR (101 MHz, $CDCl_3$) δ 171.5, 170.2, 150.2, 142.7, 142.3, 141.0, 136.0, 113.8, 100.4, 87.9, 67.3, 37.9, 37.0, 36.6, 32.4, 29.7, 27.6, 27.5, 10.8. $[\alpha]_D^{26}$ = +263.0 (c = 0.3, $CHCl_3$). These spectra were in good accord with those reported in literature.[8]

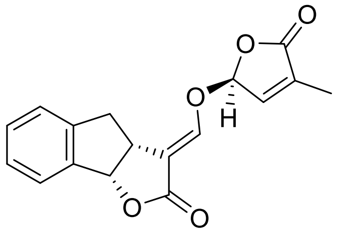

(*rac*)-GR-24 **1d**

(*rac*)-*GR-24* **1d**

Eluent: PE/EA = 2/1. Following the general procedure at 0.4 mmol scale, (rac)-GR-24 (the fast moving diastereomer on TLC, 42.9 mg, 0.144 mmol, 36% yield) was obtained as a slightly yellow solid. ^{1}H NMR (400 MHz, $CDCl_3$) δ 7.56 – 7.43 (m, 2H), 7.38 – 7.27 (m, 2H), 7.25 – 7.15 (m, 1H), 7.04 – 6.92 (m, 1H), 6.23 – 6.12 (m, 1H), 5.95 (d, *J* = 7.8 Hz, 1H), 3.94 (t, *J* = 8.4 Hz, 1H), 3.43 (dd, *J* = 16.9, 9.3 Hz, 1H), 3.16 – 3.04 (m, 1H), 2.03 (s, 3H). ^{13}C NMR (101 MHz, $CDCl_3$) δ 171.3, 170.1, 150.9, 142.5, 140.8, 138.8, 136.1, 130.0, 127.5, 126.5, 125.1, 113.3, 100.5, 85.9, 38.8, 37.3, 10.8. HRMS (ESI): calc. for $C_{17}H_{14}NaO_5^+$ $[M+Na]^+$: 321.0733, found: 321.0731. These spectra were in good accord with those reported in literature.[10]

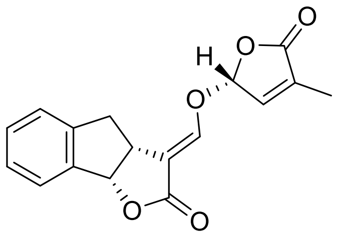

(*rac*)-(*epi*)-GR-24 **1e**

(*rac*)-(*epi*)-*GR-24* **1e**

Eluent: PE/EA = 2/1 to 1/1. Following the general procedure at 0.4 mmol scale, (*rac, epi*)-GR-24 (the slow moving diastereomer on TLC, 39.7 mg, 0.133 mmol, 33% yield) was obtained as a slightly yellow solid. ^{1}H NMR (400 MHz, $CDCl_3$) δ 7.63 – 7.44 (m, 2H), 7.37 – 7.31 (m, 2H), 7.25 – 7.21 (m, 1H), 7.01 – 6.93 (m, 1H), 6.20 – 6.16 (m, 1H) 5.96 (d, *J* = 7.9 Hz, 1H), 4.05 – 3.87 (m, 1H), 3.42 (dd, *J* = 16.9, 9.3 Hz, 1H), 3.10 (dd, *J* = 16.9, 3.4 Hz, 1H), 2.04 (s, 3H). ^{13}C NMR (101 MHz, $CDCl_3$) δ 171.3, 170.2, 151.0, 142.6, 140.9, 138.7, 136.0, 130.0, 127.5, 126.4, 125.2, 113.4, 100.6, 85.9, 38.8, 37.4, 10.8. HRMS (ESI): calc. for $C_{17}H_{14}NaO_5^+$ $[M+Na]^+$: 321.0733, found: 321.0734. These spectra were in good accord with those reported in literature.[10]

5. Copies of Spectra

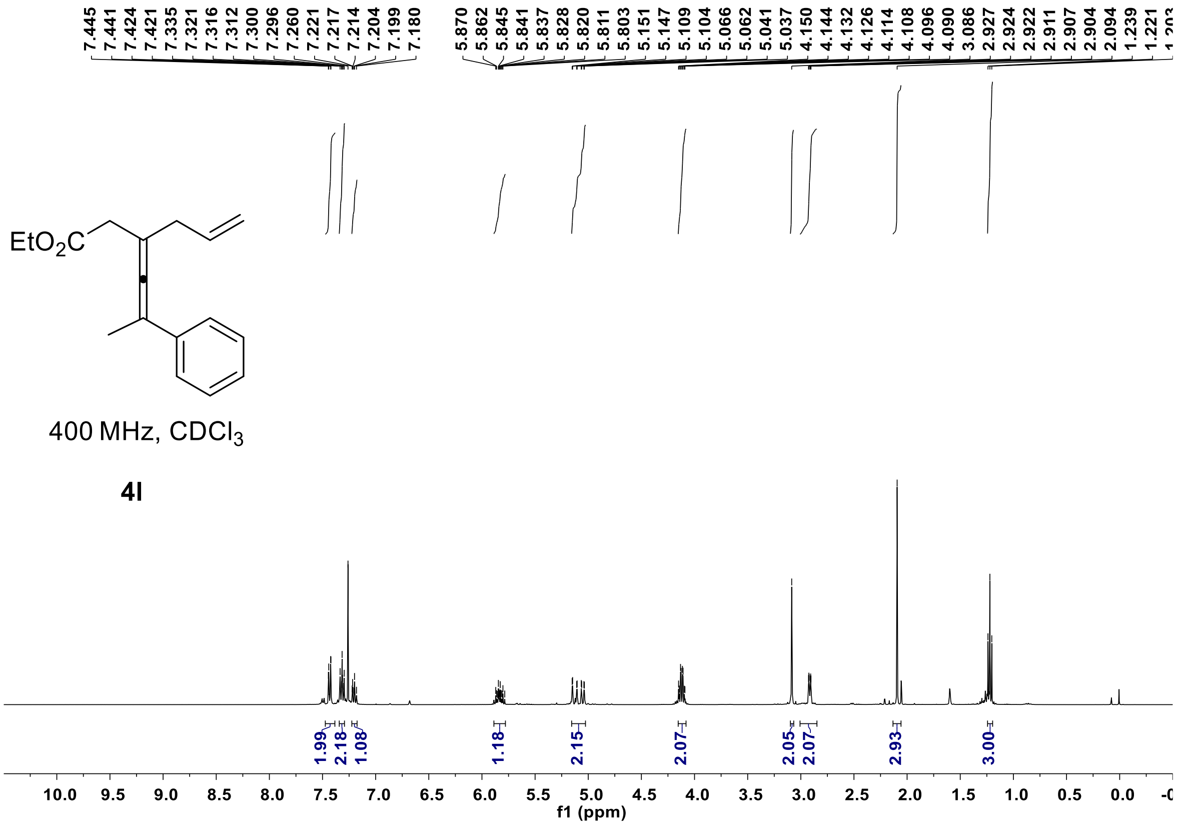

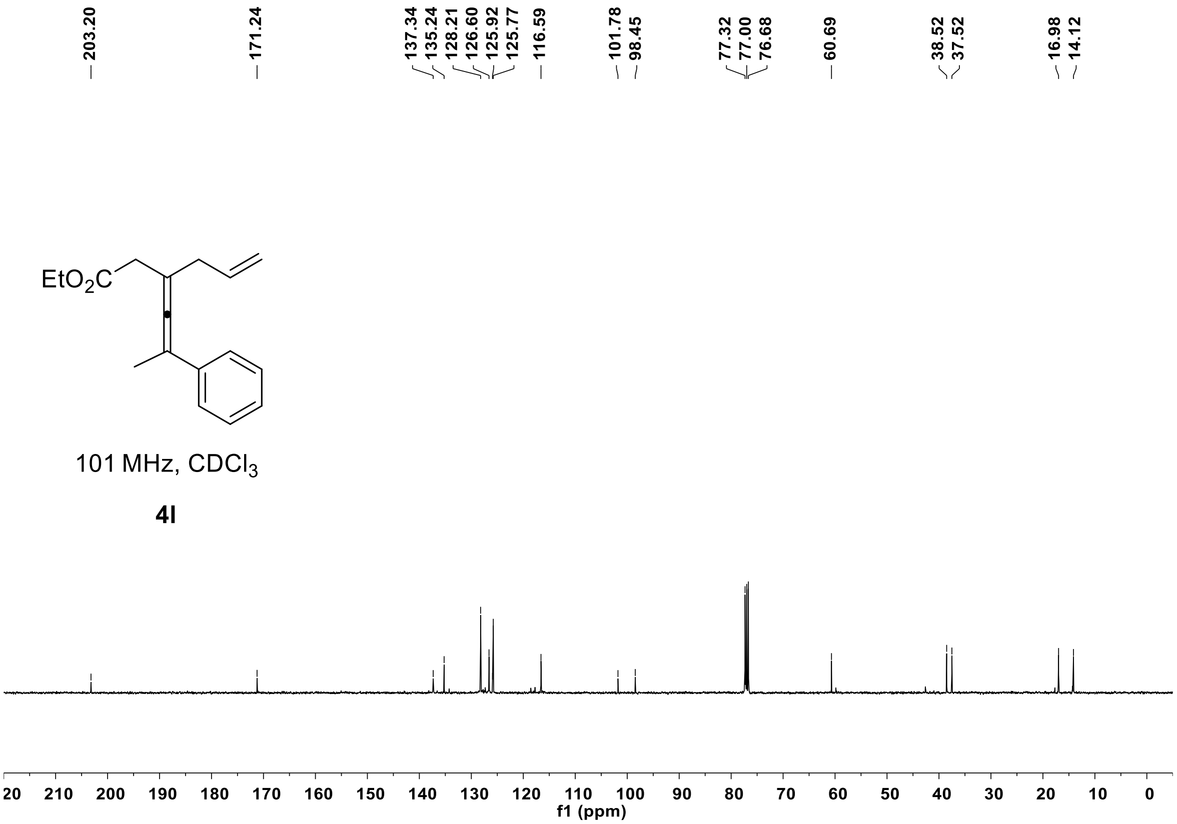

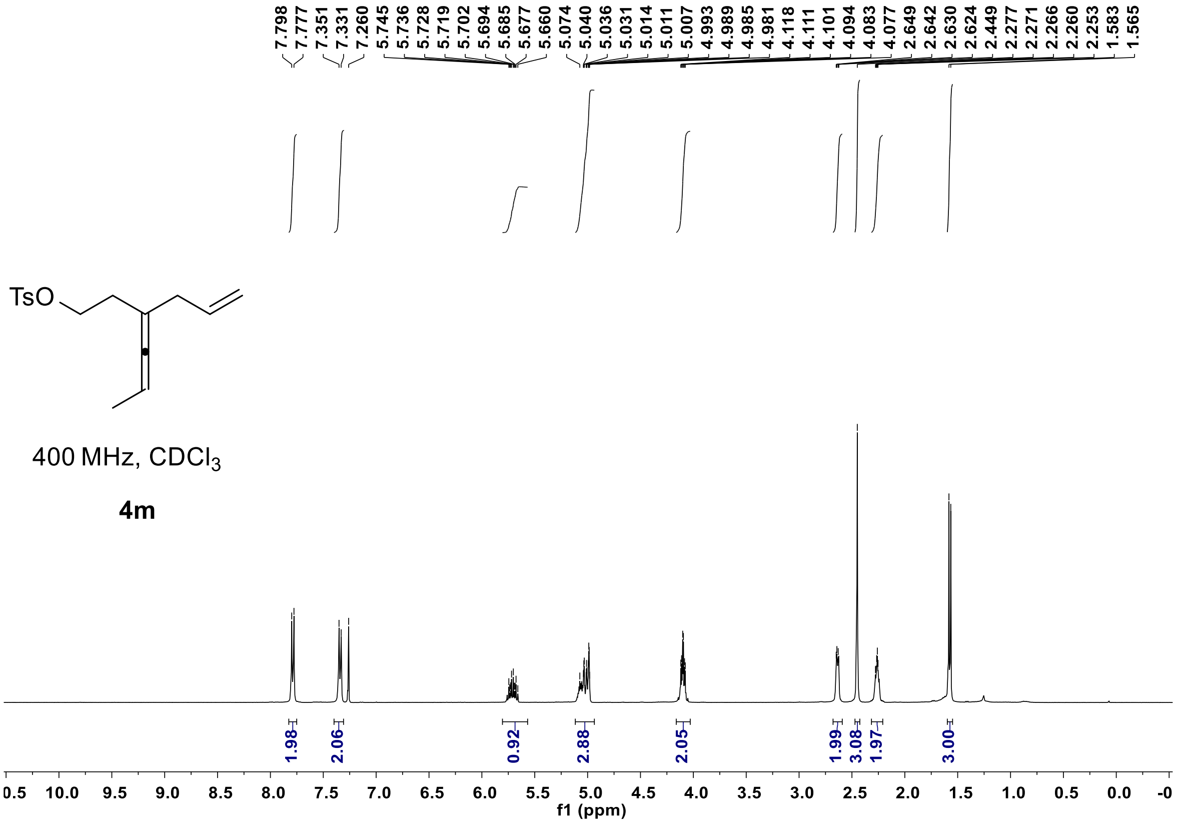
7.798
7.777
7.351
7.331
7.260
5.745
5.736
5.728
5.719
5.702
5.694
5.685
5.677
5.660
5.074
5.040
5.036
5.031
5.014
5.011
5.007
4.993
4.989
4.985
4.981
4.118
4.111
4.101
4.094
4.083
4.077
2.649
2.642
2.630
2.624
2.449
2.277
2.271
2.266
2.260
2.253
1.583
1.565
TsO
400 MHz, $CDCl_3$
4m
1.98
2.06
0.92
2.88
2.05
1.99
3.08
1.97
3.00
f1 (ppm)

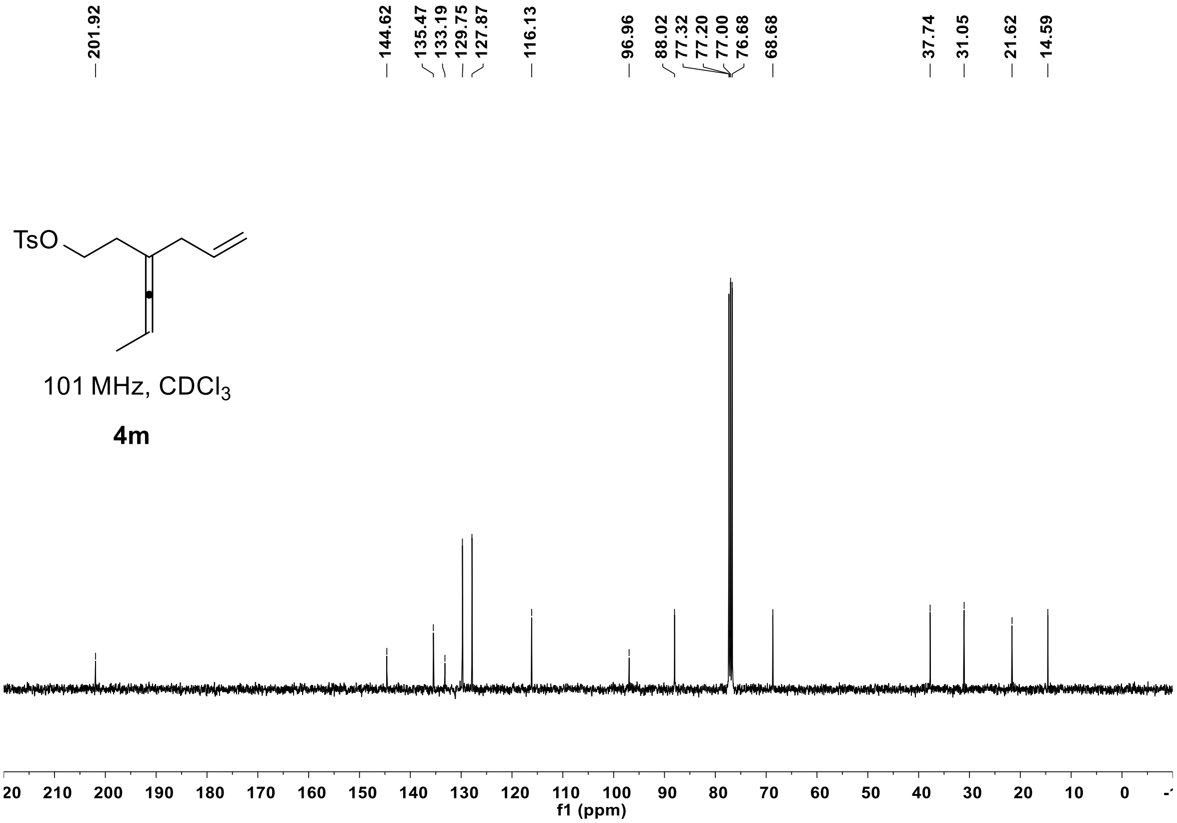
201.92
144.62
135.47
133.19
129.75
127.87
116.13
96.96
88.02
77.32
77.20
77.00
76.68
68.68
37.74
31.05
21.62
14.59
TsO
101 MHz, $CDCl_3$
4m
f1 (ppm)

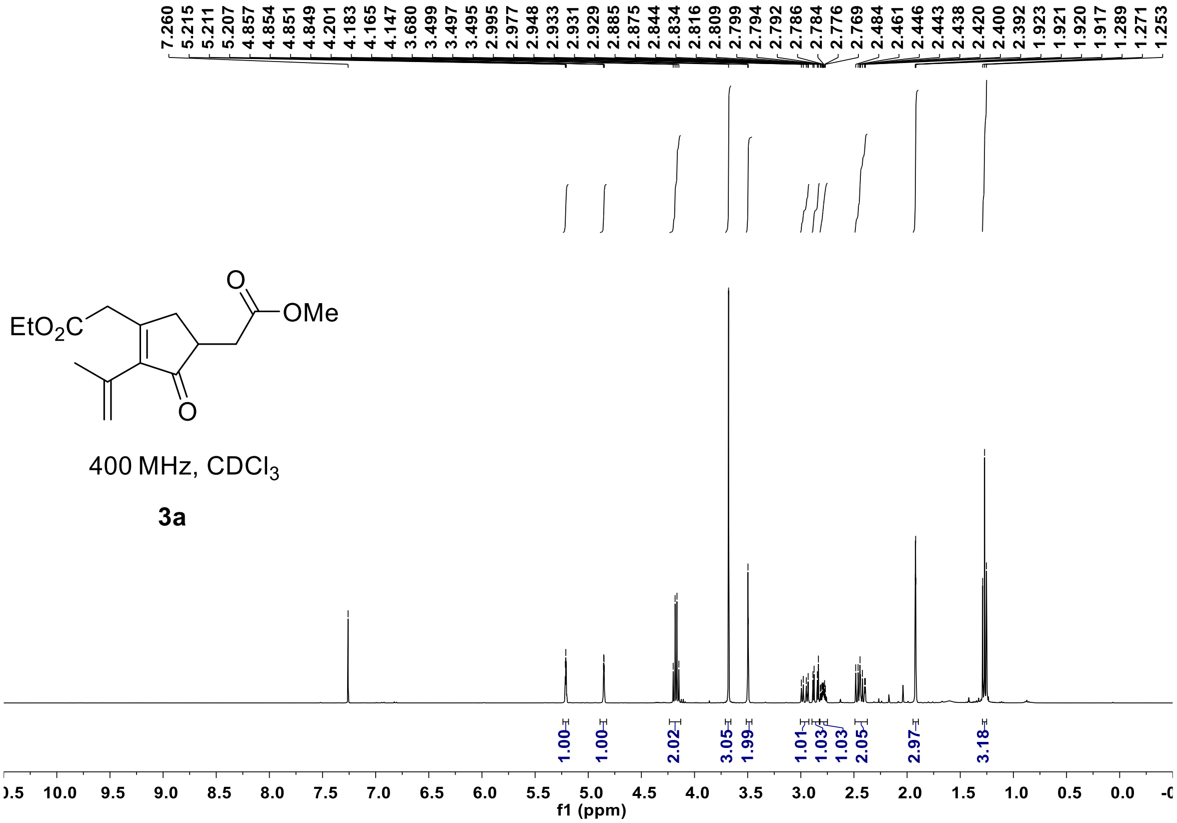
7.260 5.215 5.211 5.207 4.857 4.854 4.851 4.849 4.201 4.183 4.165 4.147 3.680 3.499 3.497 3.495 2.995 2.977 2.948 2.933 2.931 2.929 2.885 2.875 2.844 2.834 2.816 2.809 2.799 2.794 2.792 2.786 2.784 2.776 2.769 2.484 2.461 2.446 2.443 2.438 2.420 2.400 2.392 1.923 1.921 1.920 1.917 1.289 1.271 1.253
EtO2C
OMe
O
400 MHz, CDCl3
3a
1.00 1.00 2.02 3.05 1.99 1.01 1.03 1.03 2.05 2.97 3.18
f1 (ppm)

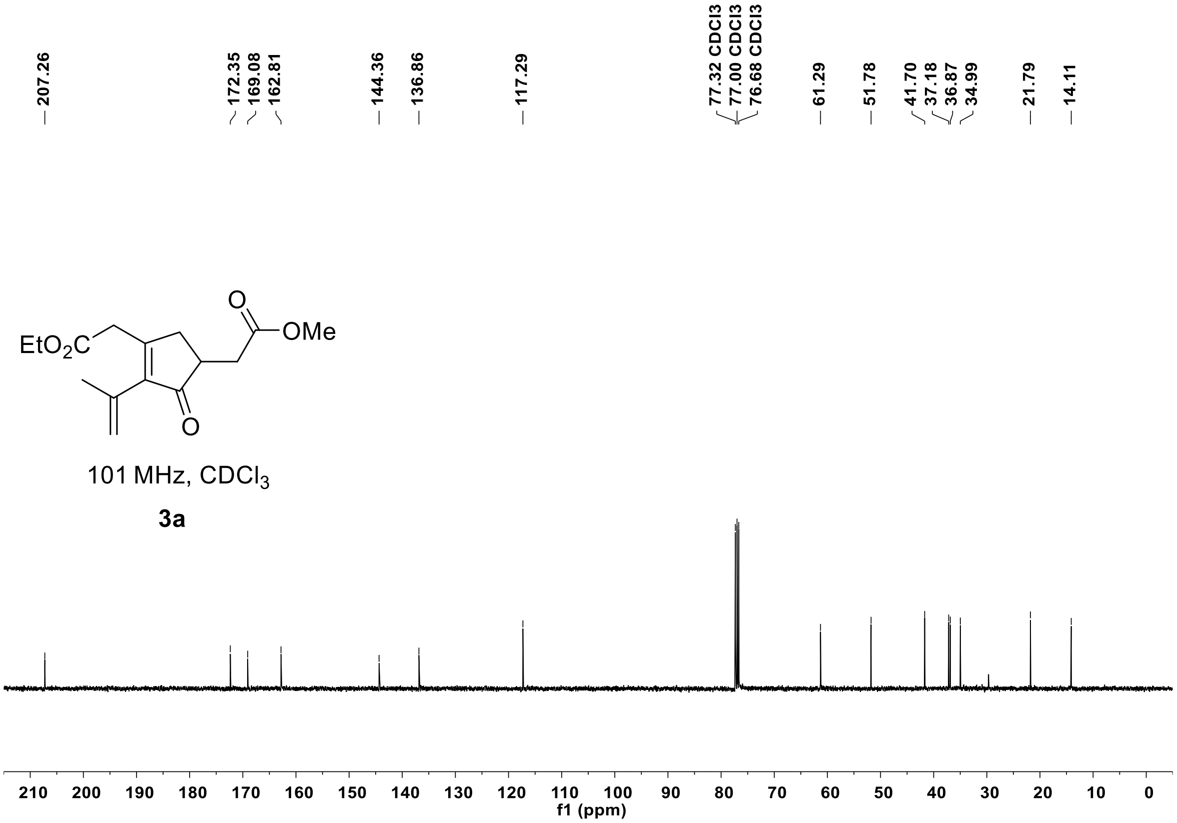
207.26 172.35 169.08 162.81 144.36 136.86 117.29 77.32 CDCl3 77.00 CDCl3 76.68 CDCl3 61.29 51.78 41.70 37.18 36.87 34.99 21.79 14.11
EtO2C
OMe
O
101 MHz, CDCl3
3a
f1 (ppm)

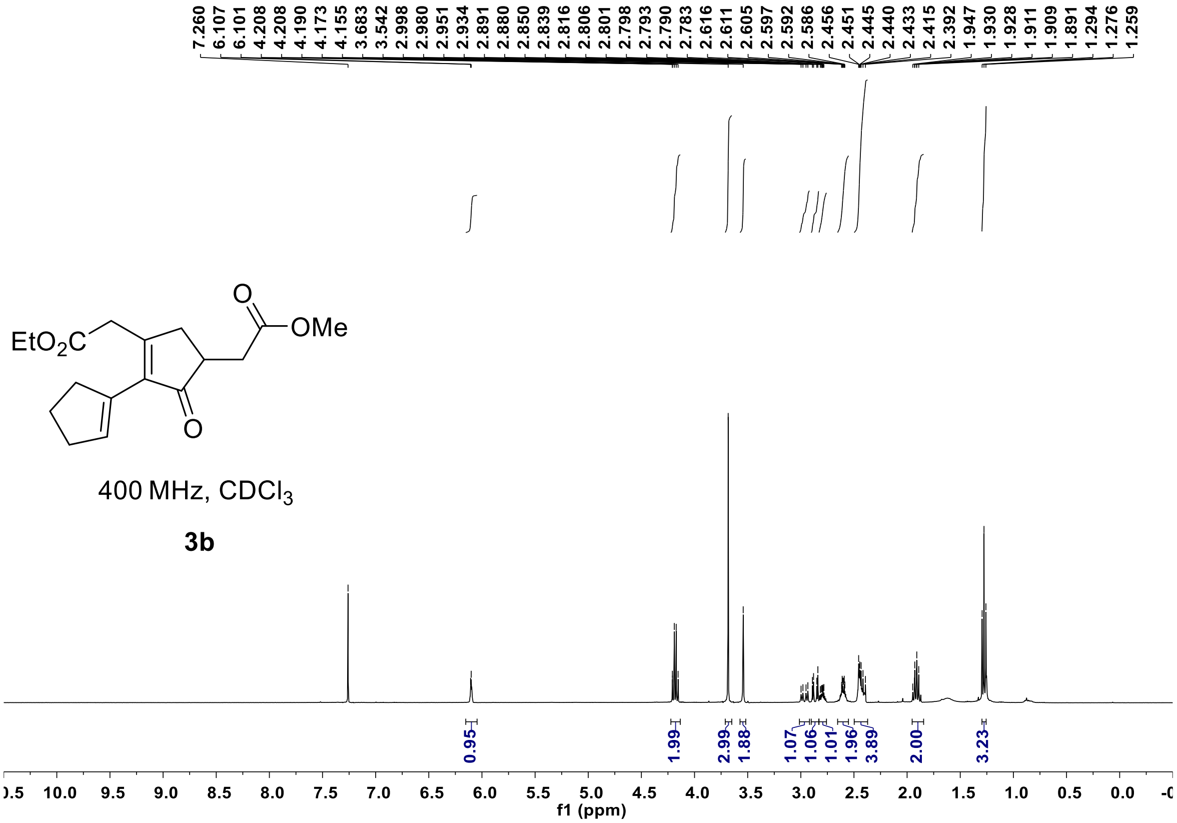
EtO2C
OMe
400 MHz, CDCl3
3b
f1 (ppm)

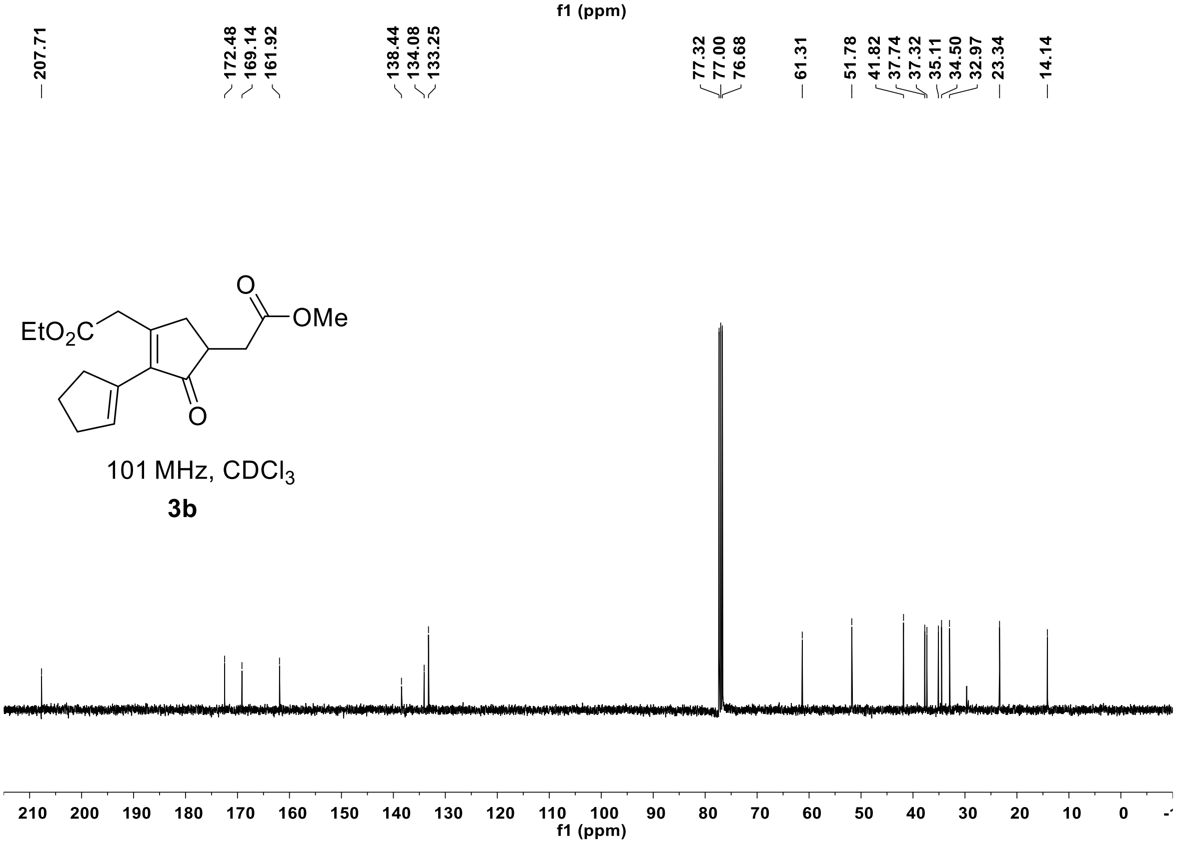
EtO2C
OMe
101 MHz, CDCl3
3b
f1 (ppm)

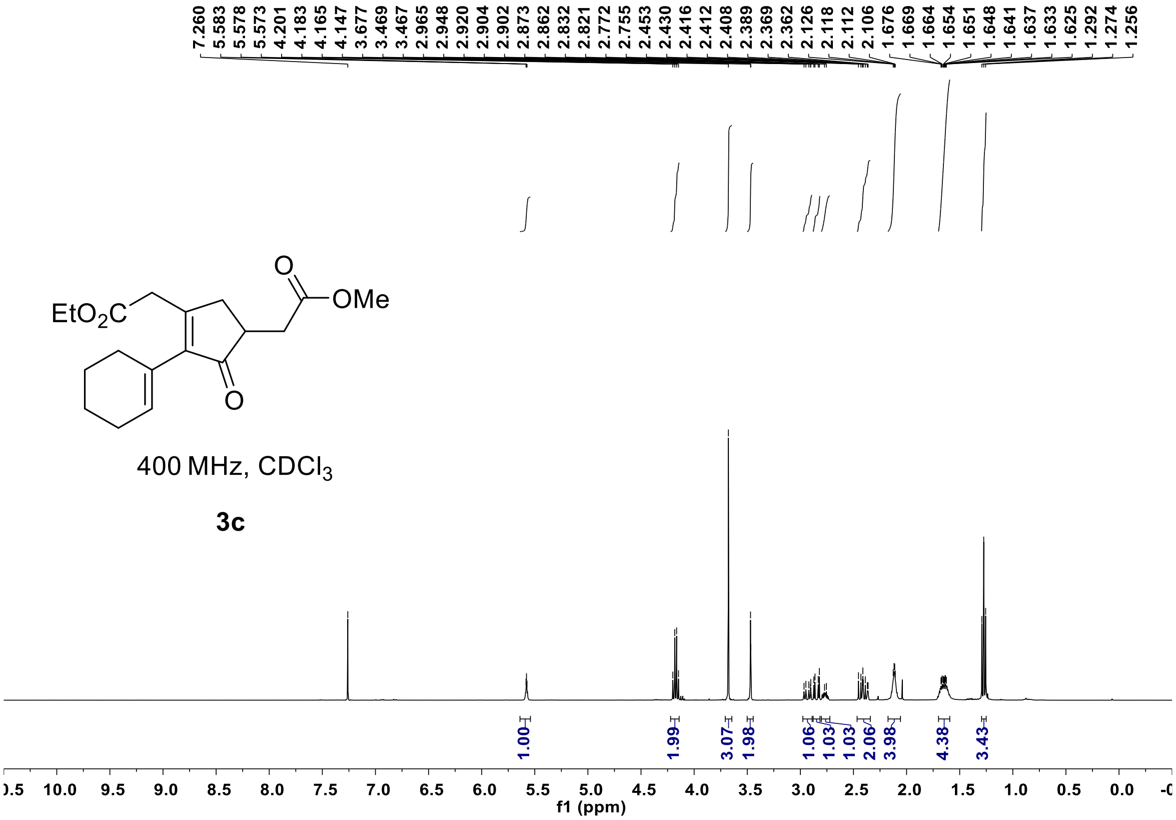
7.260 5.583 5.578 5.573 4.201 4.183 4.165 4.147 3.677 3.469 3.467 2.965 2.948 2.920 2.904 2.902 2.873 2.862 2.832 2.821 2.772 2.755 2.453 2.430 2.416 2.412 2.408 2.389 2.369 2.362 2.126 2.118 2.112 2.106 1.676 1.669 1.664 1.654 1.651 1.648 1.641 1.637 1.633 1.625 1.292 1.274 1.256
EtO2C
OMe
O
O
400 MHz, CDCl3
3c
1.00 1.99 3.07 1.98 1.06 1.03 1.03 2.06 3.98 4.38 3.43
f1 (ppm)

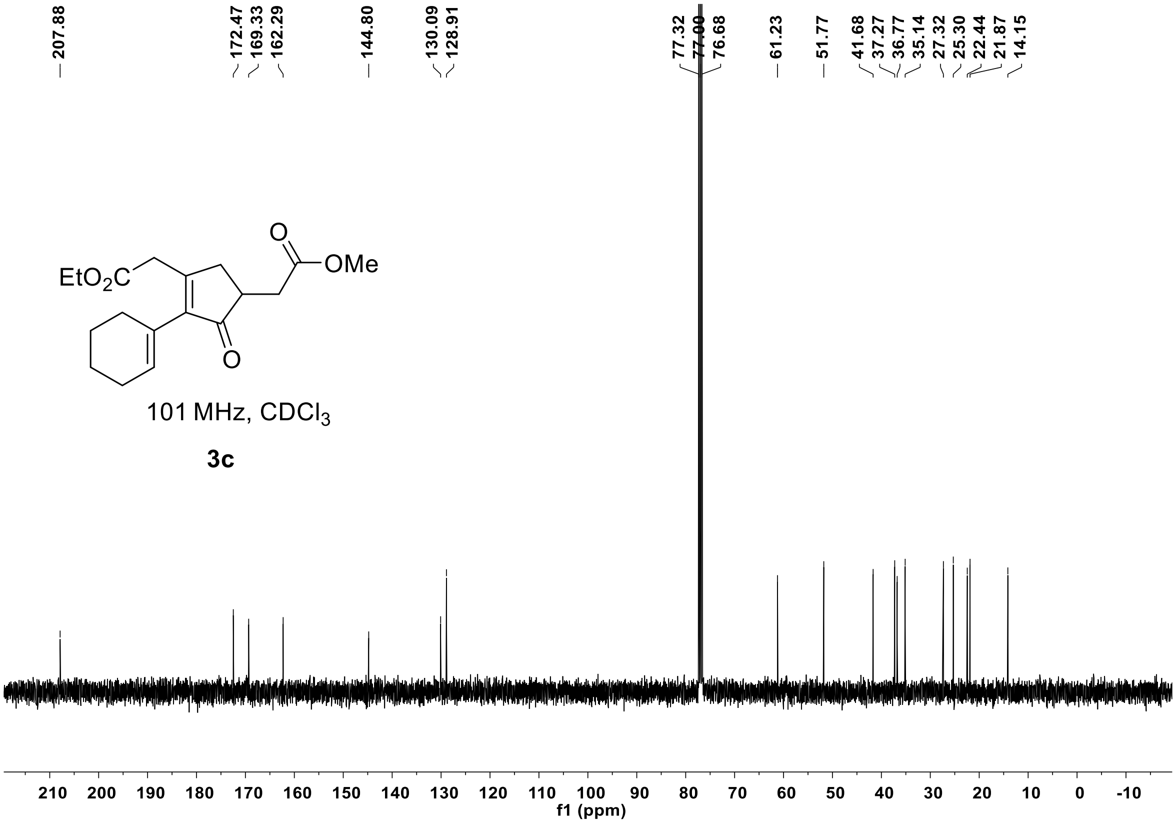
207.88 172.47 169.33 162.29 144.80 130.09 128.91 77.32 77.00 76.68 61.23 51.77 41.68 37.27 36.77 35.14 27.32 25.30 22.44 21.87 14.15
EtO2C
OMe
O
O
101 MHz, CDCl3
3c
f1 (ppm)

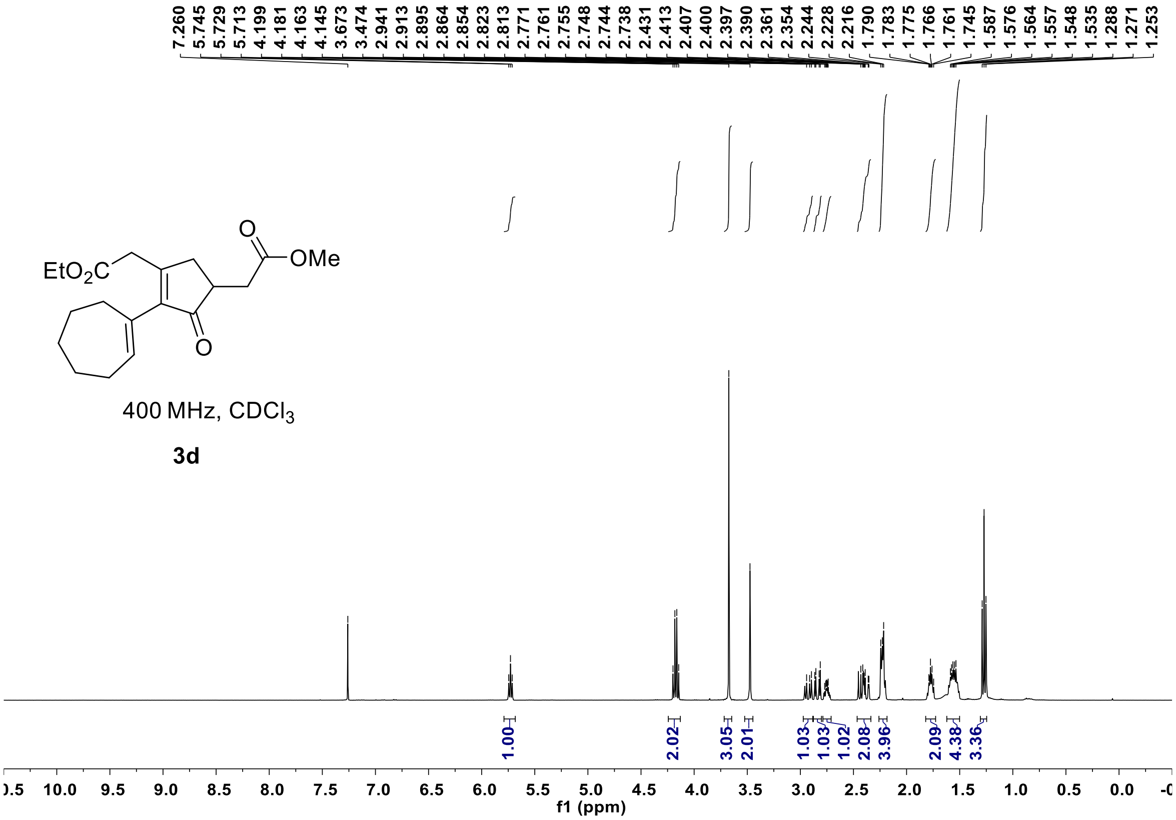
7.260 5.745 5.729 5.713 4.199 4.181 4.163 4.145 3.673 3.474 2.941 2.913 2.895 2.864 2.854 2.823 2.813 2.771 2.761 2.755 2.748 2.744 2.738 2.431 2.413 2.407 2.400 2.397 2.390 2.361 2.354 2.244 2.228 2.216 1.790 1.783 1.775 1.766 1.761 1.745 1.587 1.576 1.564 1.557 1.548 1.535 1.288 1.271 1.253
EtO2C
OMe
O
O
400 MHz, CDCl3
3d
1.00 2.02 3.05 2.01 1.03 1.03 1.02 2.08 3.96 2.09 4.38 3.36
f1 (ppm)

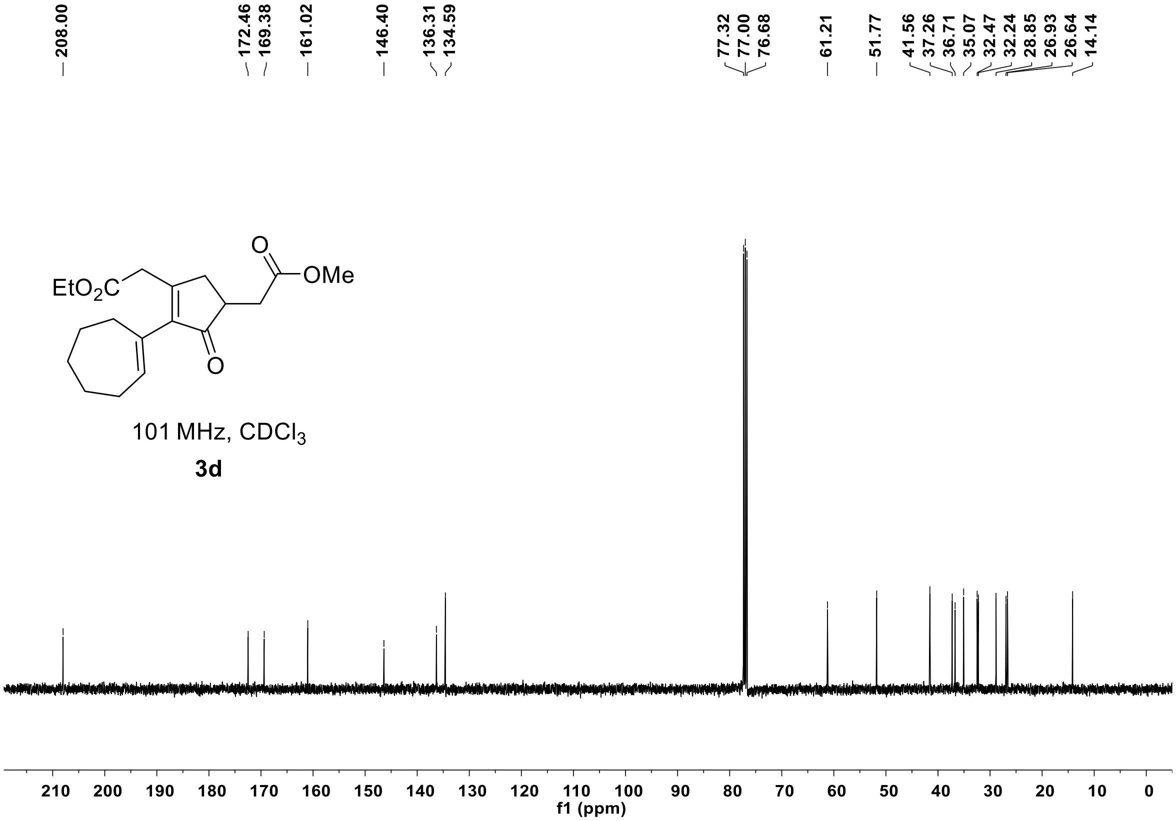
208.00 172.46 169.38 161.02 146.40 136.31 134.59 77.32 77.00 76.68 61.21 51.77 41.56 37.26 36.71 35.07 32.47 32.24 28.85 26.93 26.64 14.14
EtO2C
OMe
O
O
101 MHz, CDCl3
3d
f1 (ppm)

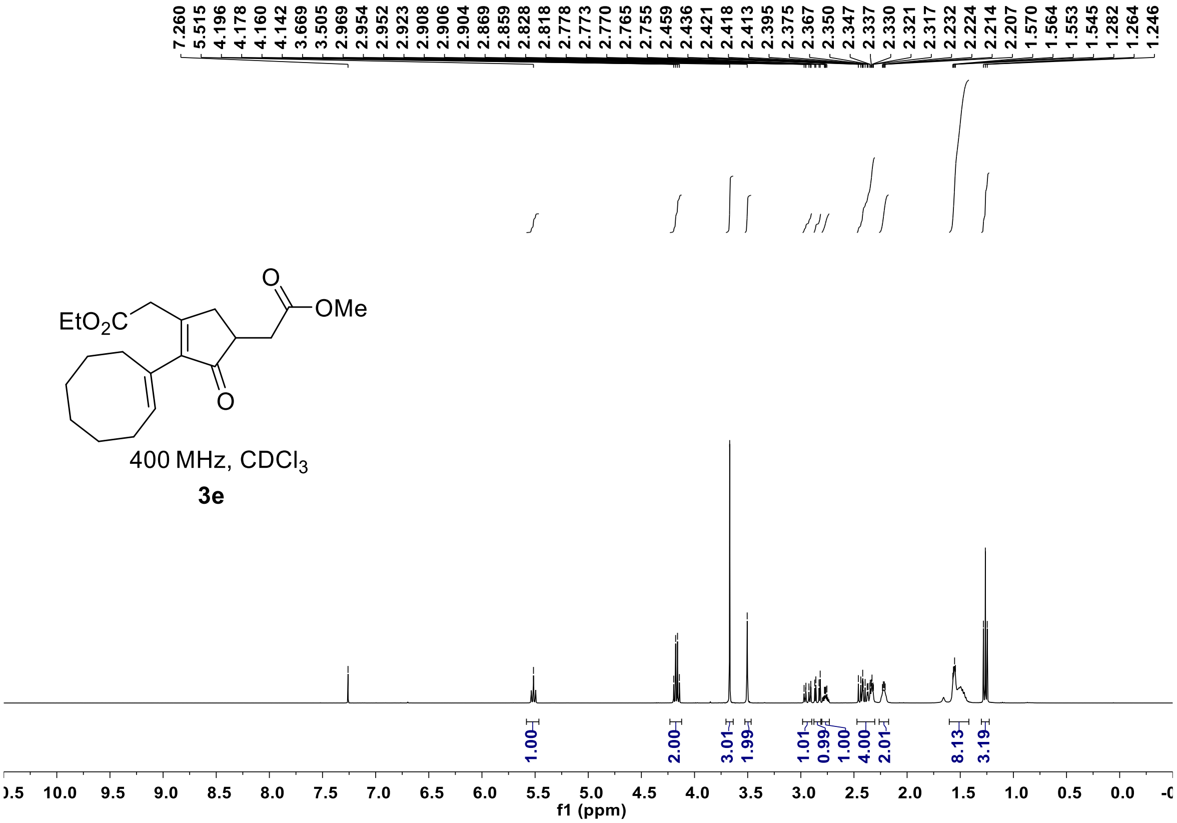

400 MHz, CDCl3
3e

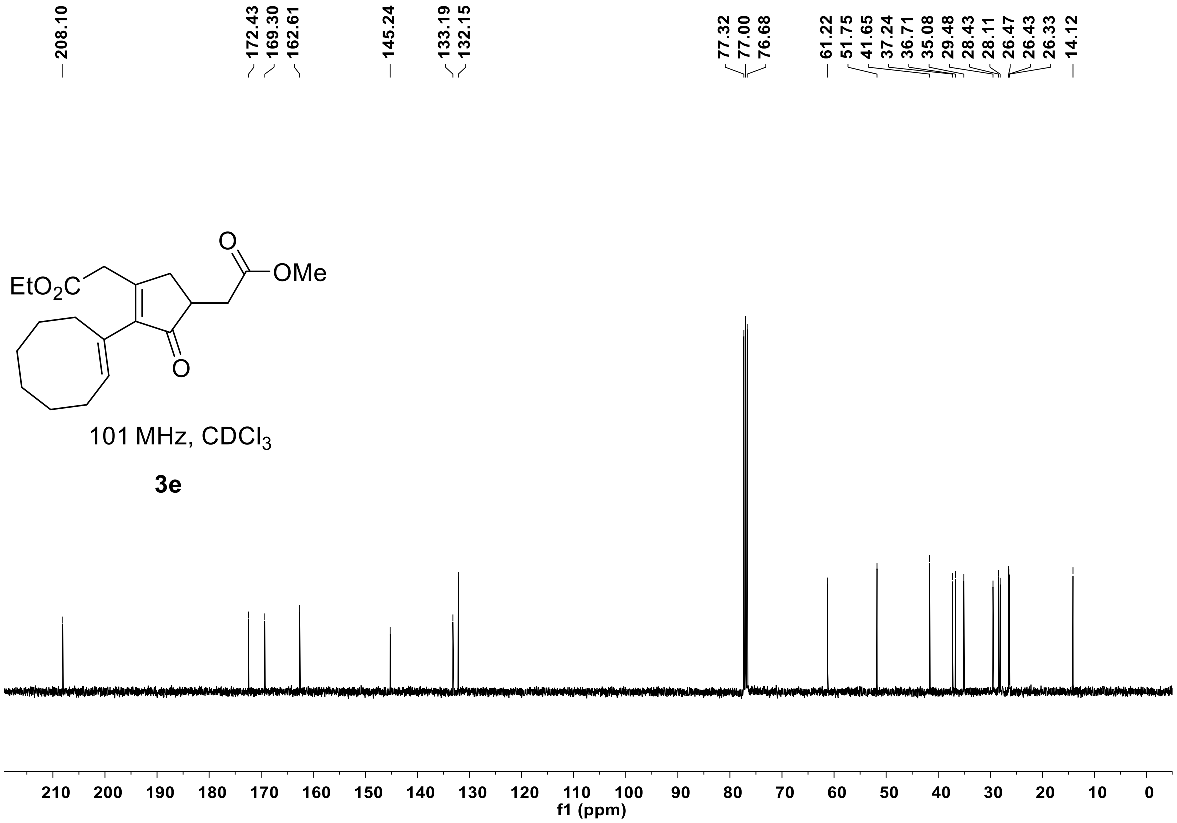

101 MHz, CDCl3
3e

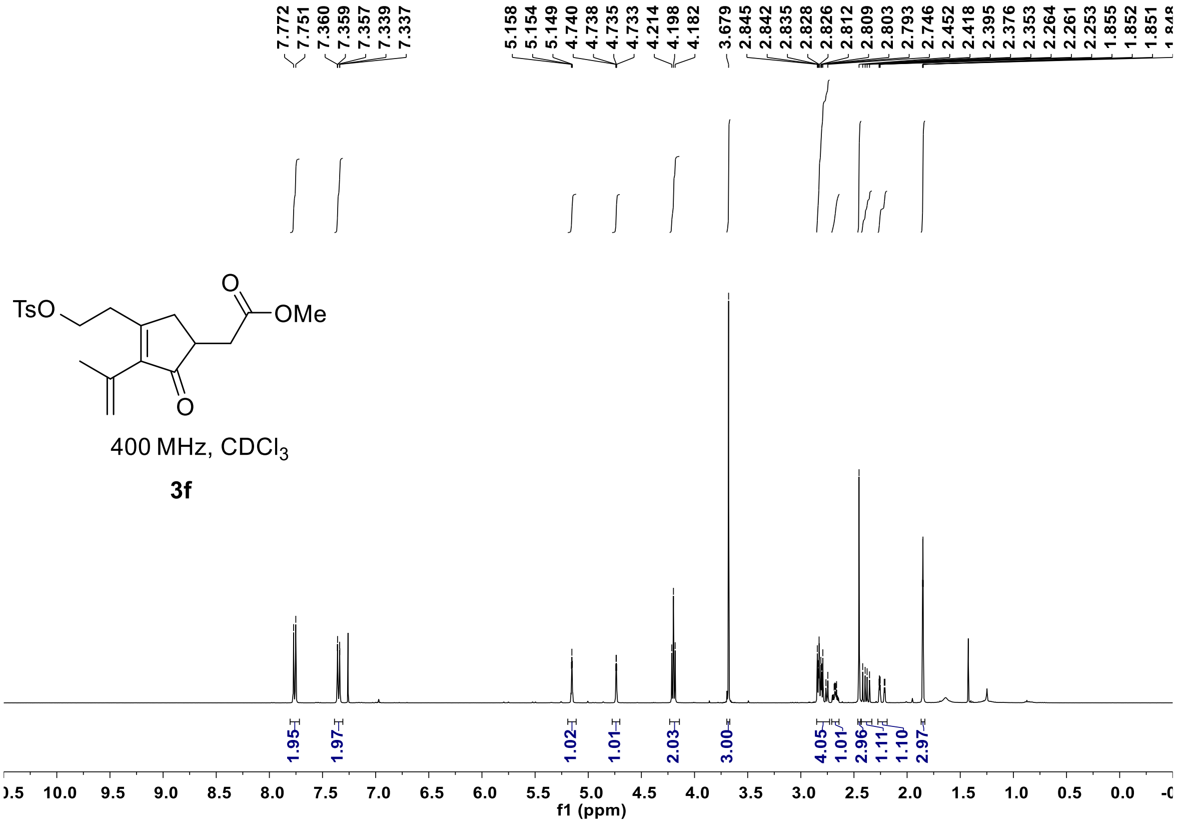

TsO
OMe
O
O
400 MHz, CDCl3
3f
7.772
7.751
7.360
7.359
7.357
7.339
7.337
5.158
5.154
5.149
4.740
4.738
4.735
4.733
4.214
4.198
4.182
3.679
1.95
1.97
1.02
1.01
2.03
3.00
4.05
1.01
2.96
1.11
1.10
2.97
f1 (ppm)

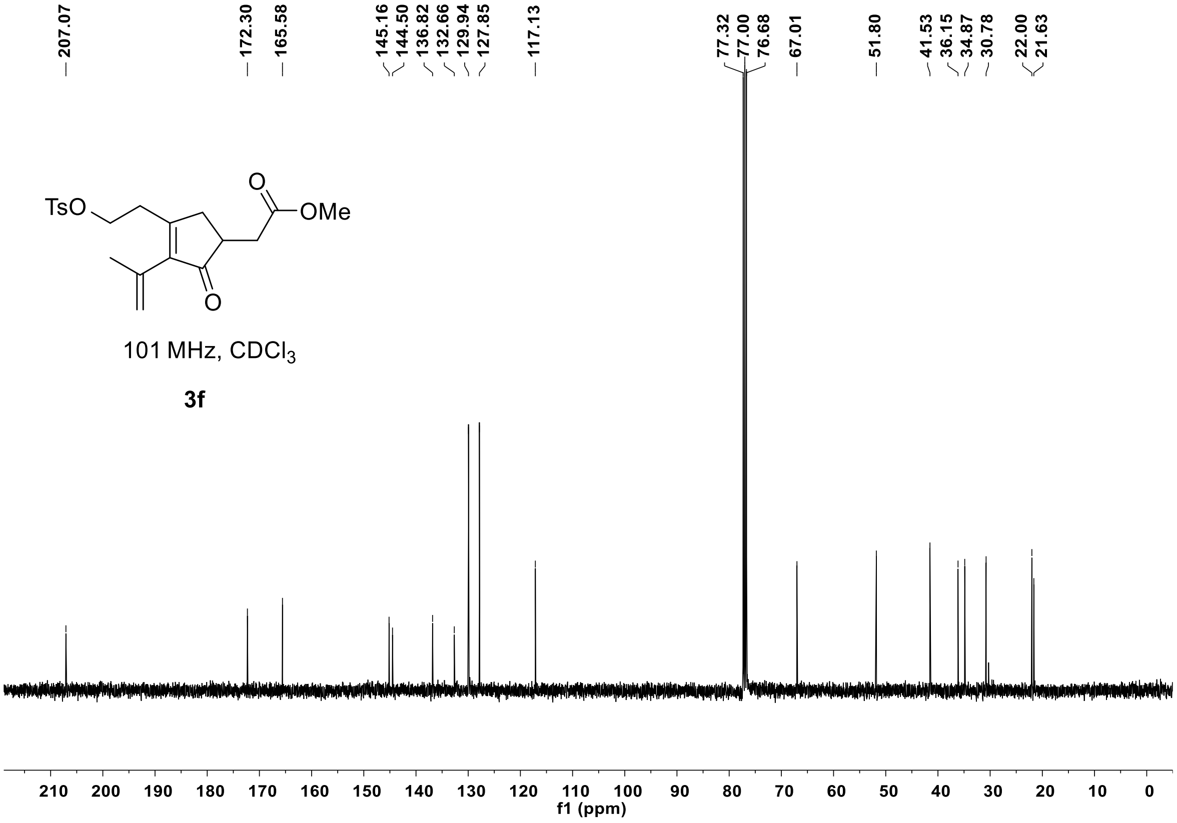

207.07
172.30
165.58
145.16
144.50
136.82
132.66
129.94
127.85
117.13
77.32
77.00
76.68
67.01
51.80
41.53
36.15
34.87
30.78
22.00
21.63
TsO
OMe
O
O
101 MHz, CDCl3
3f
f1 (ppm)

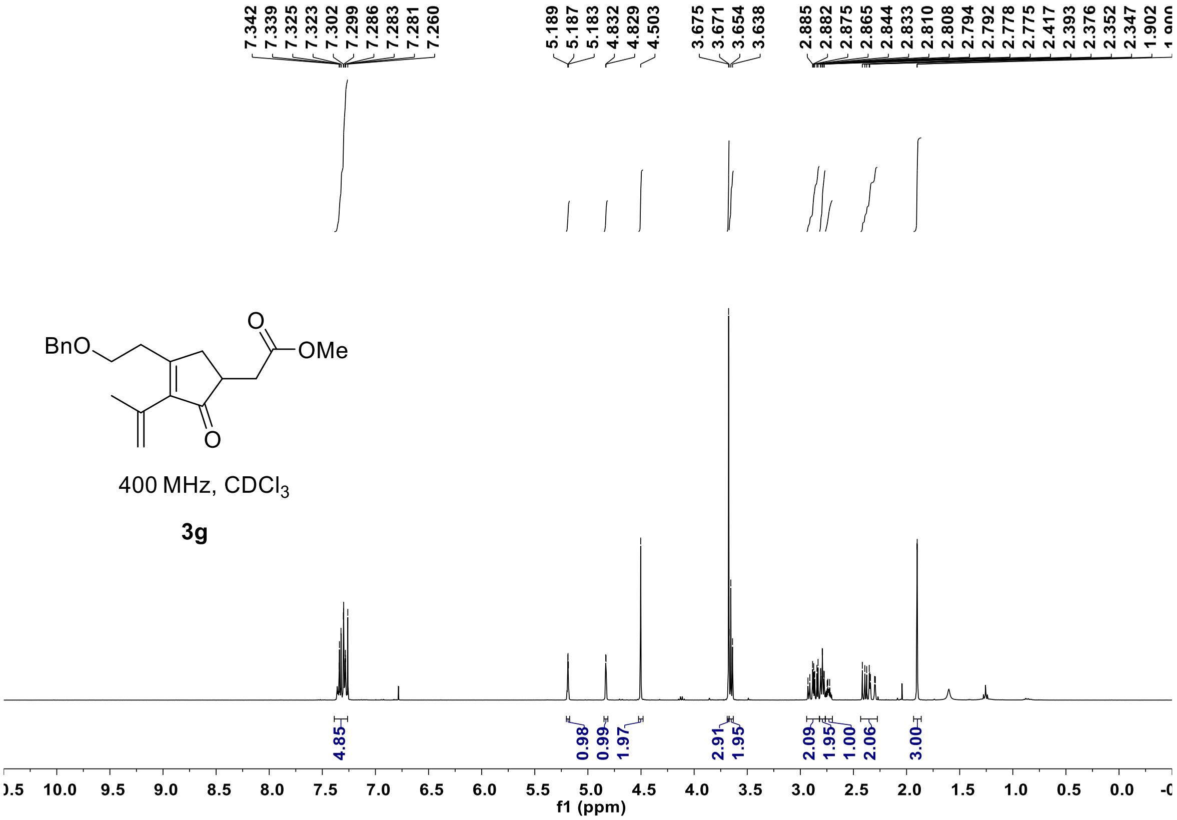
7.342
7.339
7.325
7.323
7.302
7.299
7.286
7.283
7.281
7.260
5.189
5.187
5.183
4.832
4.829
4.503
3.675
3.671
3.654
3.638
2.885
2.882
2.875
2.865
2.844
2.833
2.810
2.808
2.794
2.792
2.778
2.775
2.417
2.393
2.376
2.352
2.347
1.902
BnO
O
OMe
O
400 MHz, CDCl3
3g
4.85
0.98
0.99
1.97
2.91
1.95
2.09
1.95
1.00
2.06
3.00
10.0
9.5
9.0
8.5
8.0
7.5
7.0
6.5
6.0
5.5
5.0
4.5
4.0
3.5
3.0
2.5
2.0
1.5
1.0
0.5
0.0
f1 (ppm)

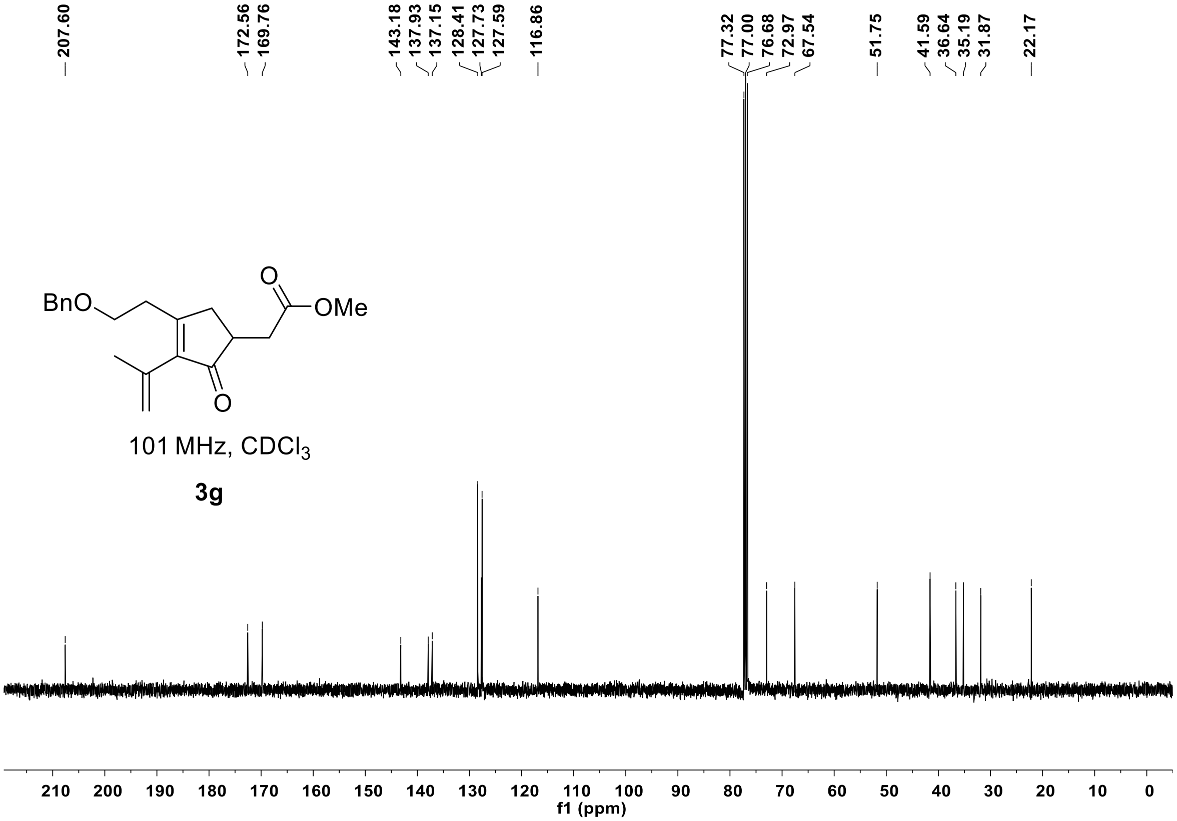
207.60
172.56
169.76
143.18
137.93
137.15
128.41
127.73
127.59
116.86
77.32
77.00
76.68
72.97
67.54
51.75
41.59
36.64
35.19
31.87
22.17
BnO
O
OMe
O
101 MHz, CDCl3
3g
210
200
190
180
170
160
150
140
130
120
110
100
90
80
70
60
50
40
30
20
10
0
f1 (ppm)

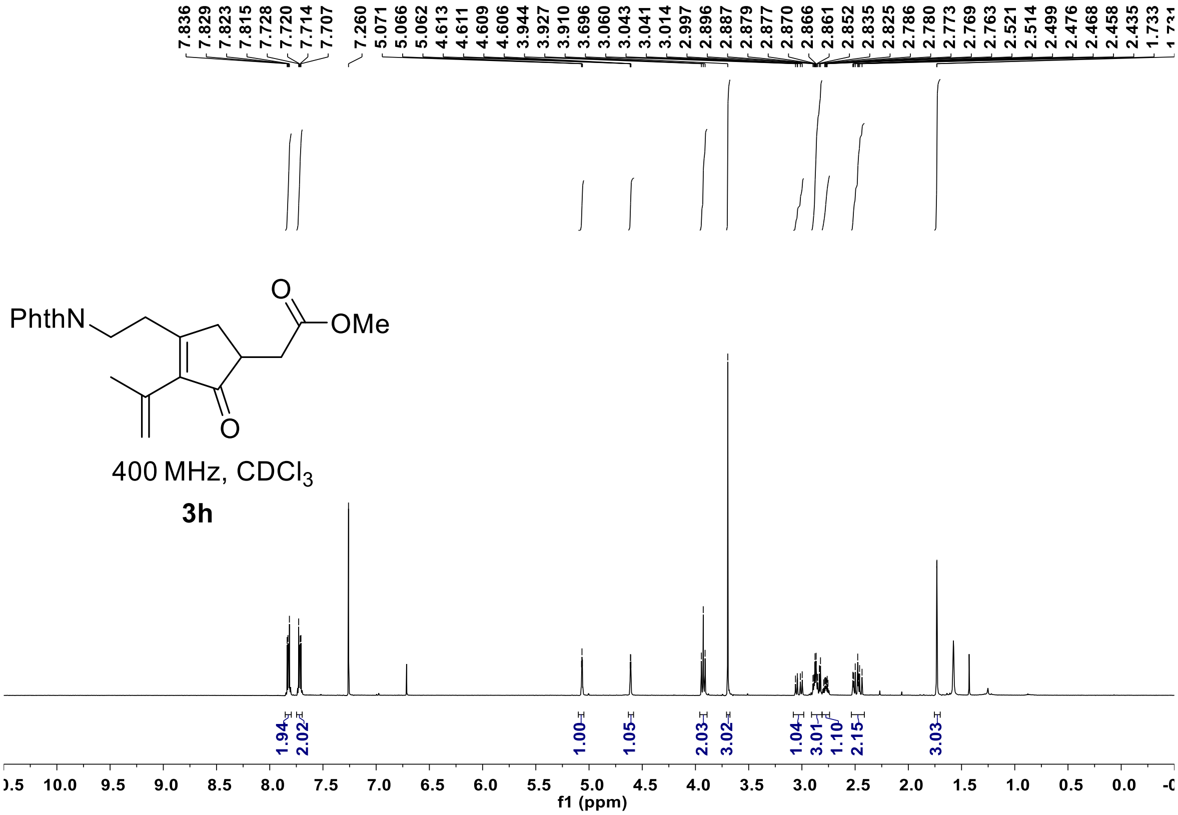
7.836
7.829
7.823
7.815
7.728
7.720
7.714
7.707
7.260
5.071
5.066
5.062
4.613
4.611
4.609
4.606
3.944
3.927
3.910
3.696
3.060
3.043
3.041
3.014
2.997
2.896
2.887
2.879
2.877
2.870
2.866
2.861
2.852
2.835
2.825
2.786
2.780
2.773
2.769
2.763
2.521
2.514
2.499
2.476
2.468
2.458
2.435
1.733
PhthN
O
OMe
O
400 MHz, CDCl3
3h
1.94
2.02
1.00
1.05
2.03
3.02
1.04
3.01
1.10
2.15
3.03
10.0
9.5
9.0
8.5
8.0
7.5
7.0
6.5
6.0
5.5
5.0
4.5
4.0
3.5
3.0
2.5
2.0
1.5
1.0
0.5
0.0
f1 (ppm)

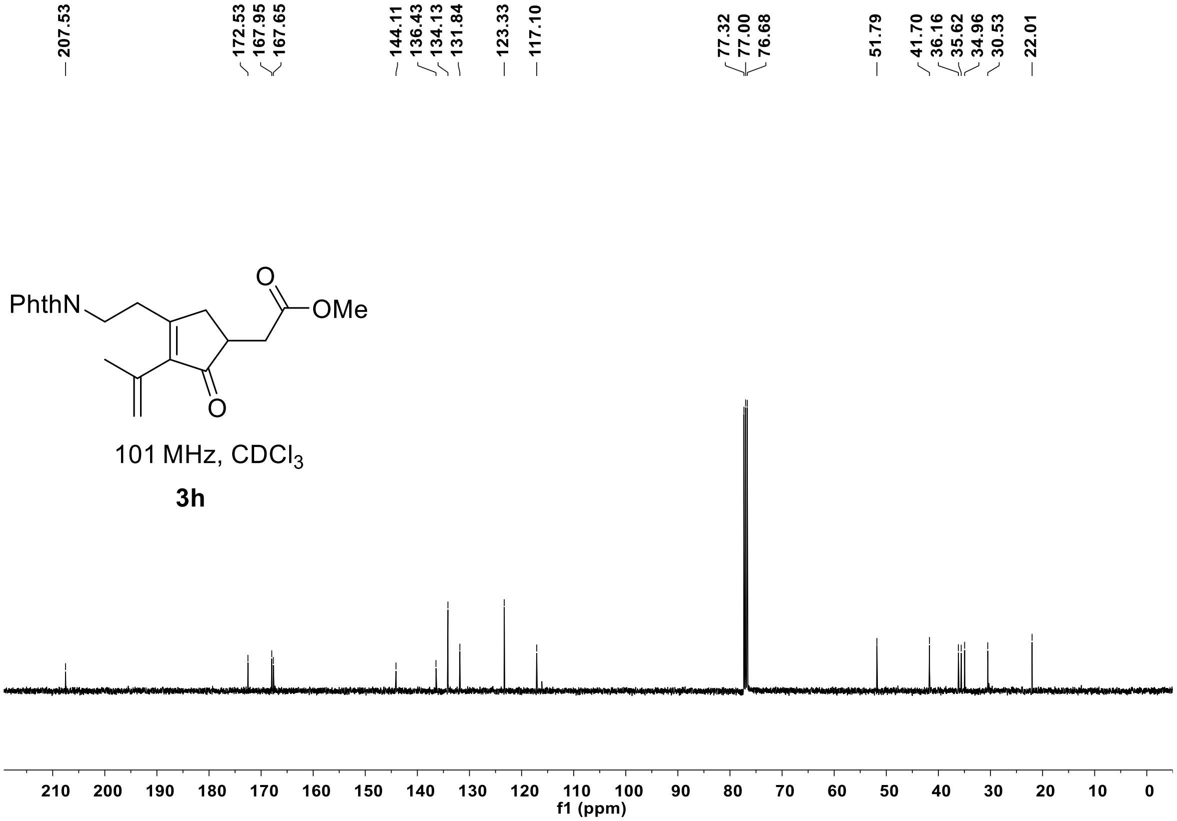
207.53
172.53
167.95
167.65
144.11
136.43
134.13
131.84
123.33
117.10
77.32
77.00
76.68
51.79
41.70
36.16
35.62
34.96
30.53
22.01
PhthN
O
OMe
O
101 MHz, CDCl3
3h
210
200
190
180
170
160
150
140
130
120
110
100
90
80
70
60
50
40
30
20
10
0
f1 (ppm)

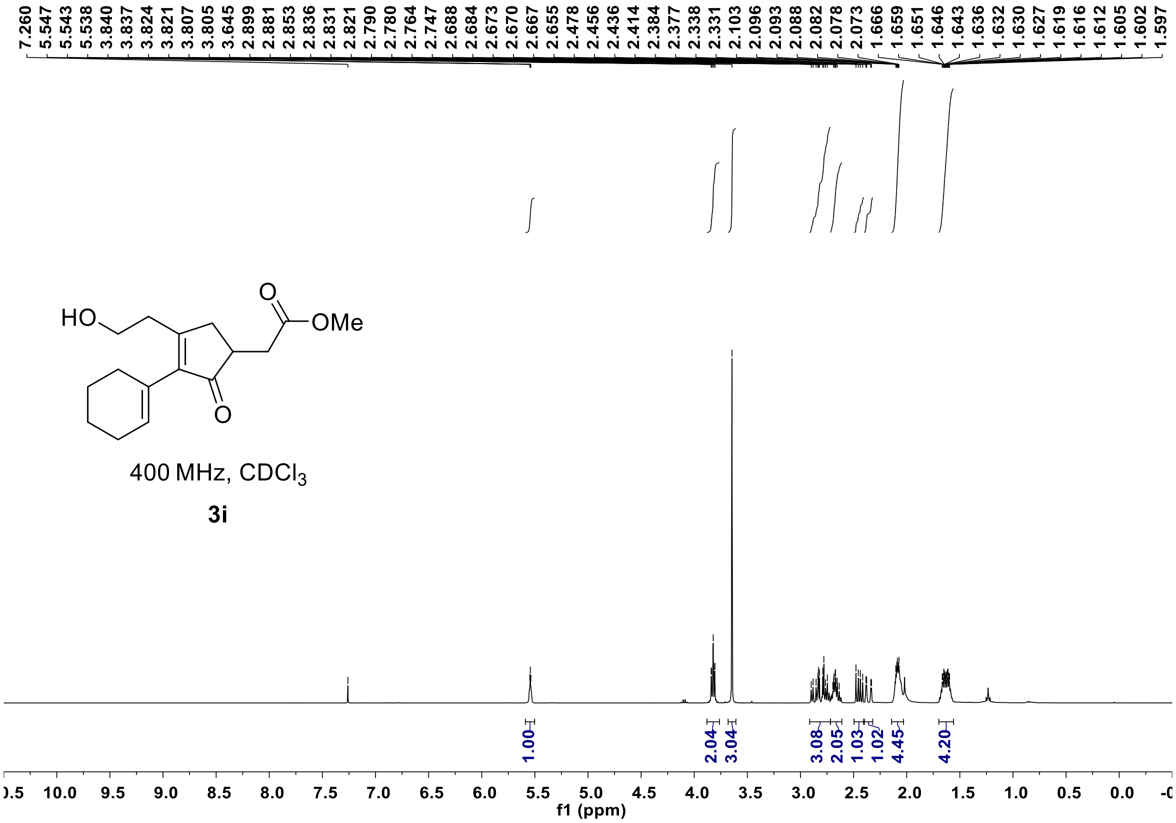
7.260
5.547
5.543
5.538
3.840
3.837
3.824
3.821
3.807
3.805
3.645
2.899
2.881
2.853
2.836
2.831
2.821
2.790
2.780
2.764
2.747
2.688
2.684
2.673
2.670
2.667
2.655
2.478
2.456
2.436
2.414
2.384
2.377
2.338
2.331
2.103
2.096
2.093
2.088
2.082
2.078
2.073
1.666
1.659
1.651
1.646
1.643
1.636
1.632
1.630
1.627
1.619
1.616
1.612
1.605
1.602
1.597
HO
O
OMe
O
400 MHz, CDCl3
3i
1.00
2.04
3.04
3.08
2.05
1.03
1.02
4.45
4.20
10.0 9.5 9.0 8.5 8.0 7.5 7.0 6.5 6.0 5.5 5.0 4.5 4.0 3.5 3.0 2.5 2.0 1.5 1.0 0.5 0.0
f1 (ppm)

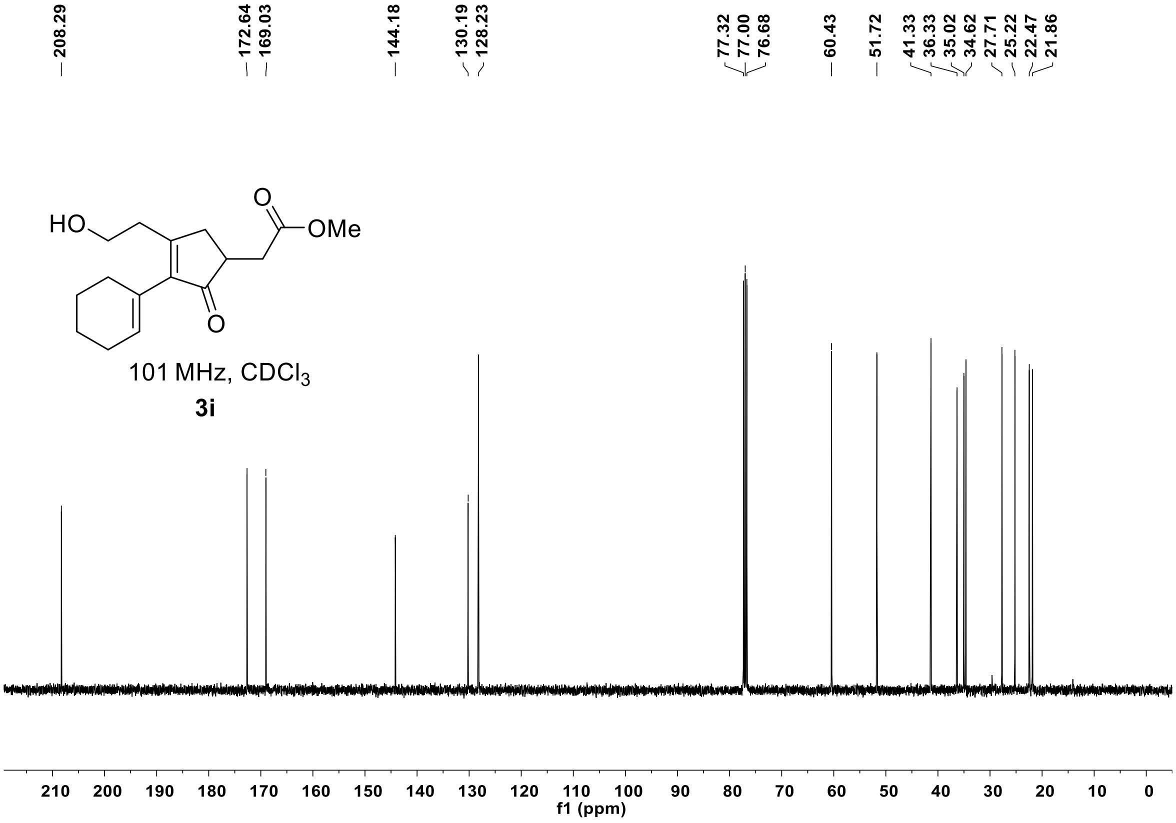
208.29
172.64
169.03
144.18
130.19
128.23
77.32
77.00
76.68
60.43
51.72
41.33
36.33
35.02
34.62
27.71
25.22
22.47
21.86
HO
O
OMe
O
101 MHz, CDCl3
3i
210 200 190 180 170 160 150 140 130 120 110 100 90 80 70 60 50 40 30 20 10 0
f1 (ppm)

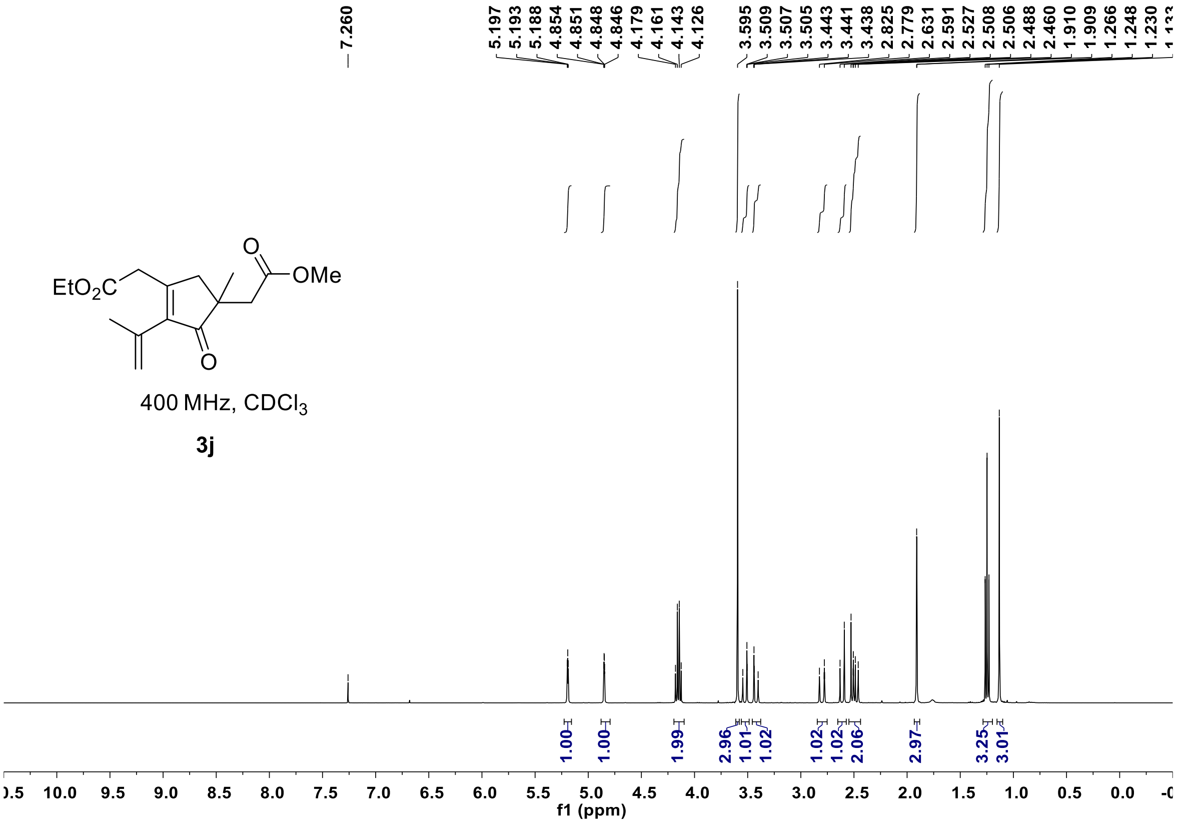

400 MHz, CDCl3
3j
f1 (ppm)

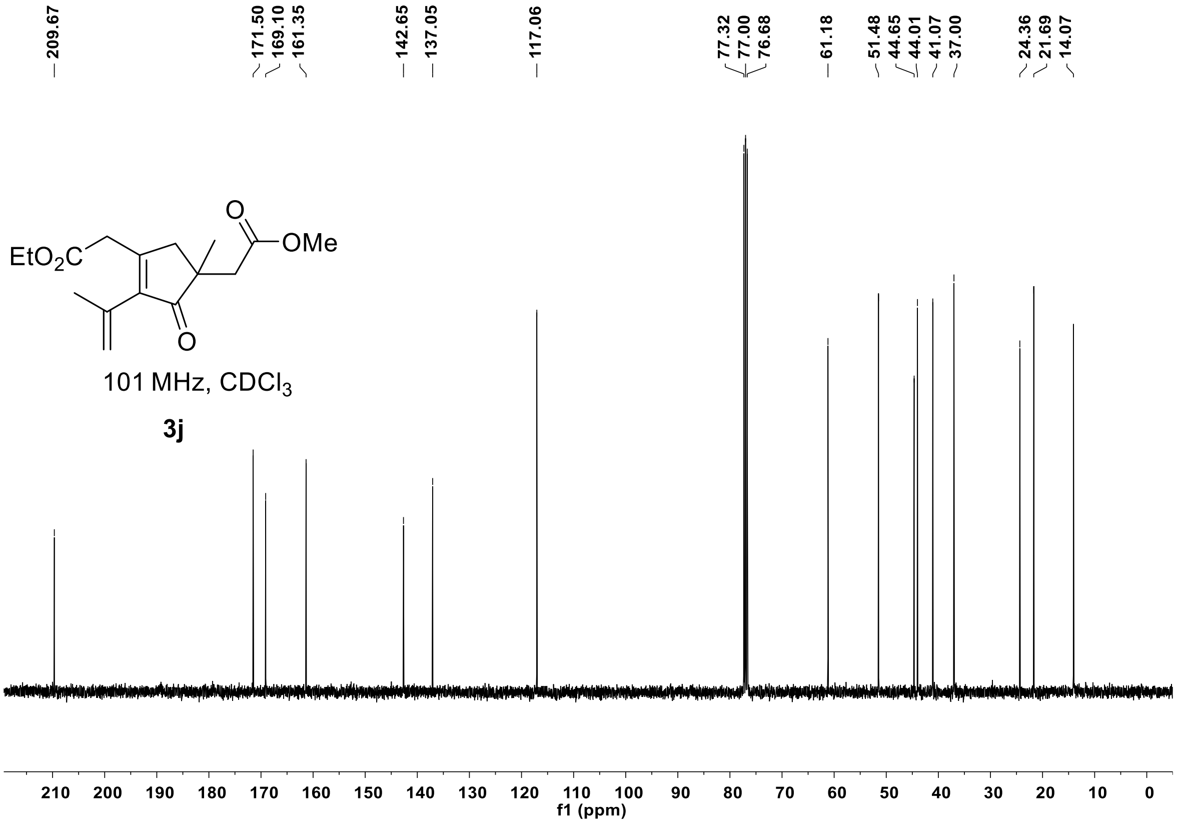

101 MHz, CDCl3
3j
f1 (ppm)

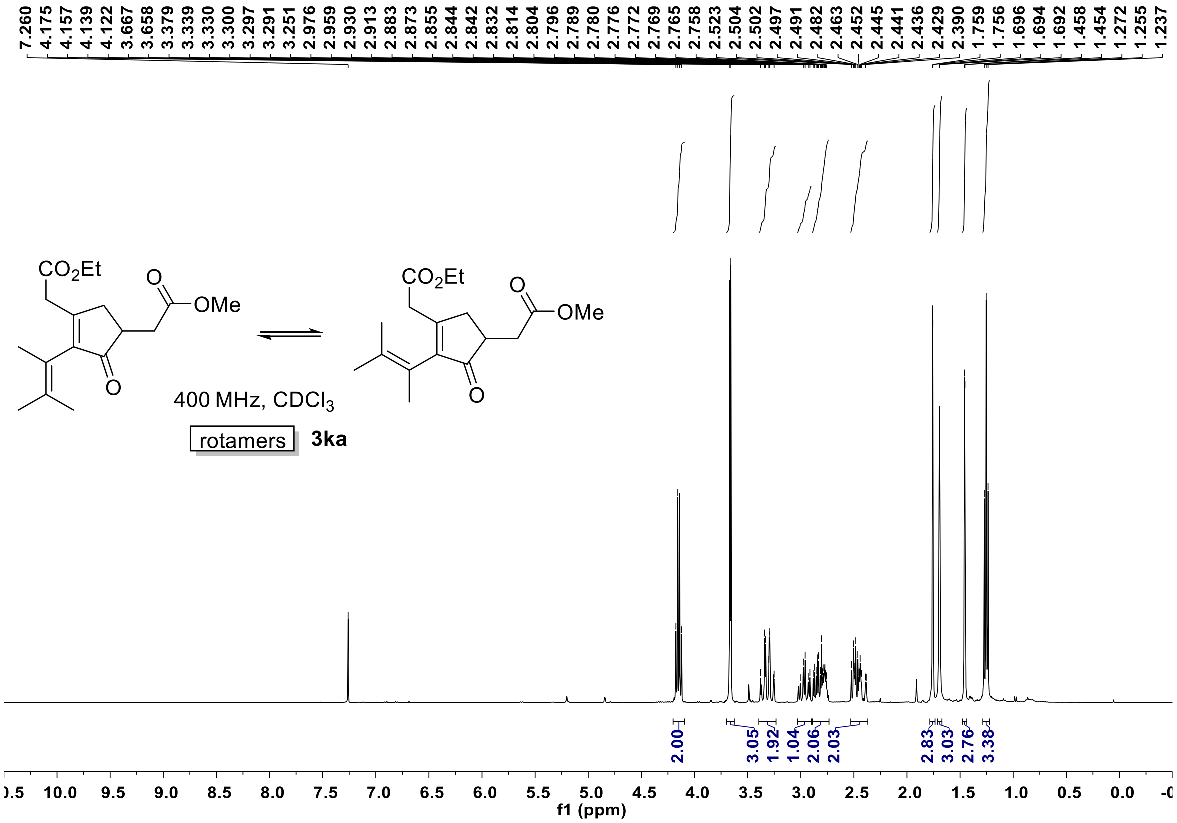
7.260
4.175
4.157
4.139
4.122
3.667
3.658
3.379
3.339
3.330
3.300
3.297
3.291
3.251
2.976
2.959
2.930
2.913
2.883
2.873
2.855
2.844
2.842
2.832
2.814
2.804
2.796
2.789
2.780
2.776
2.772
2.769
2.765
2.758
2.523
2.504
2.502
2.497
2.491
2.482
2.463
2.452
2.445
2.441
2.436
2.429
2.390
1.759
1.756
1.696
1.694
1.692
1.458
1.454
1.272
1.255
1.237
CO_2Et
OMe
O
400 MHz, $CDCl_3$
rotamers
3ka
2.00
3.05
1.92
1.04
2.06
2.03
2.83
3.03
2.76
3.38
f1 (ppm)

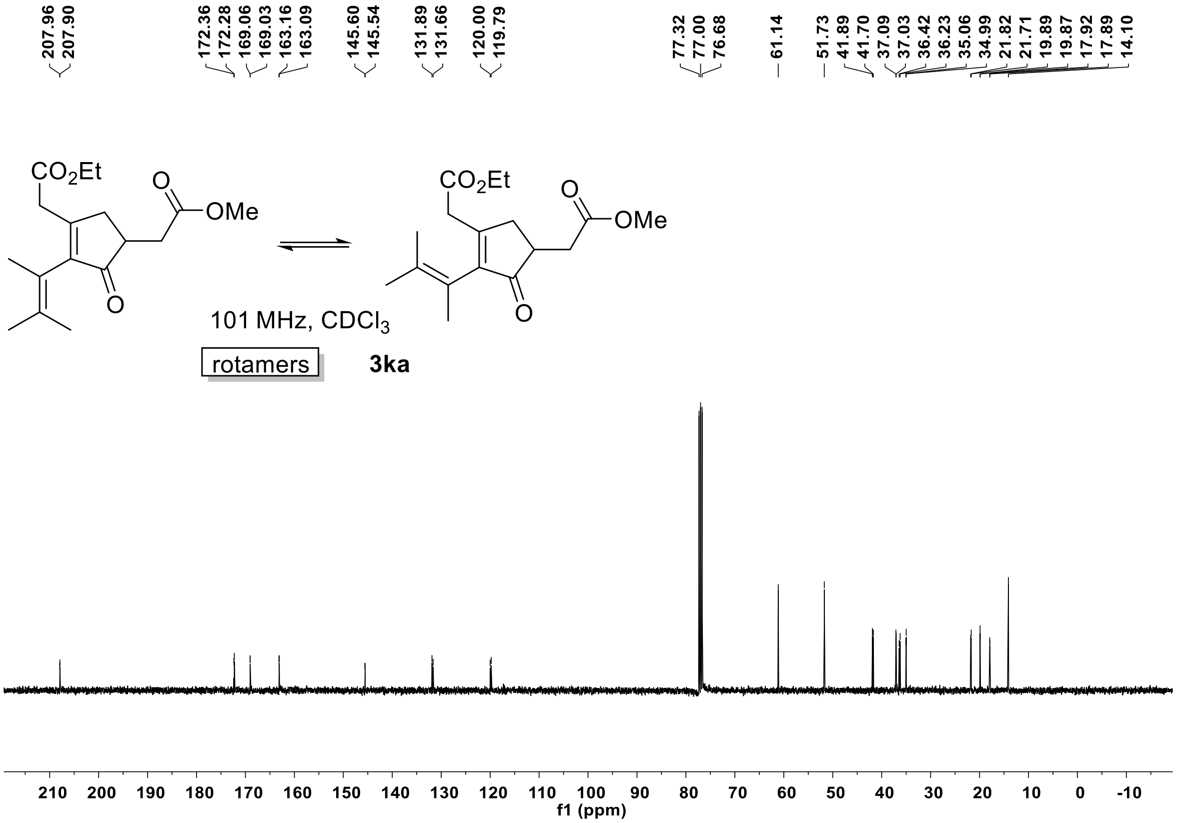
207.96
207.90
172.36
172.28
169.06
169.03
163.16
163.09
145.60
145.54
131.89
131.66
120.00
119.79
77.32
77.00
76.68
61.14
51.73
41.89
41.70
37.09
37.03
36.42
36.23
35.06
34.99
21.82
21.71
19.89
19.87
17.92
17.89
14.10
CO_2Et
OMe
O
101 MHz, $CDCl_3$
rotamers
3ka
f1 (ppm)

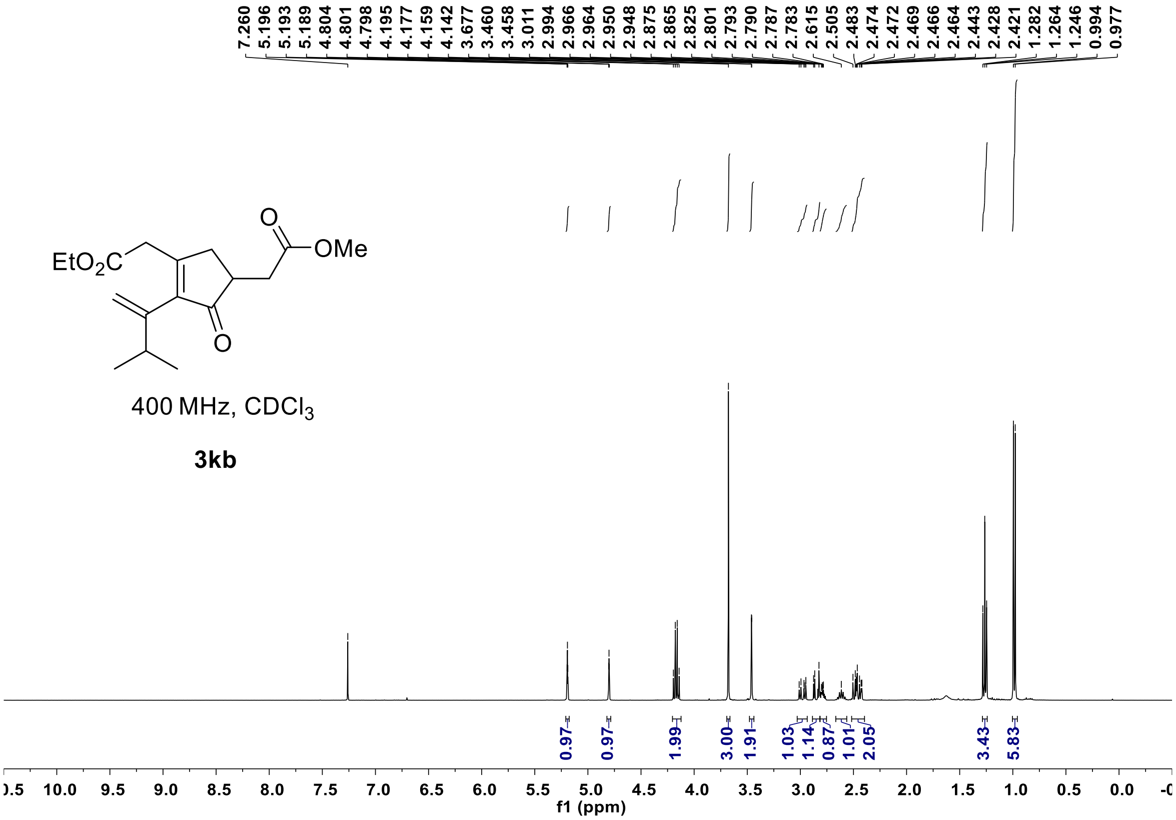

EtO2C
OMe
400 MHz, CDCl3
3kb
f1 (ppm)

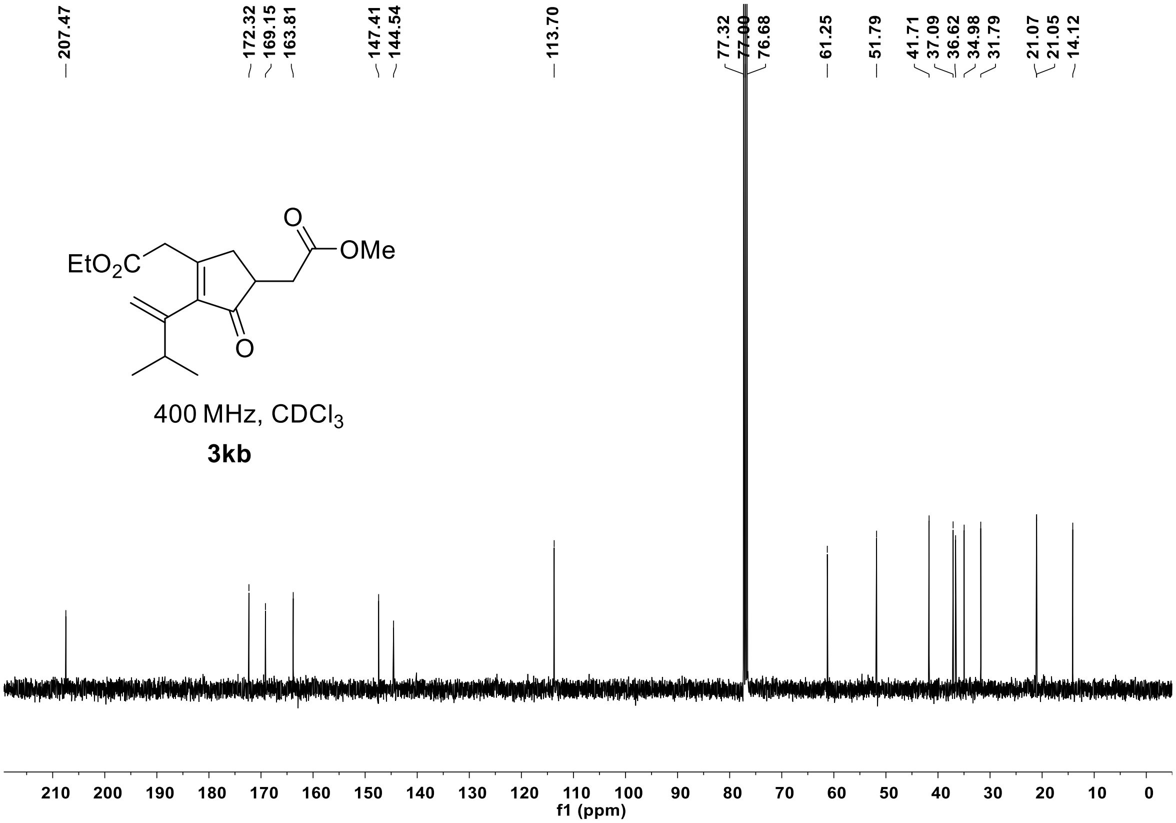

EtO2C
OMe
400 MHz, CDCl3
3kb
f1 (ppm)

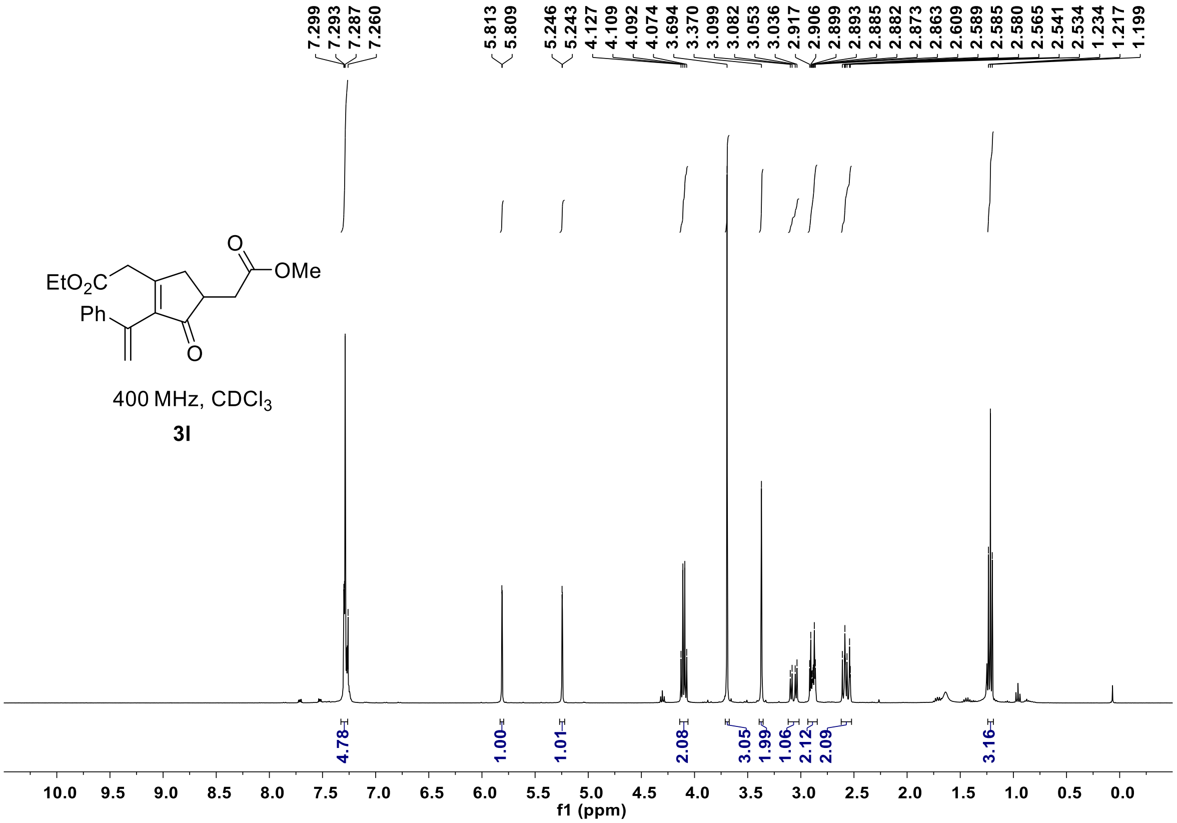
7.299
7.293
7.287
7.260
5.813
5.809
5.246
5.243
4.127
4.109
4.092
4.074
3.694
3.370
3.099
3.082
3.053
3.036
2.917
2.906
2.899
2.893
2.885
2.882
2.873
2.863
2.609
2.589
2.585
2.580
2.565
2.541
2.534
1.234
1.217
1.199
EtO2C
O
OMe
Ph
O
400 MHz, CDCl3
3l
4.78
1.00
1.01
2.08
3.05
1.99
1.06
2.12
2.09
3.16
10.0 9.5 9.0 8.5 8.0 7.5 7.0 6.5 6.0 5.5 5.0 4.5 4.0 3.5 3.0 2.5 2.0 1.5 1.0 0.5 0.0
f1 (ppm)

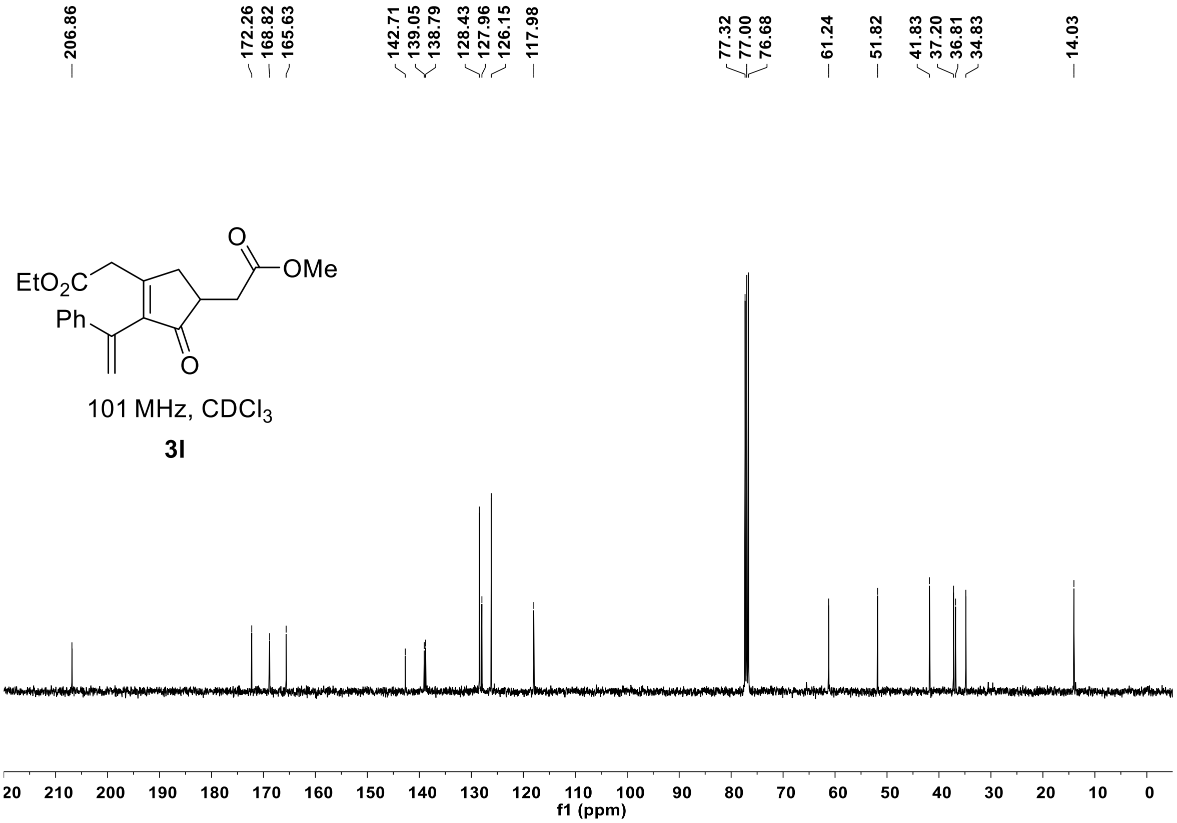
206.86
172.26
168.82
165.63
142.71
139.05
138.79
128.43
127.96
126.15
117.98
77.32
77.00
76.68
61.24
51.82
41.83
37.20
36.81
34.83
14.03
EtO2C
O
OMe
Ph
O
101 MHz, CDCl3
3l
20 210 200 190 180 170 160 150 140 130 120 110 100 90 80 70 60 50 40 30 20 10 0
f1 (ppm)

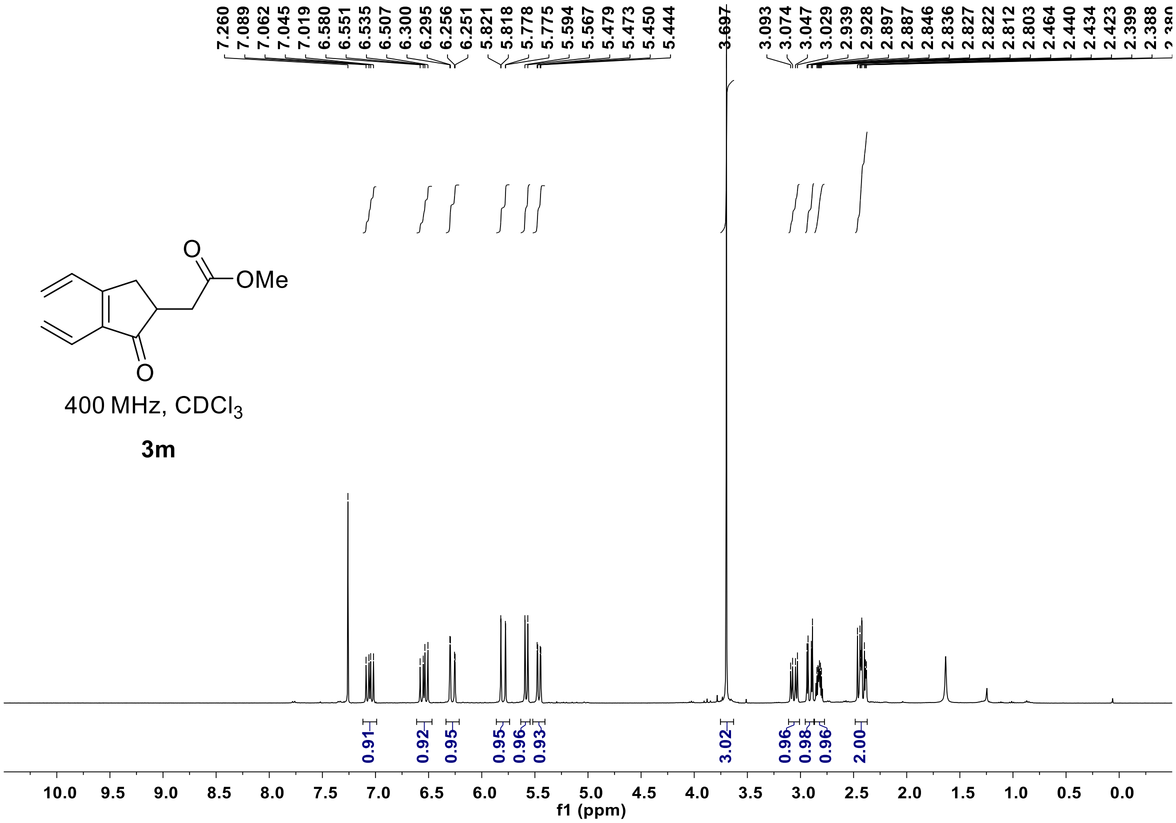
7.260
7.089
7.062
7.045
7.019
6.580
6.551
6.535
6.507
6.300
6.295
6.256
6.251
5.821
5.818
5.778
5.775
5.594
5.567
5.479
5.473
5.450
5.444
3.697
3.093
3.074
3.047
3.029
2.939
2.928
2.897
2.887
2.846
2.836
2.827
2.822
2.812
2.803
2.464
2.440
2.434
2.423
2.399
2.388
OMe
400 MHz, CDCl3
3m
0.91
0.92
0.95
0.95
0.96
0.93
3.02
0.96
0.98
0.96
2.00
f1 (ppm)

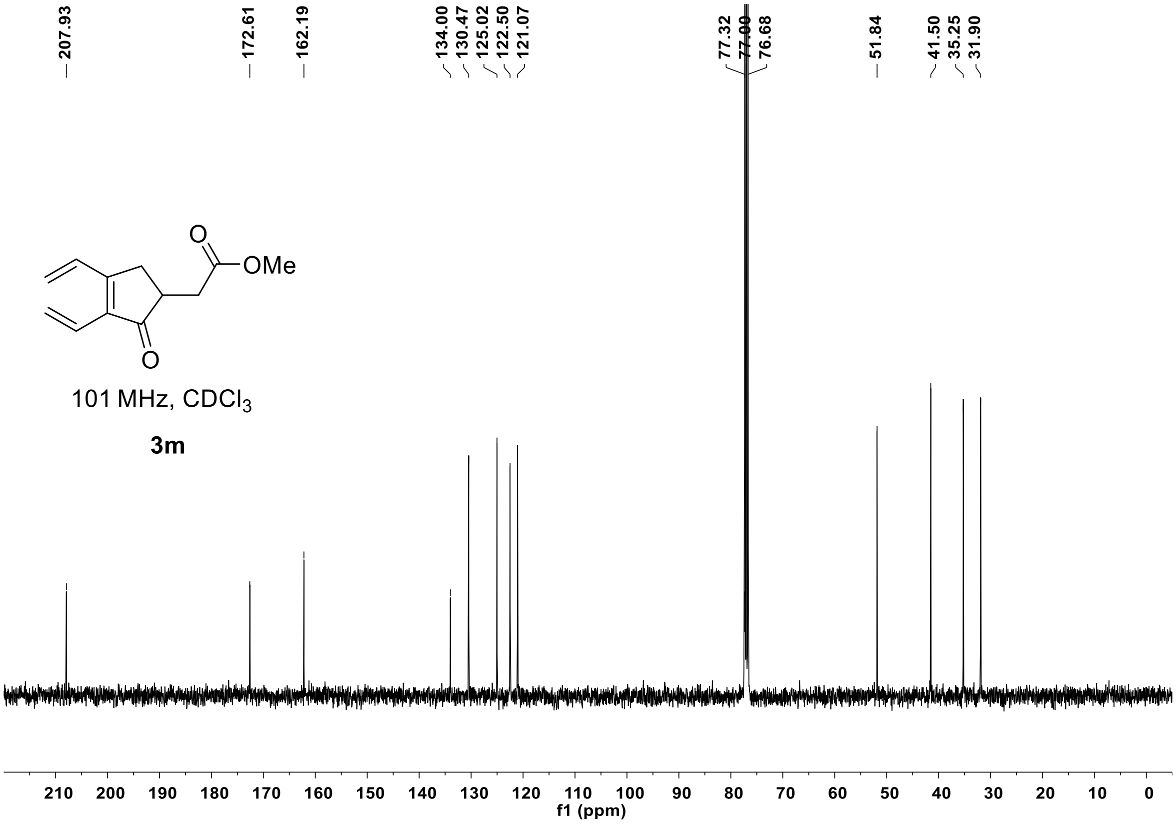
207.93
172.61
162.19
134.00
130.47
125.02
122.50
121.07
77.32
77.00
76.68
51.84
41.50
35.25
31.90
OMe
101 MHz, CDCl3
3m
f1 (ppm)

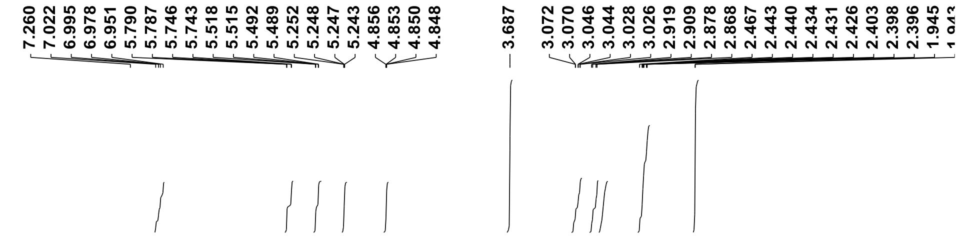

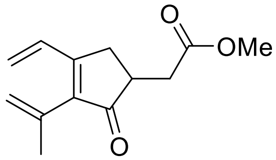

400 MHz, $CDCl_3$

3n

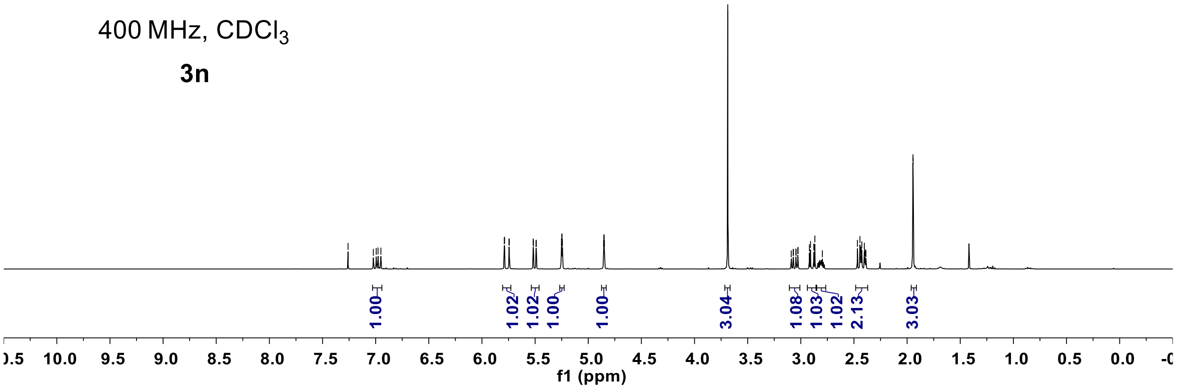

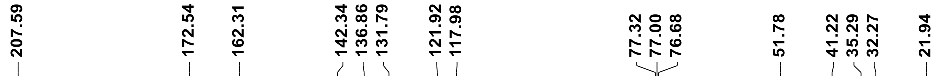

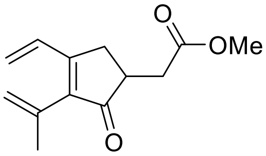

101 MHz, $CDCl_3$

3n

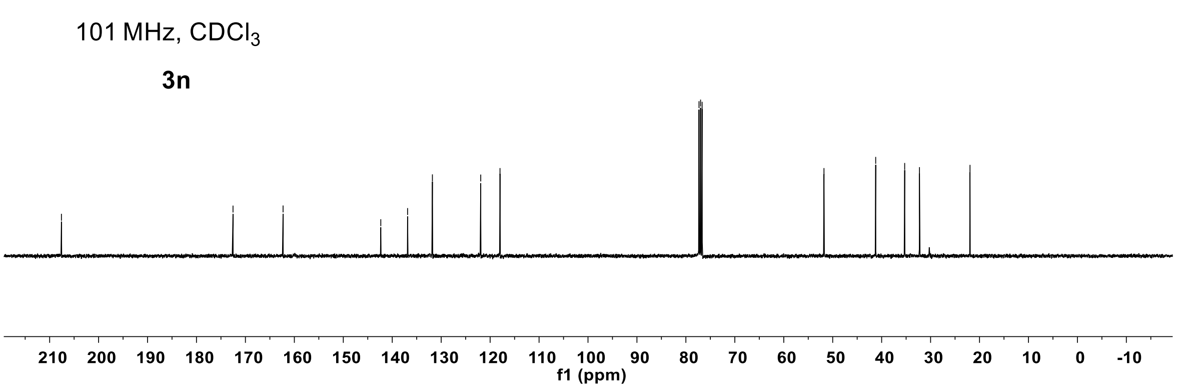

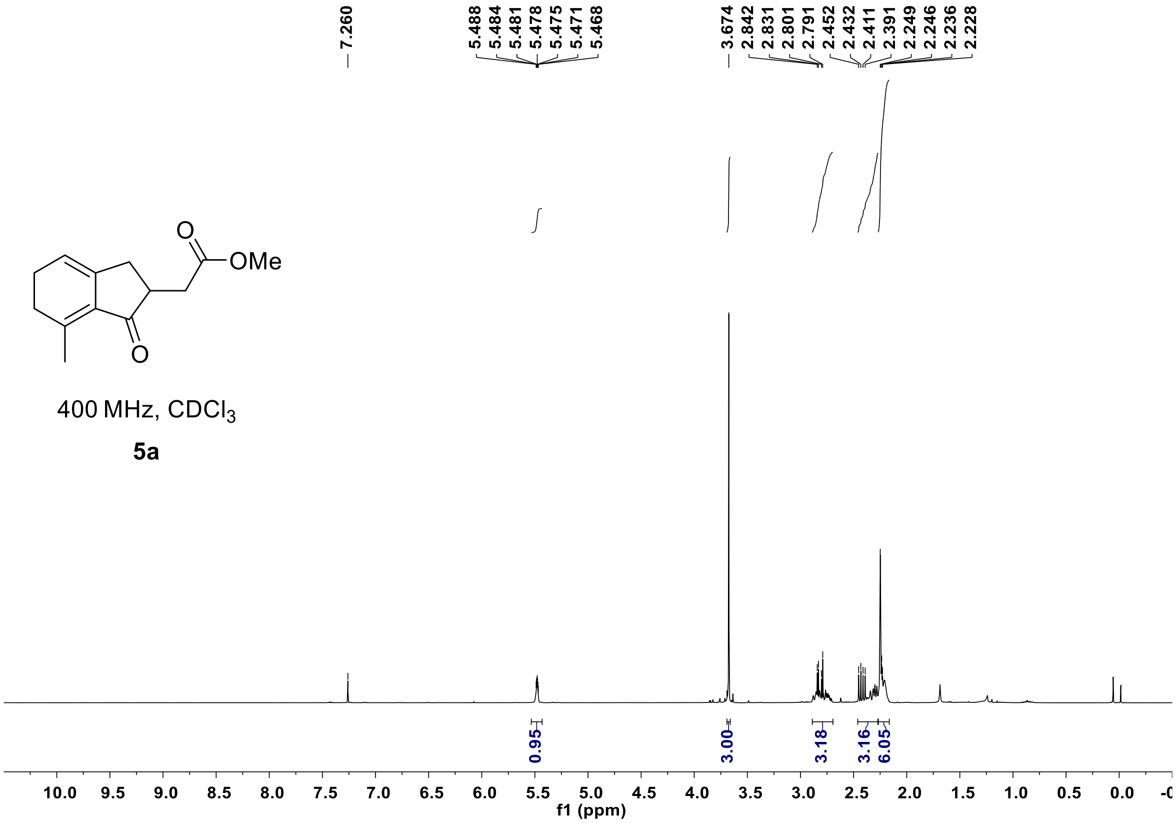

7.260
5.488
5.484
5.481
5.478
5.475
5.471
5.468
3.674
2.842
2.831
2.801
2.791
2.452
2.432
2.411
2.391
2.249
2.246
2.236
2.228
OMe
O
400 MHz, CDCl3
5a
0.95
3.00
3.18
3.16
6.05
f1 (ppm)

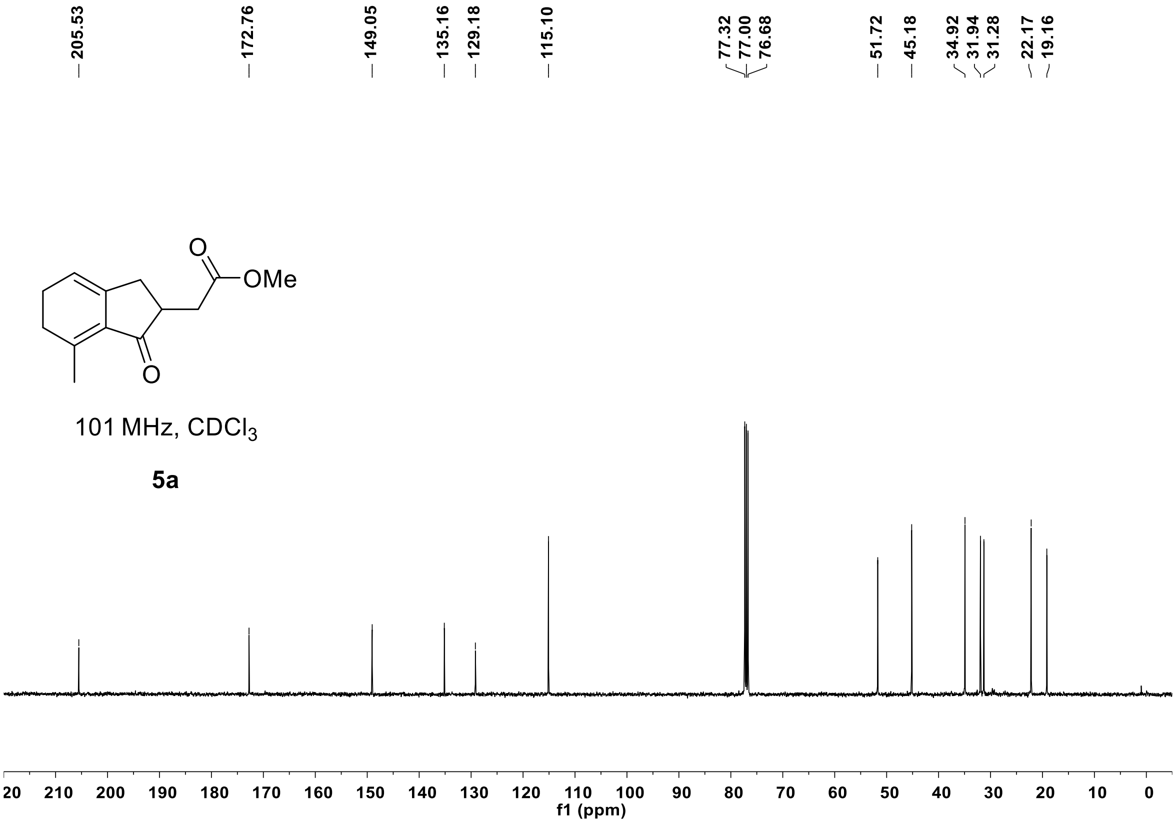

205.53
172.76
149.05
135.16
129.18
115.10
77.32
77.00
76.68
51.72
45.18
34.92
31.94
31.28
22.17
19.16
OMe
O
101 MHz, CDCl3
5a
f1 (ppm)

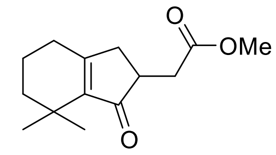

400 MHz, $CDCl_3$

6a

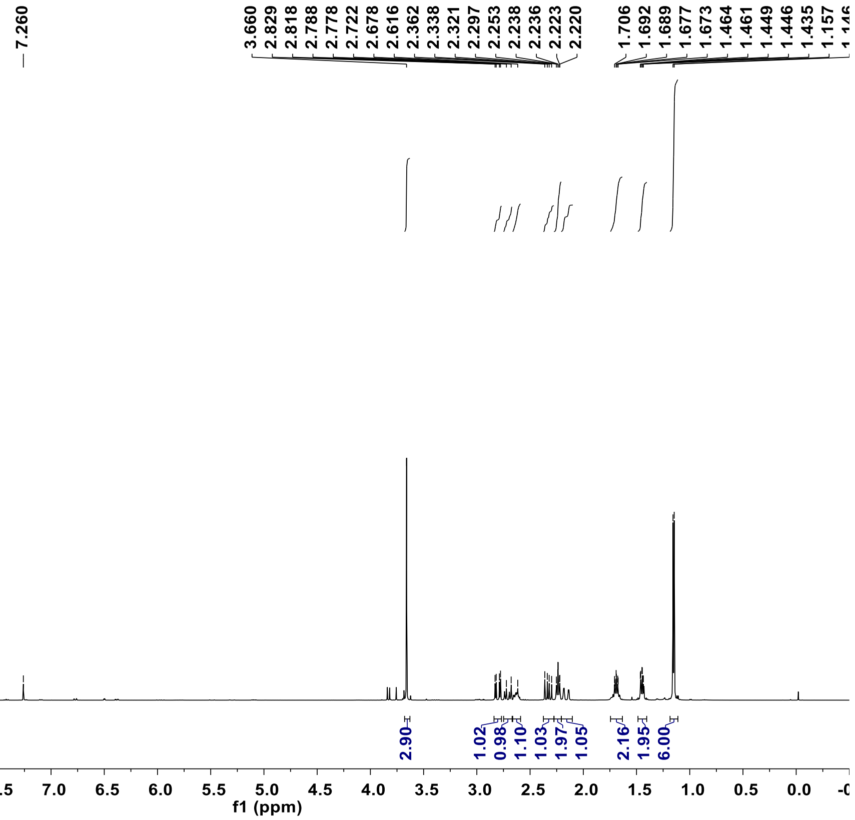

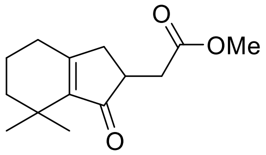

101 MHz, $CDCl_3$

6a

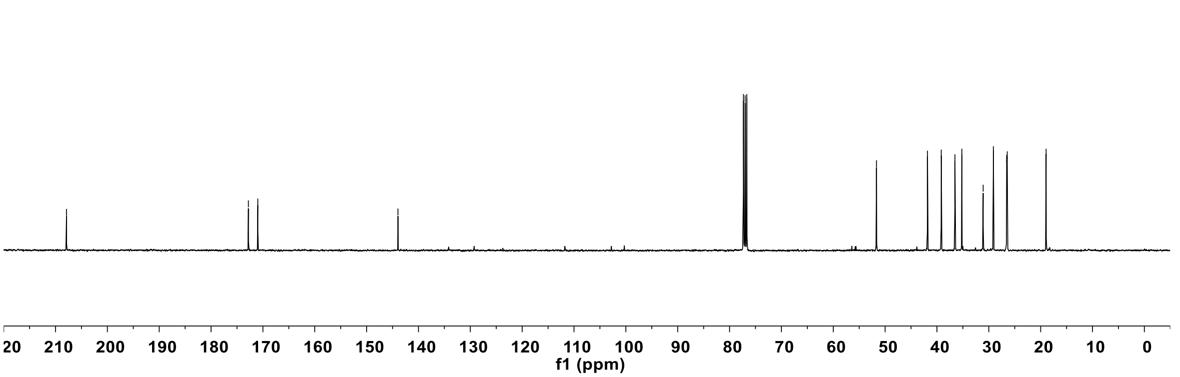

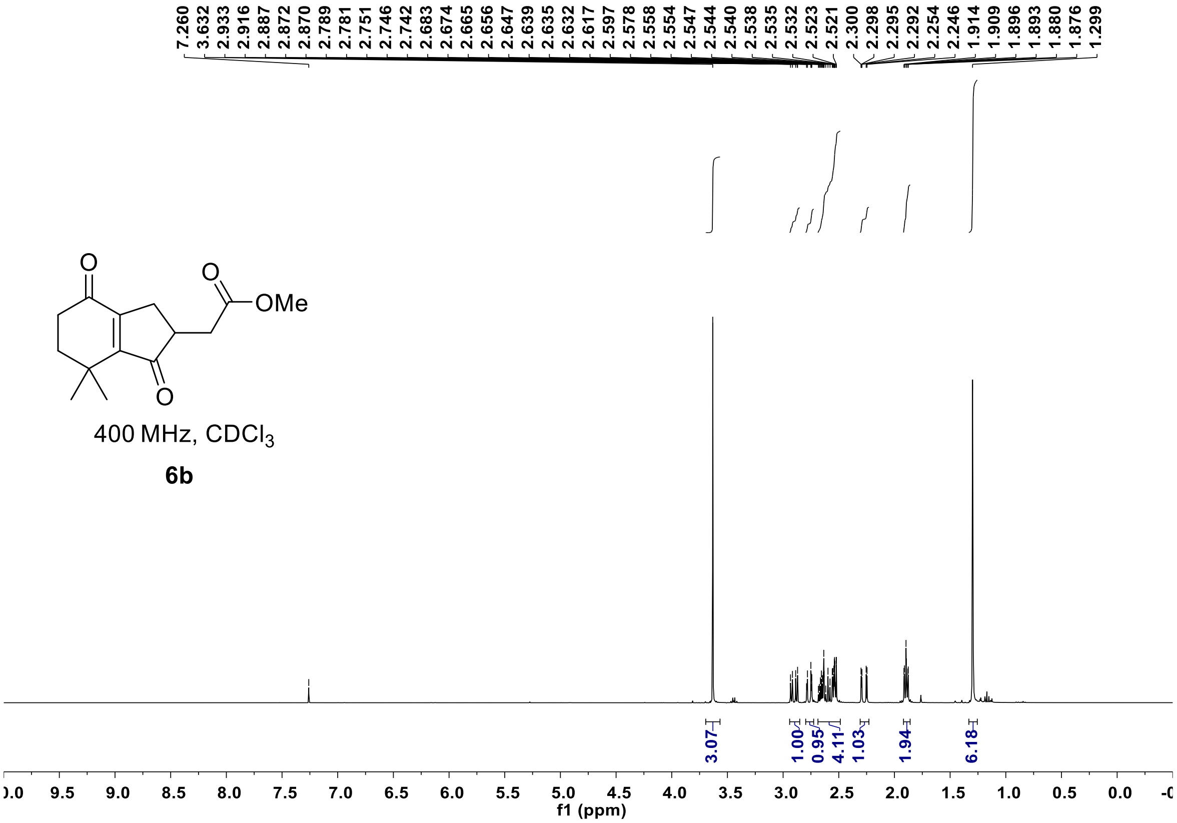
7.260 3.632 2.933 2.916 2.887 2.872 2.870 2.789 2.781 2.751 2.746 2.742 2.683 2.674 2.665 2.656 2.647 2.639 2.635 2.632 2.617 2.597 2.578 2.558 2.554 2.547 2.544 2.540 2.538 2.535 2.532 2.523 2.521 2.300 2.298 2.295 2.292 2.254 2.246 1.914 1.909 1.896 1.893 1.880 1.876 1.299
O
O
OMe
O
400 MHz, $CDCl_3$
6b
3.07
1.00
0.95
4.11
1.03
1.94
6.18
f1 (ppm)

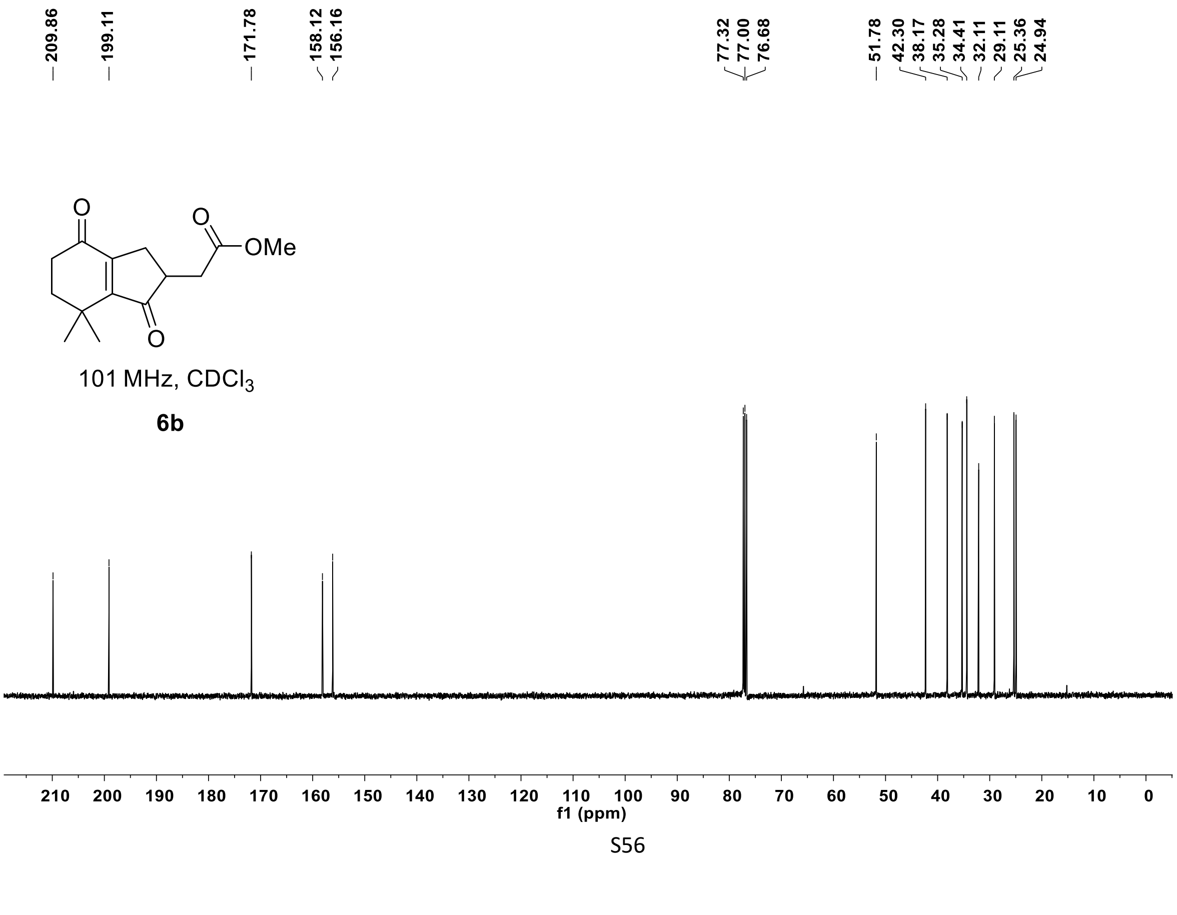
209.86
199.11
171.78
158.12
156.16
77.32
77.00
76.68
51.78
42.30
38.17
35.28
34.41
32.11
29.11
25.36
24.94
O
O
OMe
O
101 MHz, $CDCl_3$
6b
f1 (ppm)

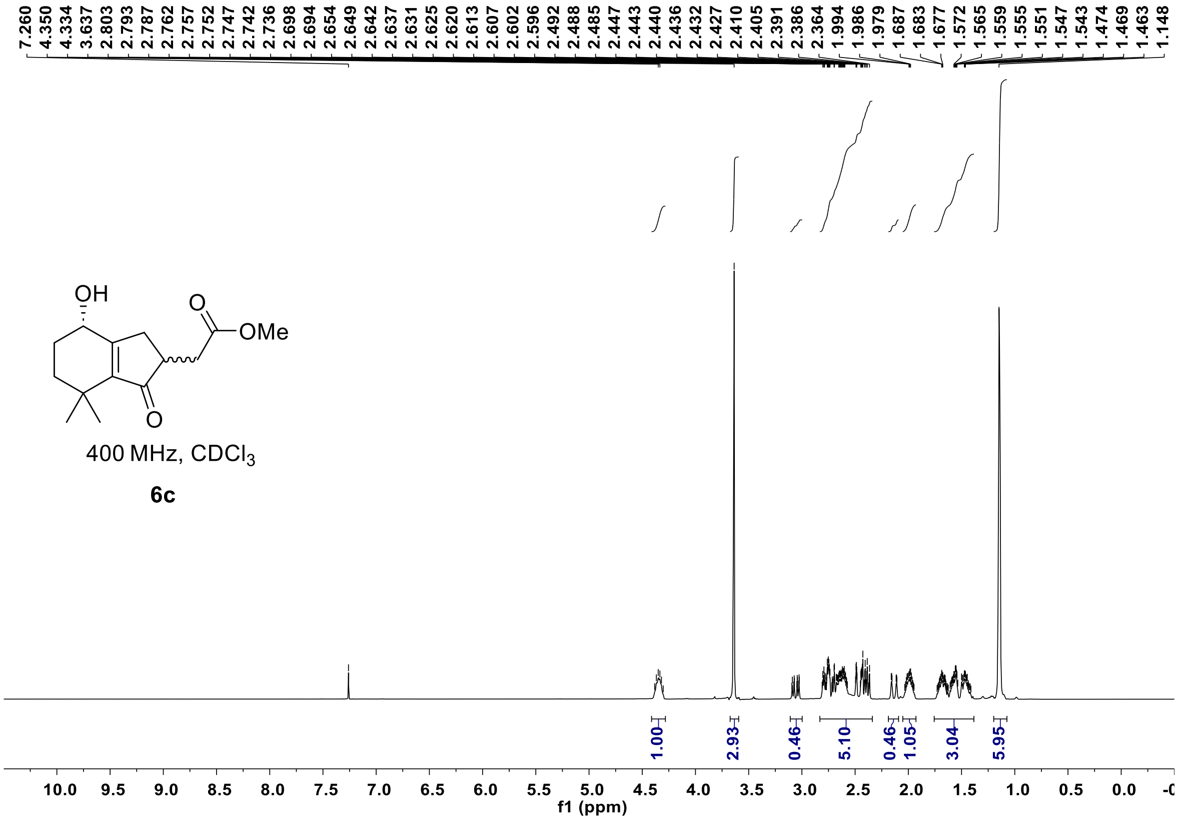
7.260 4.350 4.334 3.637 2.803 2.793 2.787 2.762 2.757 2.752 2.747 2.742 2.736 2.698 2.694 2.654 2.649 2.642 2.637 2.631 2.625 2.620 2.613 2.607 2.602 2.596 2.492 2.488 2.485 2.447 2.443 2.440 2.436 2.432 2.427 2.410 2.405 2.391 2.386 2.364 1.994 1.986 1.979 1.687 1.683 1.677 1.572 1.565 1.559 1.555 1.551 1.547 1.543 1.474 1.469 1.463 1.148
OH
O
OMe
O
400 MHz, CDCl3
6c
1.00 2.93 0.46 5.10 0.46 1.05 3.04 5.95
10.0 9.5 9.0 8.5 8.0 7.5 7.0 6.5 6.0 5.5 5.0 4.5 4.0 3.5 3.0 2.5 2.0 1.5 1.0 0.5 0.0
f1 (ppm)

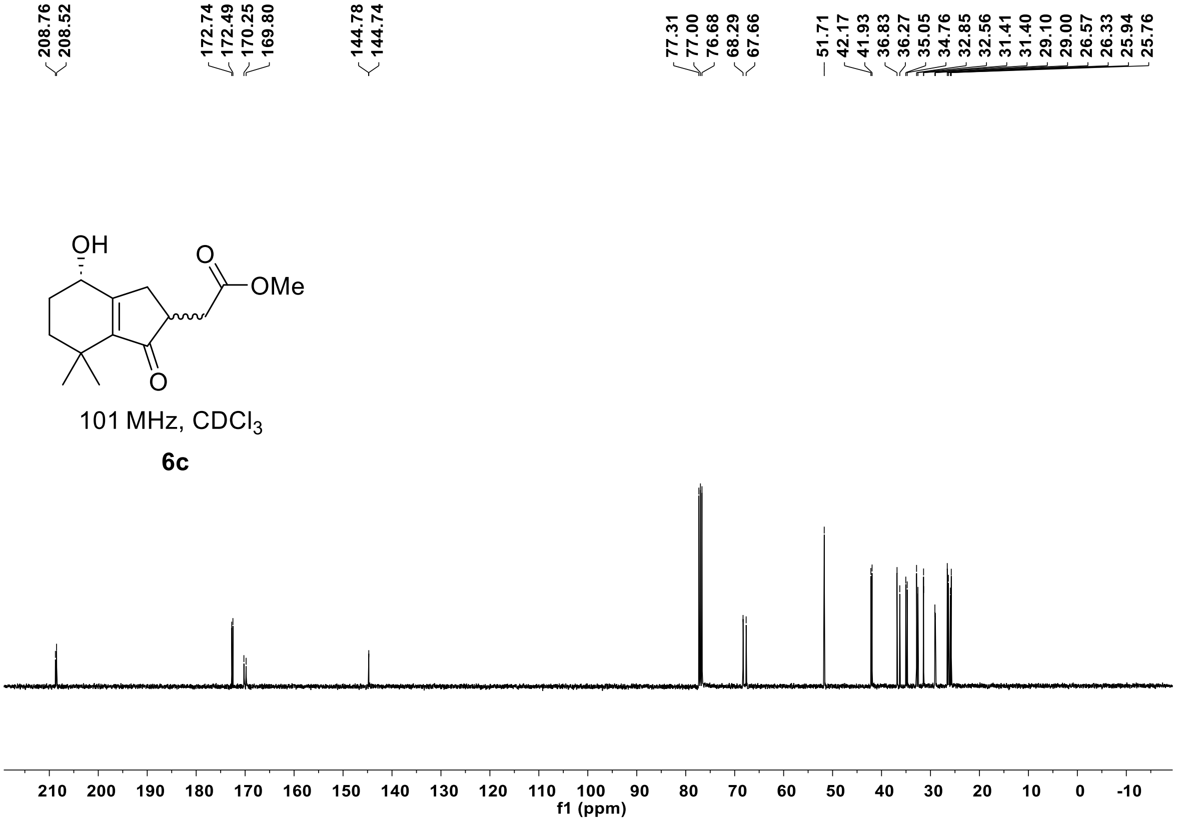
208.76 208.52 172.74 172.49 170.25 169.80 144.78 144.74 77.31 77.00 76.68 68.29 67.66 51.71 42.17 41.93 36.83 36.27 35.05 34.76 32.85 32.56 31.41 31.40 29.10 29.00 26.57 26.33 25.94 25.76
OH
O
OMe
O
101 MHz, CDCl3
6c
210 200 190 180 170 160 150 140 130 120 110 100 90 80 70 60 50 40 30 20 10 0 -10
f1 (ppm)

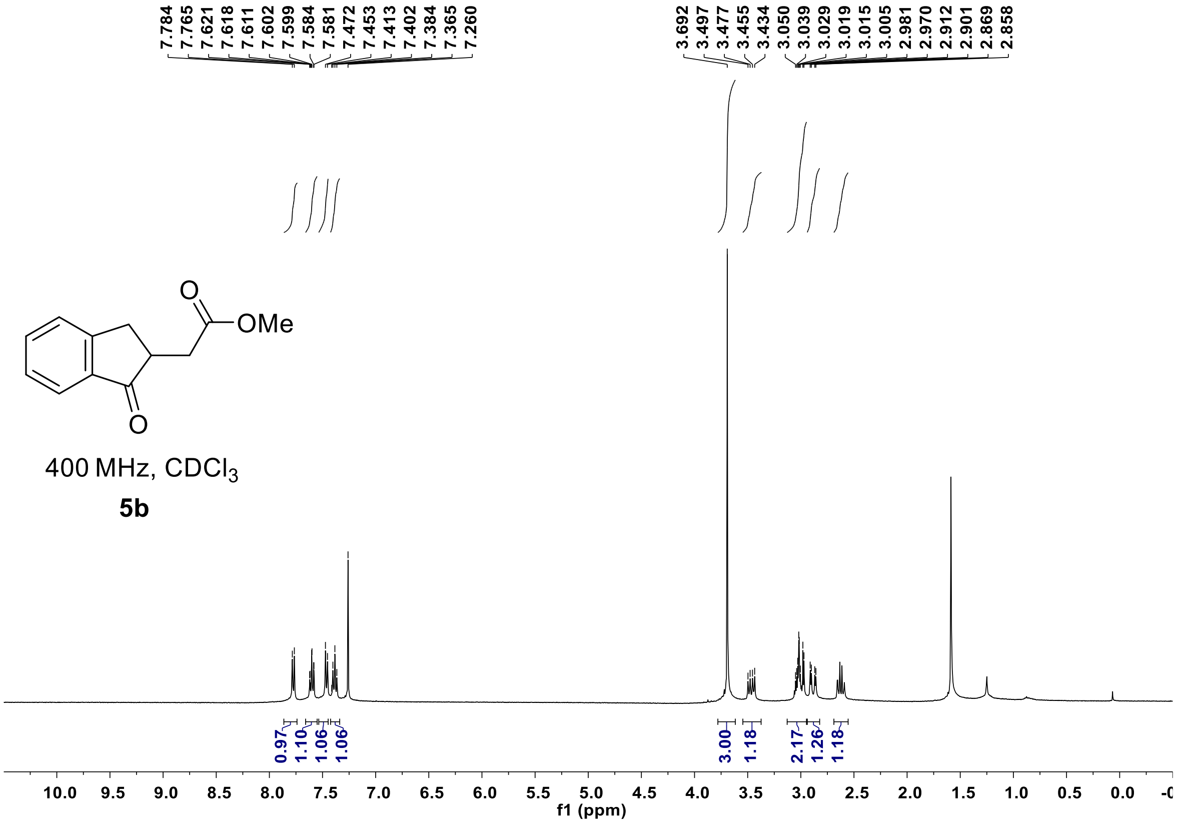
7.784 7.765 7.621 7.618 7.611 7.602 7.599 7.584 7.581 7.472 7.453 7.413 7.402 7.384 7.365 7.260
3.692 3.497 3.477 3.455 3.434 3.050 3.039 3.029 3.019 3.015 3.005 2.981 2.970 2.912 2.901 2.869 2.858
O
OMe
O
400 MHz, CDCl3
5b
0.97 1.10 1.06 1.06
3.00 1.18
2.17 1.26 1.18
10.0 9.5 9.0 8.5 8.0 7.5 7.0 6.5 6.0 5.5 5.0 4.5 4.0 3.5 3.0 2.5 2.0 1.5 1.0 0.5 0.0
f1 (ppm)

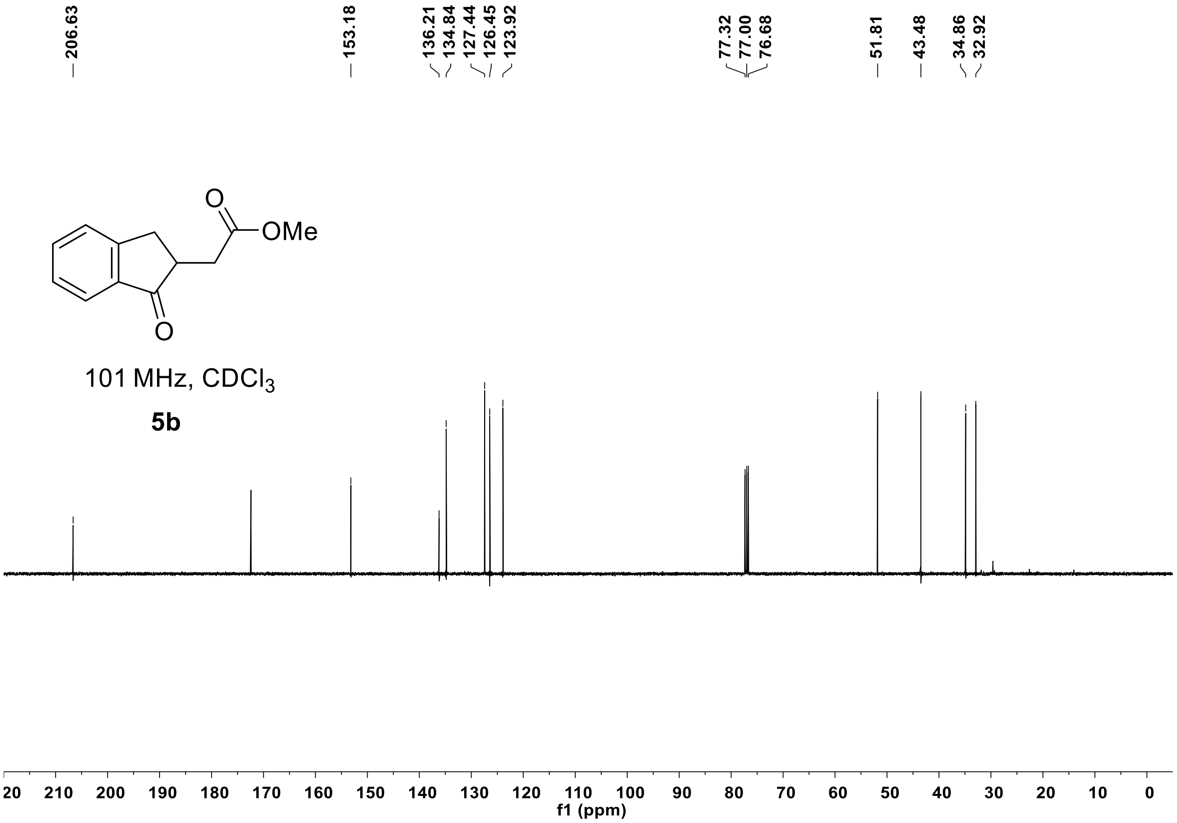
206.63
153.18
136.21 134.84 127.44 126.45 123.92
77.32 77.00 76.68
51.81
43.48
34.86 32.92
O
OMe
O
101 MHz, CDCl3
5b
20 210 200 190 180 170 160 150 140 130 120 110 100 90 80 70 60 50 40 30 20 10 0
f1 (ppm)

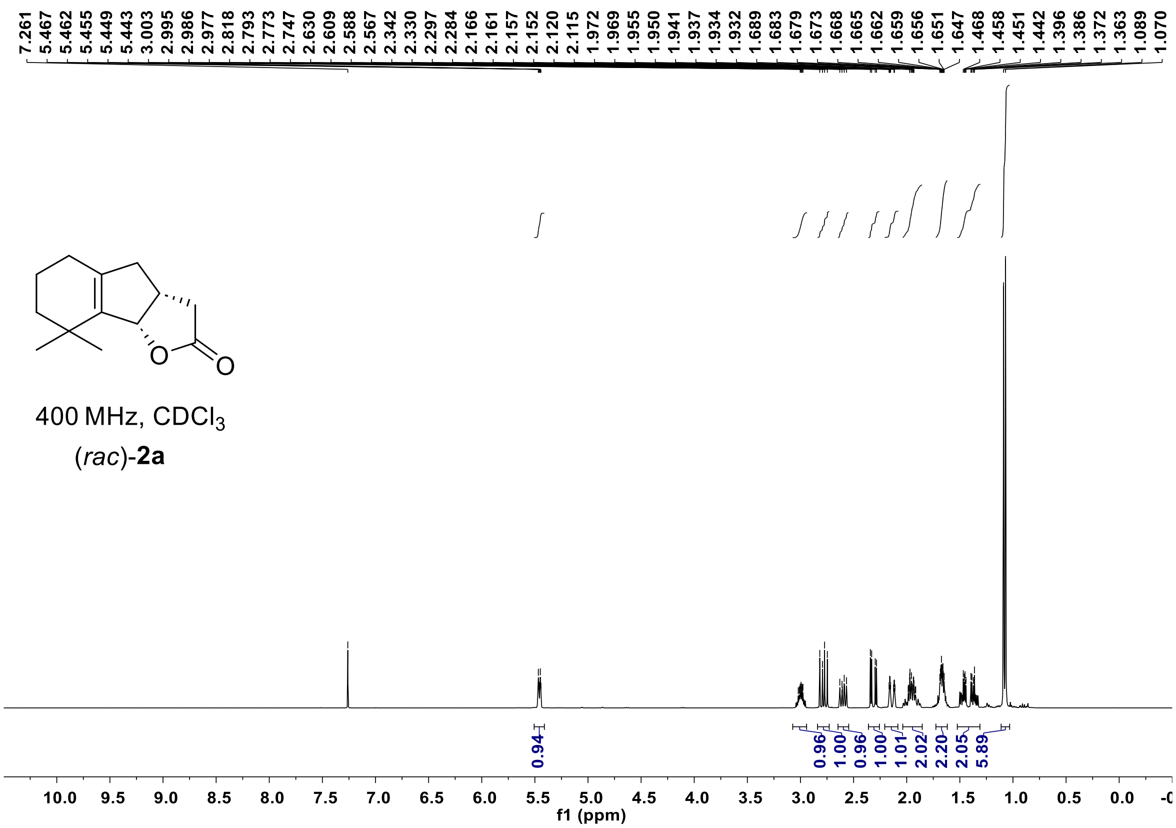
7.261
5.467
5.462
5.455
5.449
5.443
3.003
2.995
2.986
2.977
2.818
2.793
2.773
2.747
2.630
2.609
2.588
2.567
2.342
2.330
2.297
2.284
2.166
2.161
2.157
2.152
2.120
2.115
1.972
1.969
1.955
1.950
1.941
1.937
1.934
1.932
1.689
1.683
1.679
1.673
1.668
1.665
1.662
1.659
1.656
1.651
1.647
1.468
1.458
1.451
1.442
1.396
1.386
1.372
1.363
1.089
1.070
400 MHz, $CDCl_3$
(*rac*)-**2a**
0.94
0.96
1.00
0.96
1.00
1.01
2.02
2.20
2.05
5.89
f1 (ppm)

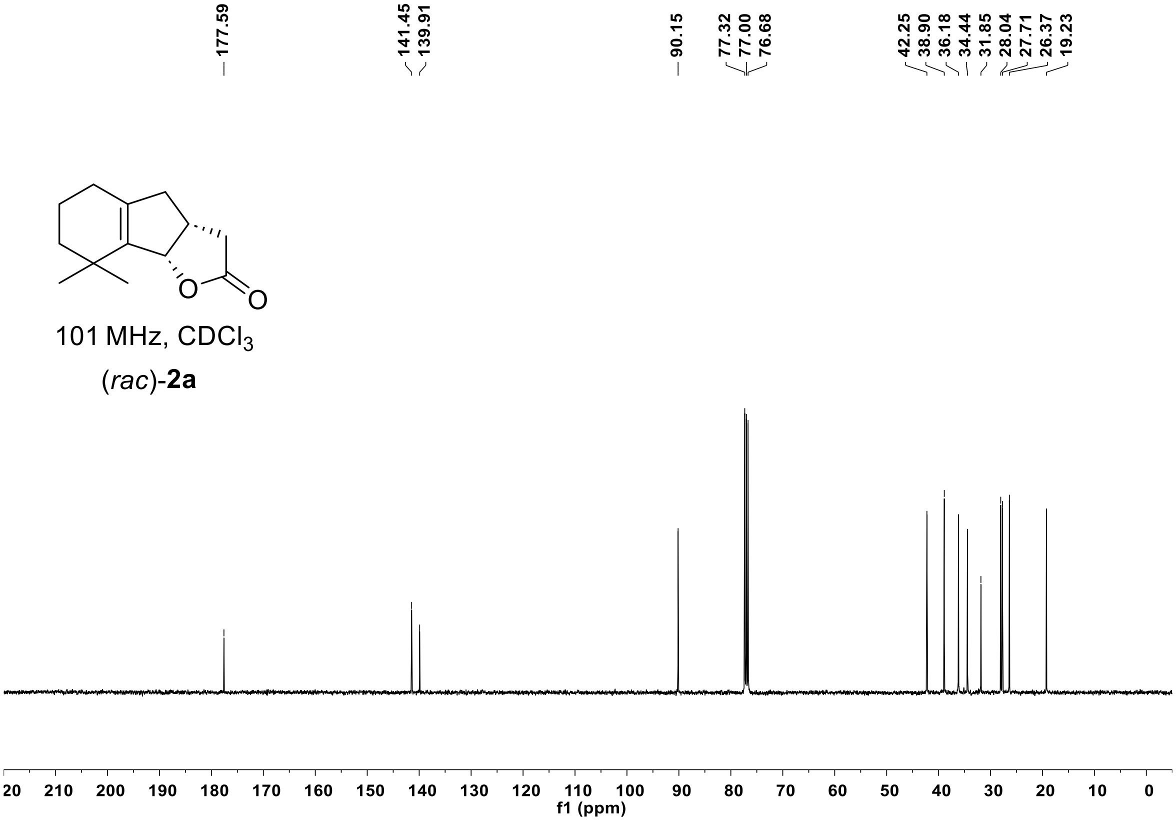
177.59
141.45
139.91
90.15
77.32
77.00
76.68
42.25
38.90
36.18
34.44
31.85
28.04
27.71
26.37
19.23
101 MHz, $CDCl_3$
(*rac*)-**2a**
f1 (ppm)

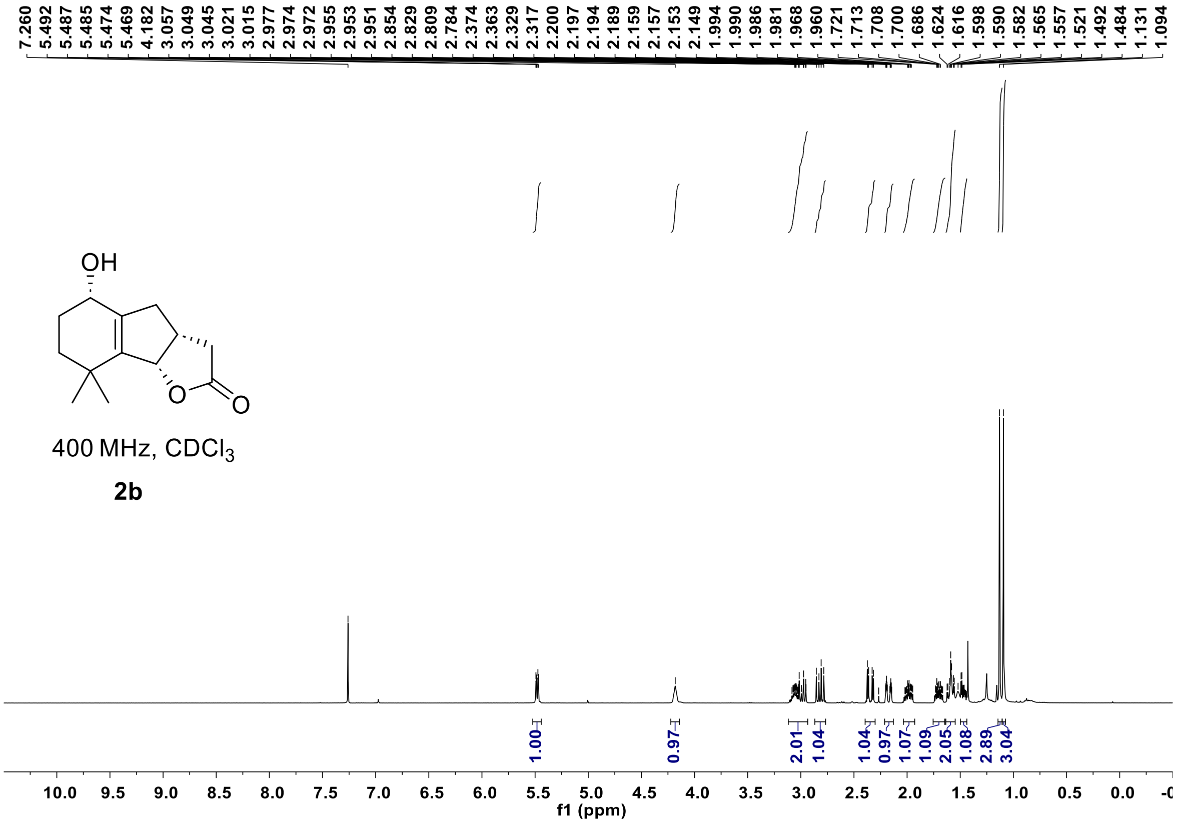
7.260 5.492 5.487 5.485 5.474 5.469 4.182 3.057 3.049 3.045 3.021 3.015 2.977 2.974 2.972 2.955 2.953 2.951 2.854 2.829 2.809 2.784 2.374 2.363 2.329 2.317 2.200 2.197 2.194 2.189 2.159 2.157 2.153 2.149 1.994 1.990 1.986 1.981 1.968 1.960 1.721 1.713 1.708 1.700 1.686 1.624 1.616 1.598 1.590 1.582 1.565 1.557 1.521 1.492 1.484 1.131 1.094
OH
O
O
400 MHz, $CDCl_3$
2b
1.00 0.97 2.01 1.04 1.04 0.97 1.07 1.09 2.05 1.08 2.89 3.04
10.0 9.5 9.0 8.5 8.0 7.5 7.0 6.5 6.0 5.5 5.0 4.5 4.0 3.5 3.0 2.5 2.0 1.5 1.0 0.5 0.0
f1 (ppm)

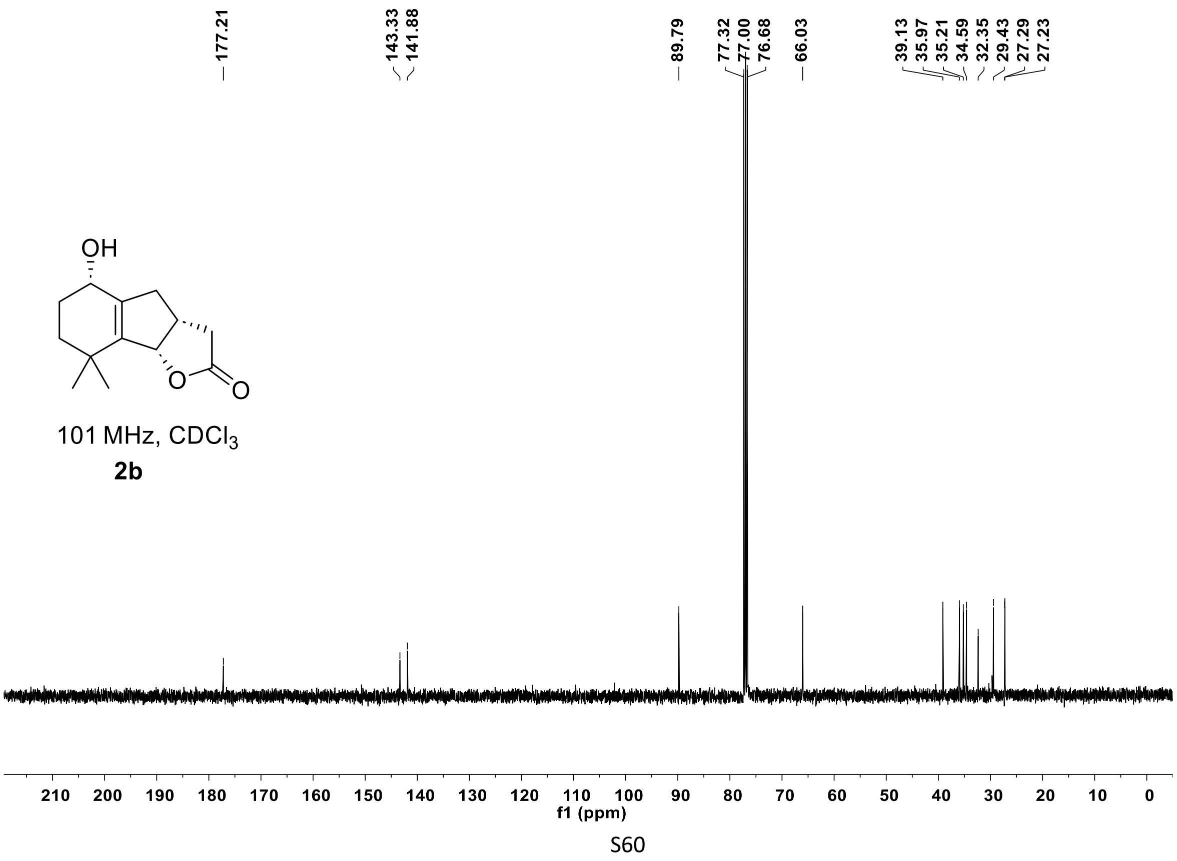
177.21
143.33 141.88
89.79
77.32 77.00 76.68
66.03
39.13 35.97 35.21 34.59 32.35 29.43 27.29 27.23
OH
O
O
101 MHz, $CDCl_3$
2b
210 200 190 180 170 160 150 140 130 120 110 100 90 80 70 60 50 40 30 20 10 0
f1 (ppm)

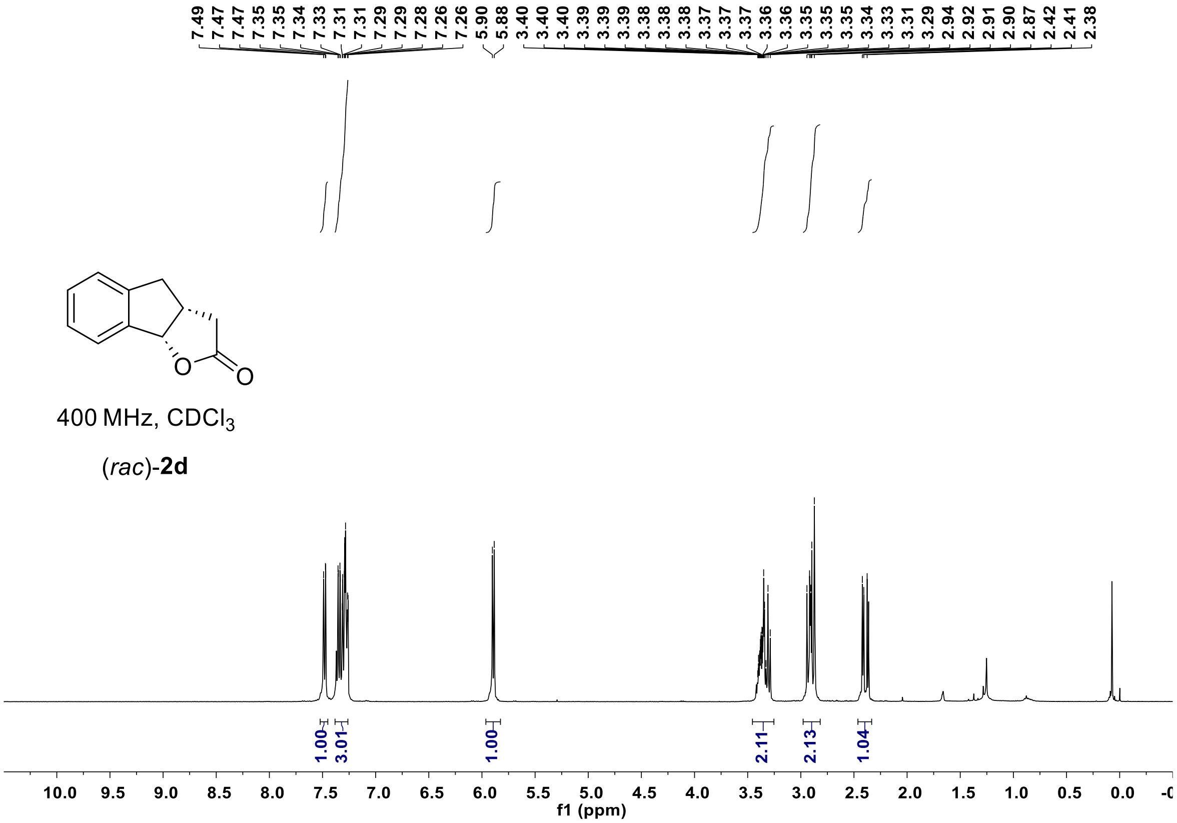
400 MHz, $CDCl_3$
(*rac*)-**2d**
f1 (ppm)

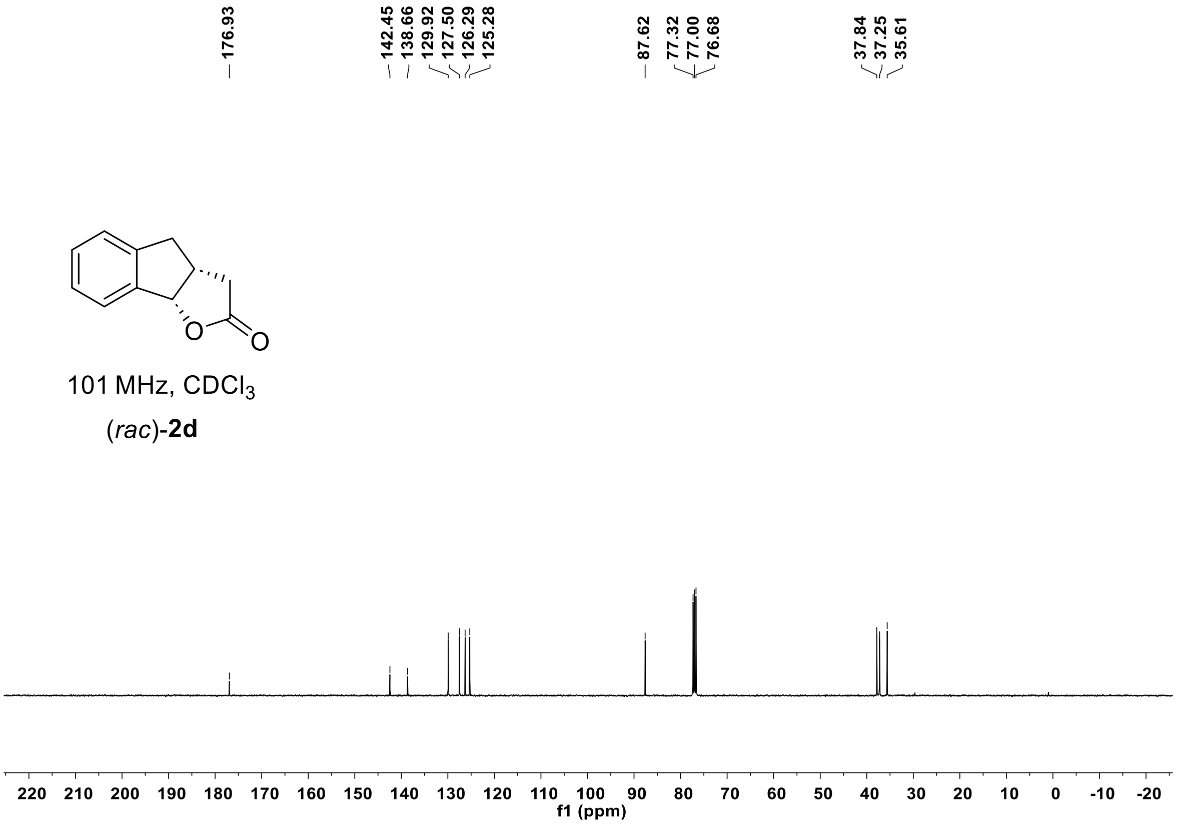
101 MHz, $CDCl_3$
(*rac*)-**2d**
f1 (ppm)

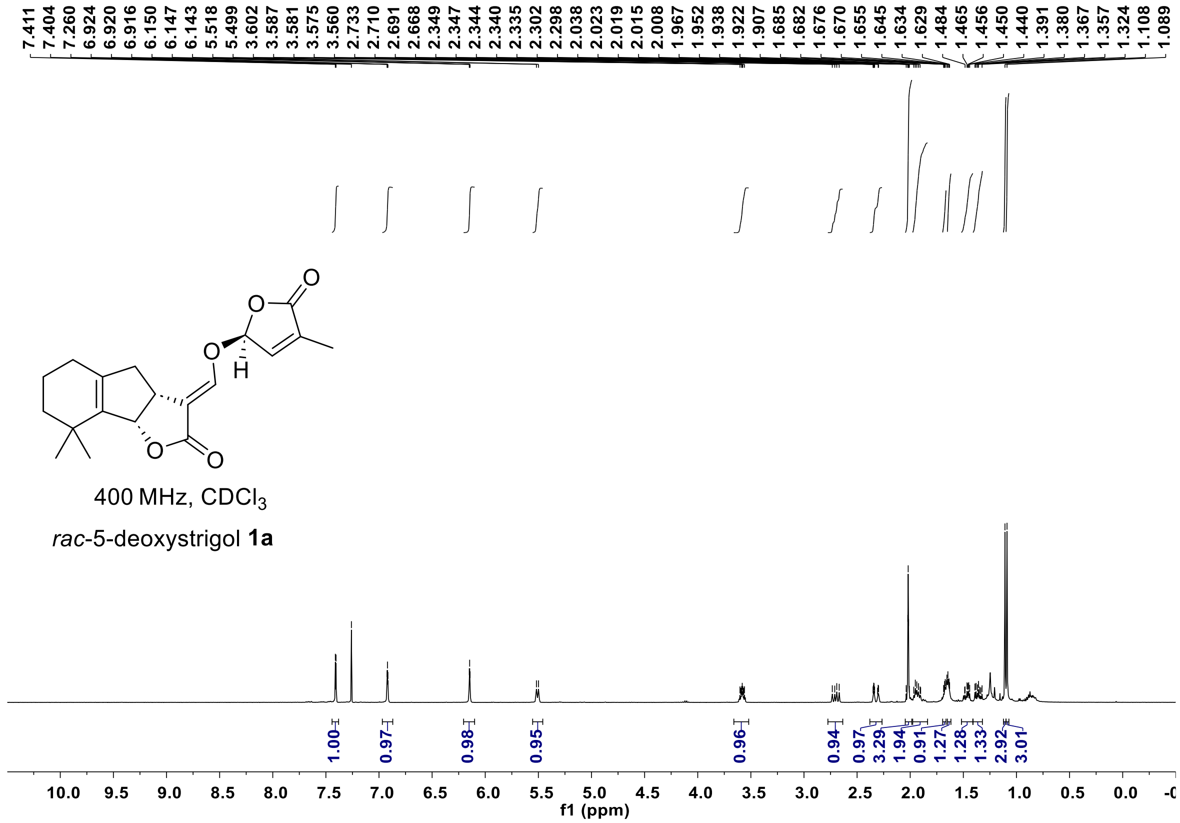
7.411 7.404 7.260 6.924 6.920 6.916 6.150 6.147 6.143 5.518 5.499 3.602 3.587 3.581 3.575 3.560 2.733 2.710 2.691 2.668 2.349 2.347 2.344 2.340 2.335 2.302 2.298 2.038 2.023 2.019 2.015 2.008 1.967 1.952 1.938 1.922 1.907 1.685 1.682 1.676 1.670 1.655 1.645 1.634 1.629 1.484 1.465 1.456 1.450 1.440 1.391 1.380 1.367 1.357 1.324 1.108 1.089
400 MHz, $CDCl_3$
rac-5-deoxystrigol **1a**
1.00 0.97 0.98 0.95 0.96 0.94 0.97 3.29 1.94 0.91 1.27 1.28 1.33 2.92 3.01
10.0 9.5 9.0 8.5 8.0 7.5 7.0 6.5 6.0 5.5 5.0 4.5 4.0 3.5 3.0 2.5 2.0 1.5 1.0 0.5 0.0
f1 (ppm)

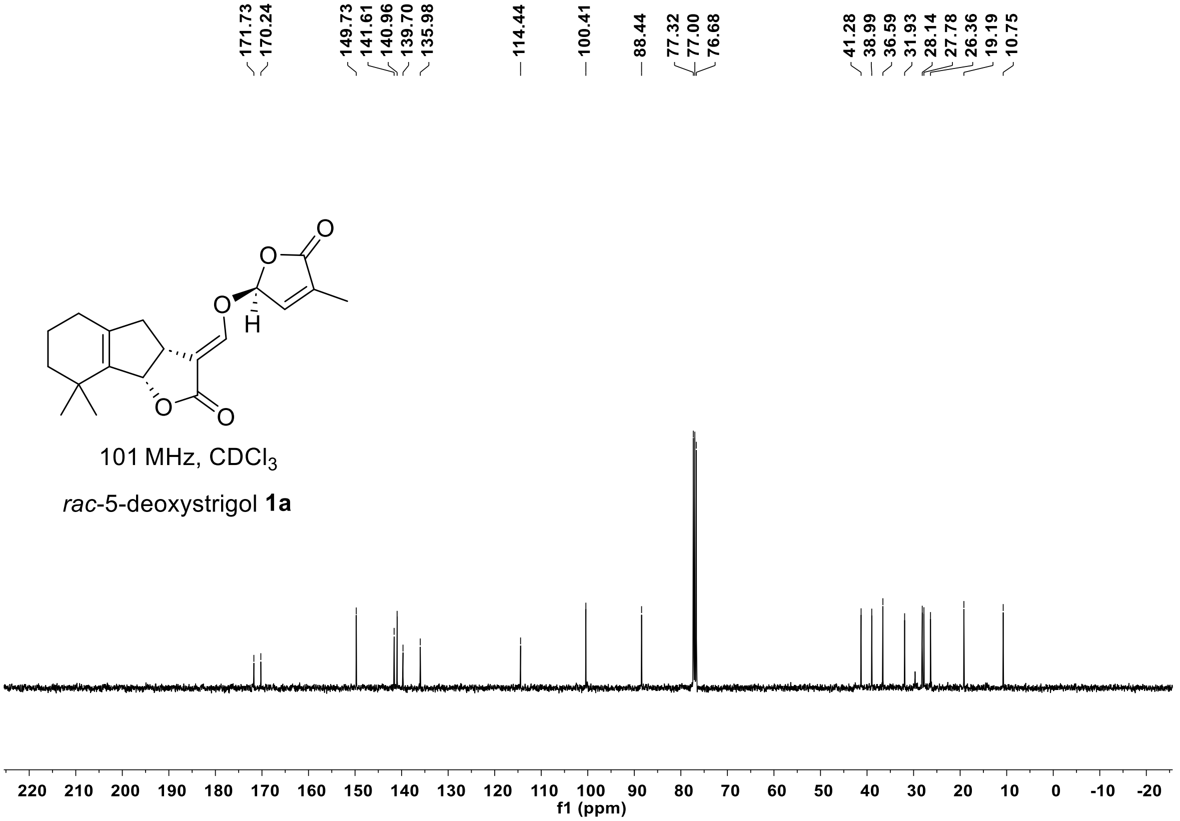
171.73 170.24 149.73 141.61 140.96 139.70 135.98 114.44 100.41 88.44 77.32 77.00 76.68 41.28 38.99 36.59 31.93 28.14 27.78 26.36 19.19 10.75
101 MHz, $CDCl_3$
rac-5-deoxystrigol **1a**
220 210 200 190 180 170 160 150 140 130 120 110 100 90 80 70 60 50 40 30 20 10 0 -10 -20
f1 (ppm)

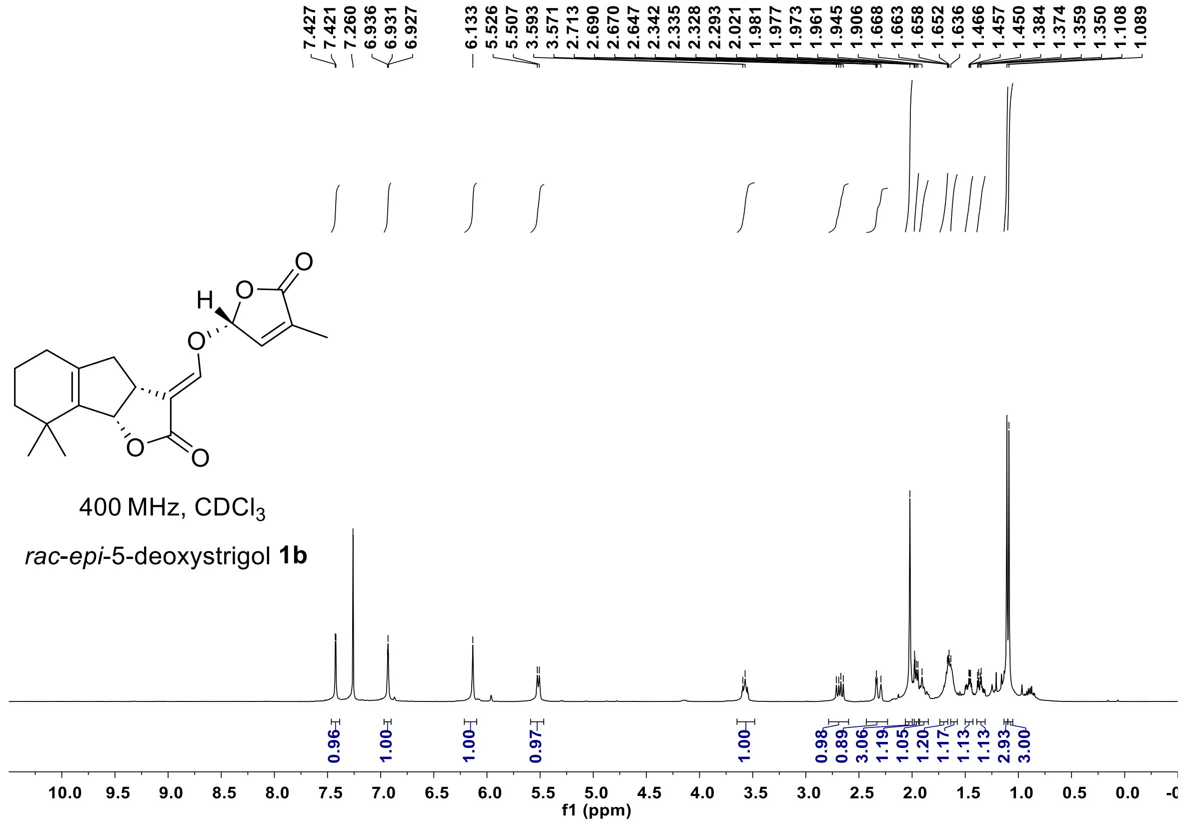
400 MHz, CDCl3
rac-epi-5-deoxystrigol 1b
f1 (ppm)

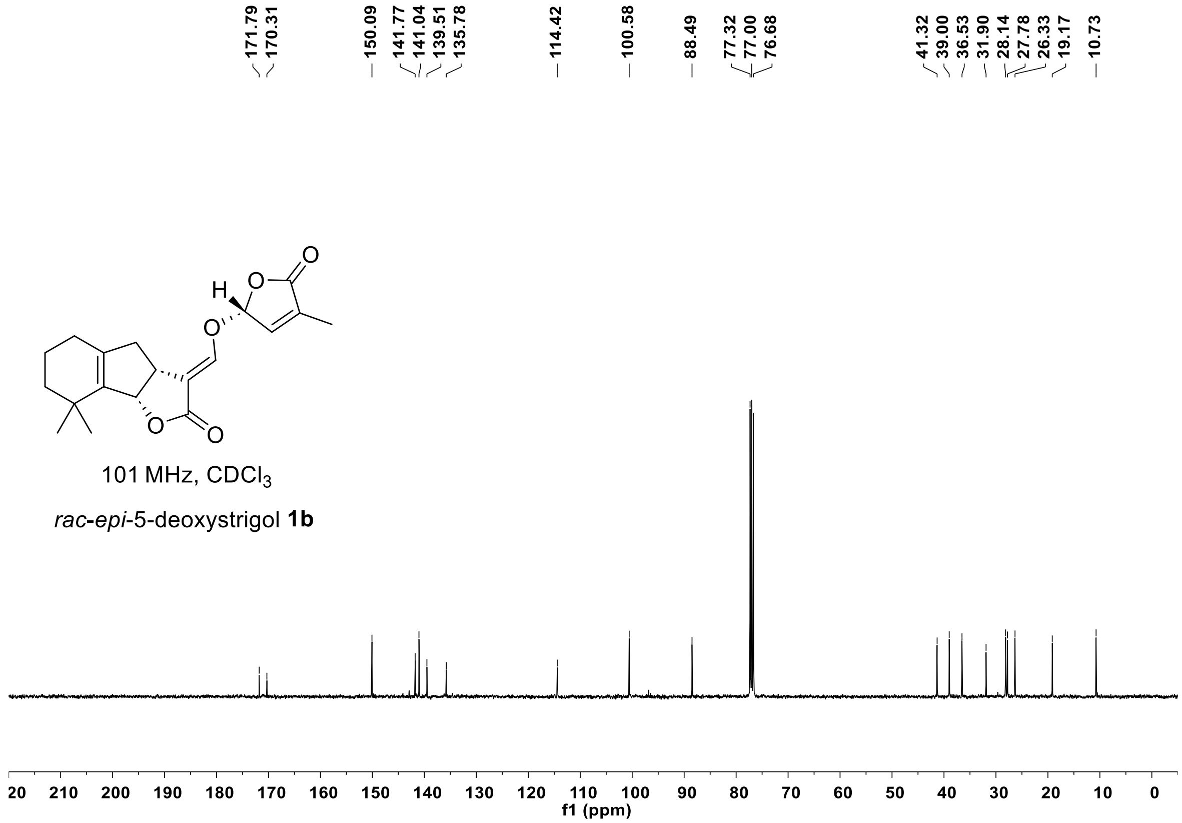
101 MHz, CDCl3
rac-epi-5-deoxystrigol 1b
f1 (ppm)

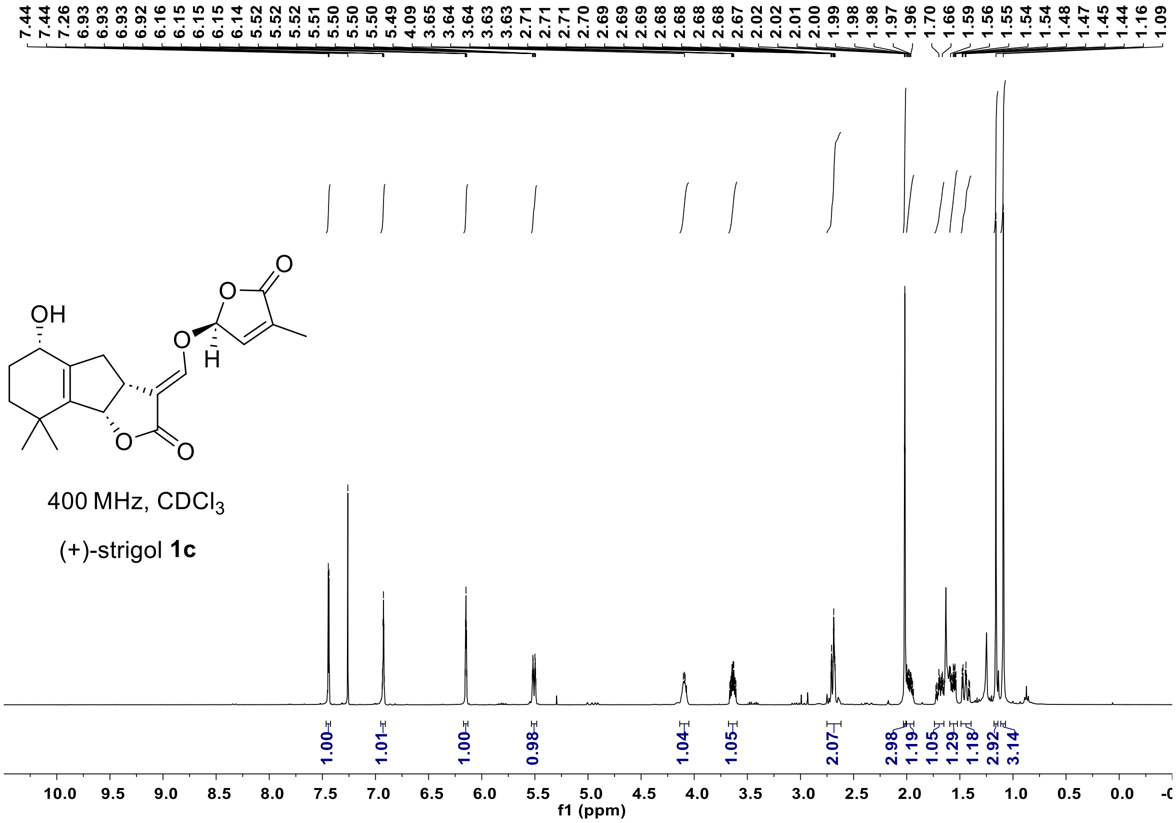
7.44 7.44 7.26 6.93 6.93 6.93 6.92 6.16 6.15 6.15 6.15 6.14 5.52 5.52 5.52 5.51 5.50 5.50 5.50 5.49 4.09 3.65 3.64 3.64 3.63 3.63 2.71 2.71 2.70 2.69 2.69 2.69 2.68 2.68 2.68 2.68 2.67 2.02 2.02 2.01 2.00 1.99 1.98 1.98 1.97 1.96 1.70 1.66 1.59 1.56 1.55 1.54 1.54 1.48 1.47 1.45 1.44 1.16 1.09
OH
O
O
O
H
O
O
400 MHz, CDCl3
(+)-strigol 1c
1.00 1.01 1.00 0.98 1.04 1.05 2.07 2.98 1.19 1.05 1.29 1.18 2.92 3.14
10.0 9.5 9.0 8.5 8.0 7.5 7.0 6.5 6.0 5.5 5.0 4.5 4.0 3.5 3.0 2.5 2.0 1.5 1.0 0.5 0.0
f1 (ppm)

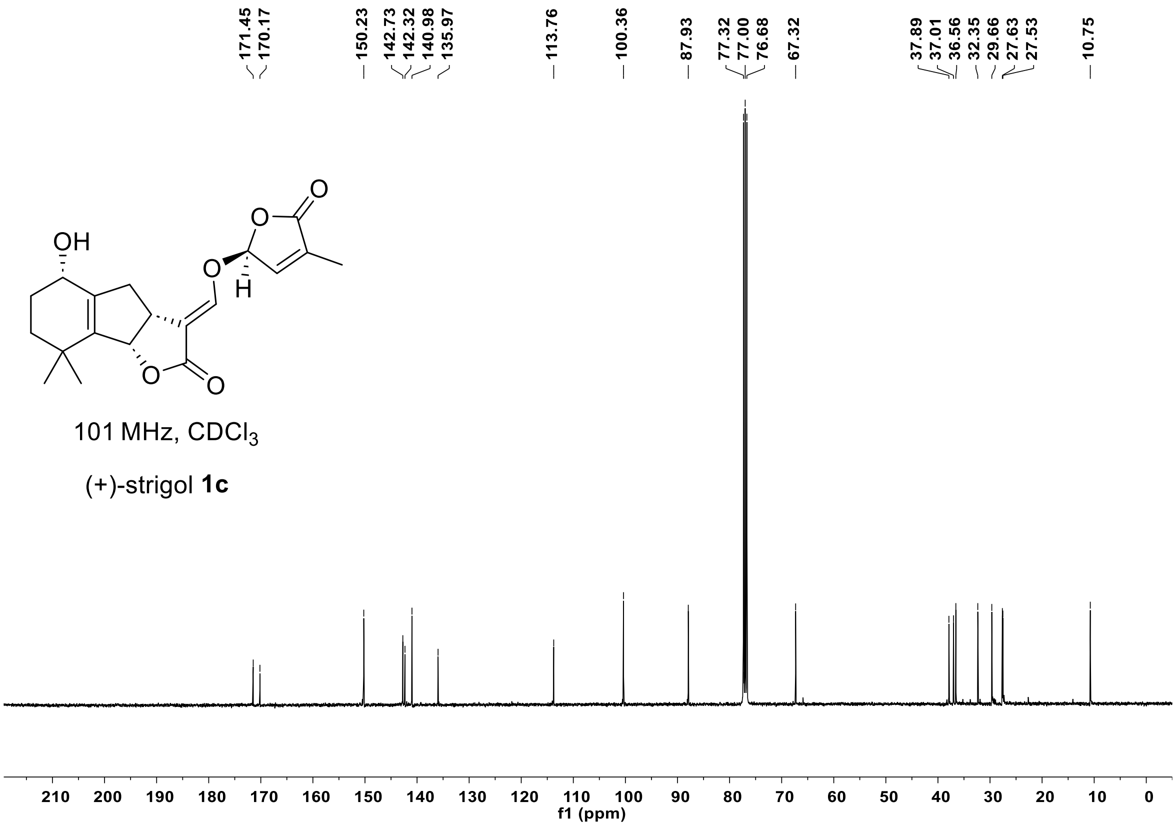
171.45 170.17 150.23 142.73 142.32 140.98 135.97 113.76 100.36 87.93 77.32 77.00 76.68 67.32 37.89 37.01 36.56 32.35 29.66 27.63 27.53 10.75
OH
O
O
O
H
O
O
101 MHz, CDCl3
(+)-strigol 1c
210 200 190 180 170 160 150 140 130 120 110 100 90 80 70 60 50 40 30 20 10 0
f1 (ppm)

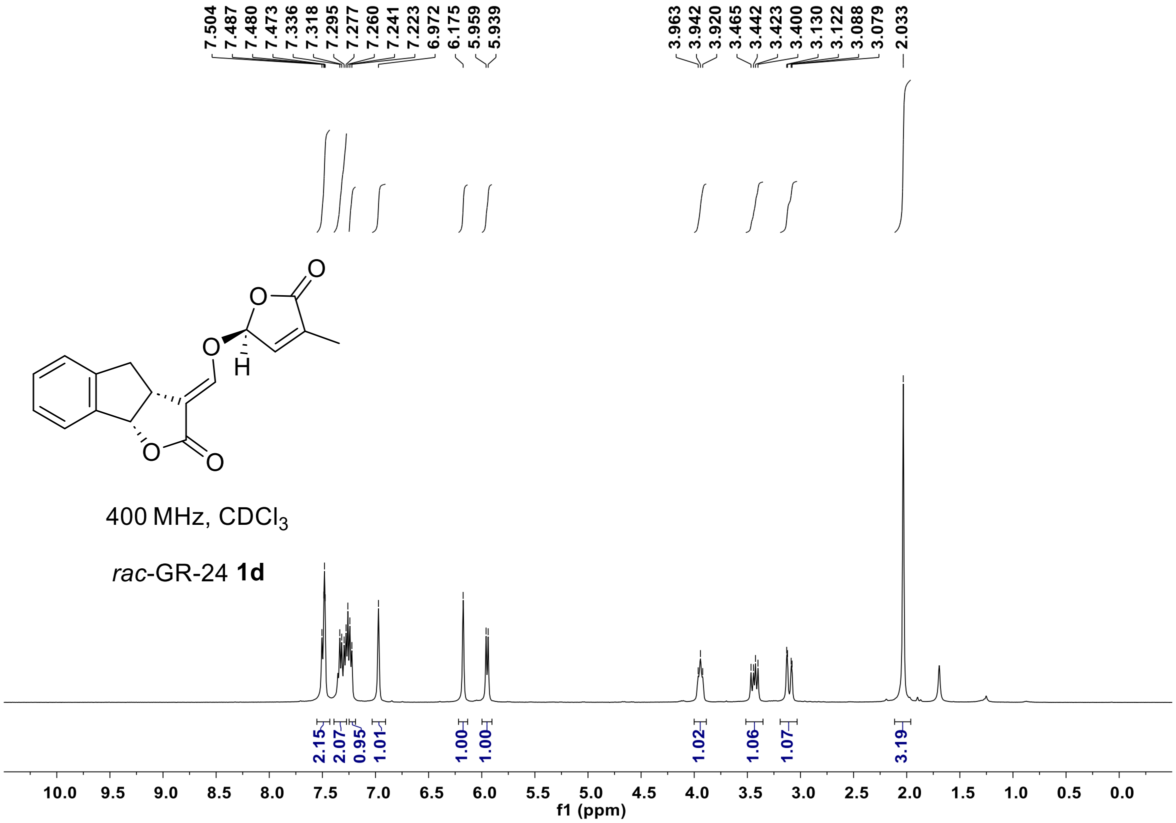
7.504
7.487
7.480
7.473
7.336
7.318
7.295
7.277
7.260
7.241
7.223
6.972
6.175
5.959
5.939
3.963
3.942
3.920
3.465
3.442
3.423
3.400
3.130
3.122
3.088
3.079
2.033
O
O
O
H
O
O
O
400 MHz, CDCl$_3$
rac-GR-24 **1d**
2.15
2.07
0.95
1.01
1.00
1.00
1.02
1.06
1.07
3.19
10.0 9.5 9.0 8.5 8.0 7.5 7.0 6.5 6.0 5.5 5.0 4.5 4.0 3.5 3.0 2.5 2.0 1.5 1.0 0.5 0.0
f1 (ppm)

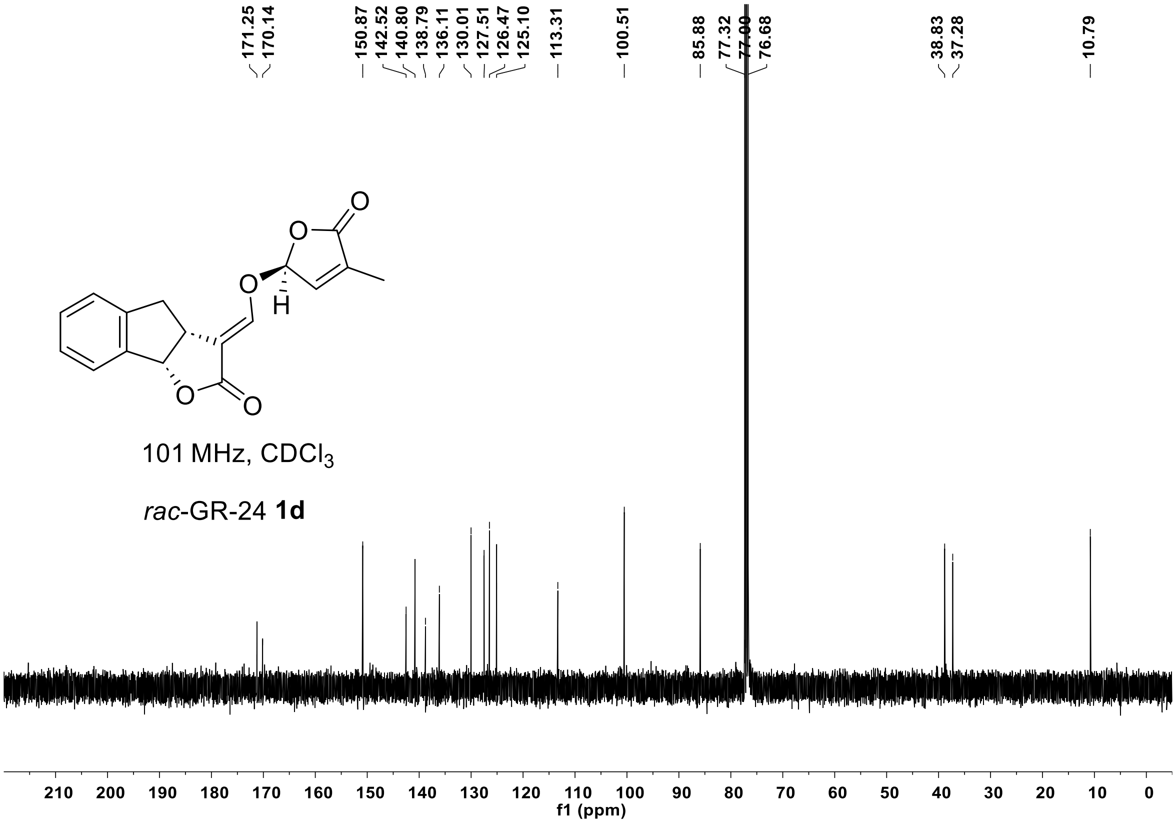
171.25
170.14
150.87
142.52
140.80
138.79
136.11
130.01
127.51
126.47
125.10
113.31
100.51
85.88
77.32
77.00
76.68
38.83
37.28
10.79
O
O
O
H
O
O
O
101 MHz, CDCl$_3$
rac-GR-24 **1d**
210 200 190 180 170 160 150 140 130 120 110 100 90 80 70 60 50 40 30 20 10 0
f1 (ppm)

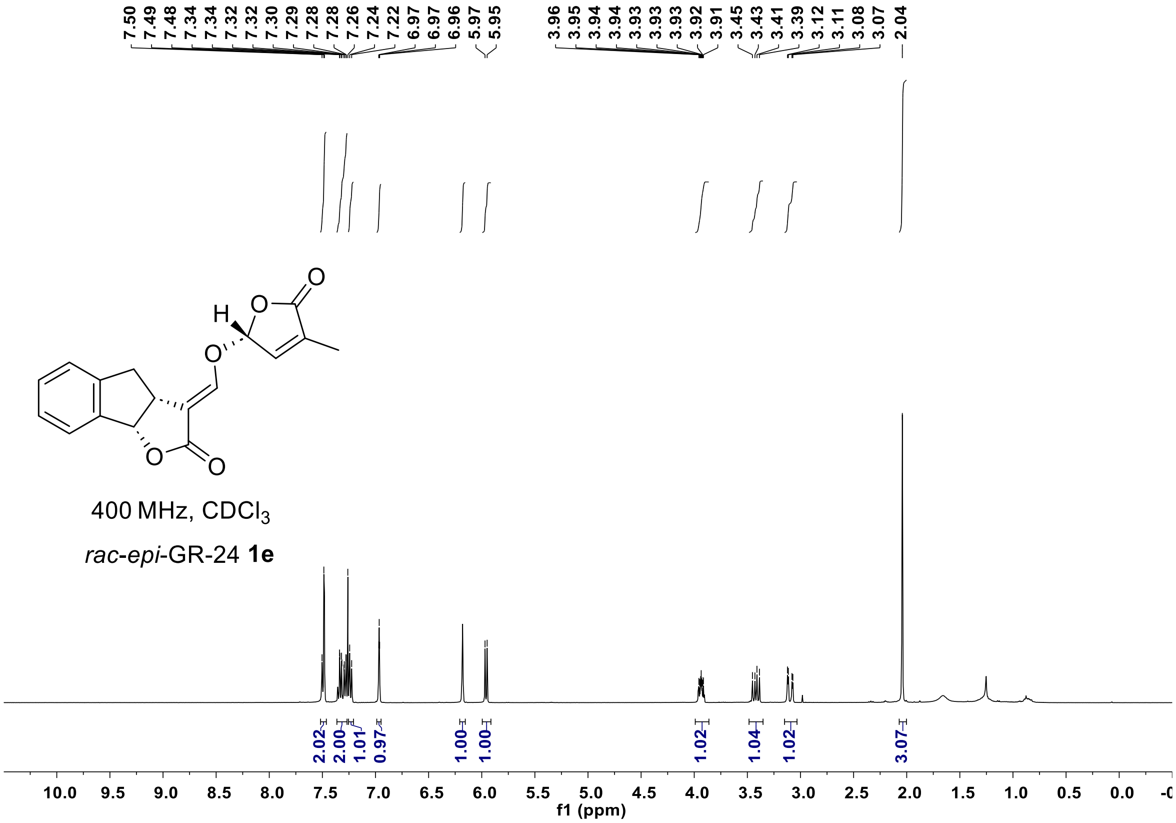

7.50
7.49
7.48
7.34
7.34
7.32
7.32
7.30
7.29
7.28
7.28
7.26
7.24
7.22
6.97
6.97
6.96
5.97
5.95
3.96
3.95
3.94
3.93
3.93
3.93
3.93
3.92
3.91
3.45
3.43
3.41
3.39
3.12
3.11
3.08
3.07
2.04
400 MHz, CDCl3
rac-epi-GR-24 1e
2.02
2.00
1.01
0.97
1.00
1.00
1.02
1.04
1.02
3.07
f1 (ppm)

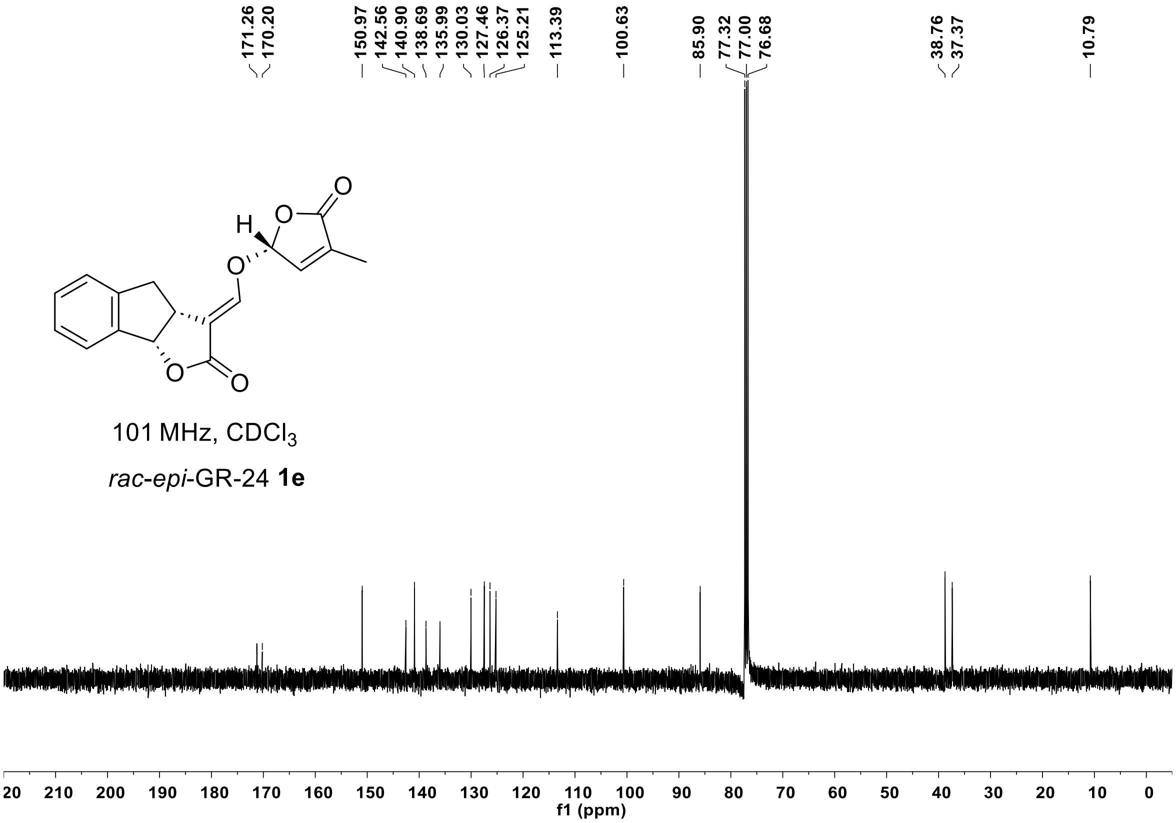

171.26
170.20
150.97
142.56
140.90
138.69
135.99
130.03
127.46
126.37
125.21
113.39
100.63
85.90
77.32
77.00
76.68
38.76
37.37
10.79
101 MHz, CDCl3
rac-epi-GR-24 1e
f1 (ppm)

6. References

(1) Zhu, C.; Yang, B.; Jiang, T.; Bäckvall, J. E. Olefin-Directed Palladium-Catalyzed Regio- and Stereoselective Oxidative Arylation of Allenes. *Angew. Chem. Int. Ed.* **2015**, *54* (31), 9066–9069.

(2) Zhu, C.; Yang, B.; Bäckvall, J.-E. Highly Selective Cascade C–C Bond Formation via Palladium- Catalyzed Oxidative Carbonylation–Carbocyclization–Carbonylation–Alkynylation of Enallenes. *J. Am. Chem. Soc.* **2015**, *137* (37), 11868–11871.

(3) Yang, B.; Qiu, Y.; Jiang, T.; Wulff, W. D.; Yin, X.; Zhu, C.; Bäckvall, J.-E. E. Enantioselective Palladium-Catalyzed Carbonylative Carbocyclization of Enallenes via Cross-Dehydrogenative Coupling with Terminal Alkynes: Efficient Construction of α-Chirality of Ketones. *Angew. Chem. Int. Ed.* **2017**, *56* (16), 4535–4539.

(4) Qiu, Y.; Yang, B.; Zhu, C.; Bäckvall, J. -E. Palladium-Catalyzed Oxidative Carbocyclization-Borylation of Enallenes to Cyclobutenes. *Angew. Chem. Int. Ed.* **2016**, *55* (22), 6520–6524.

(5) Zheng, W.; Zhang, W.; Huang, C.; Wu, P.; Qian, H.; Wang, L.; Guo, Y.; Ma, S. Tetrasubstituted Allenes via the Palladium-Catalysed Kinetic Resolution of Propargylic Alcohols Using a Supporting Ligand. *Nat. Catal.* **2019**, *2*, 997–1005.

(6) Zhu, C.; Yang, B.; Qiu, Y.; Bäckvall, J. -E. Olefin-Directed Palladium-Catalyzed Regio- and Stereoselective Hydroboration of Allenes. *Chem. Eur. J.* **2016**, *22* (9), 2939–2943.

(7) Chen, C.; Fujimoto, Y.; Girdaukas, G.; Sih, C. J. Quantitative Analyses of Biochemical Kinetic Resolutions of Enantiomers. *J. Am. Chem. Soc.* **1982**, *104* (25), 7294–7299.

(8) Brooks, D. W.; Bevinakatti, H. S.; Kennedy, E.; Hathaway, J. Practical Total Synthesis of (±)-Strigol. *J. Org. Chem.* **1985**, *50* (5), 628–632.

(9) Shoji, M.; Suzuki, E.; Ueda, D. Total Synthesis of (±)-5-Deoxystrigol via Reductive Carbon−Carbon Bond Formation. *J. Org. Chem.* **2009**, *74* (10), 3966–3969.

(10) Bromhead, L. J.; Visser, J.; McErlean, C. S. P. Enantioselective Synthesis of the Strigolactone Mimic (+)-GR24. *J. Org. Chem.* **2014**, *79*, 1516−1520.
